# Ten-Step Total Synthesis of (±)-Phaeocaulisin A Enabled by Cyclopropanol Ring-Opening Carbonylation

**DOI:** 10.1021/jacs.4c12121

**Published:** 2024-11-12

**Authors:** Chang Liu, Mingyu Zhang, Lidan Zeng, Yong Wan, Mingji Dai

**Affiliations:** † Department of Chemistry, Emory University, Atlanta, Georgia 30322, United States; ‡ Department of Pharmacology and Chemical Biology, School of Medicine, Emory University, Atlanta, Georgia 30322, United States

## Abstract

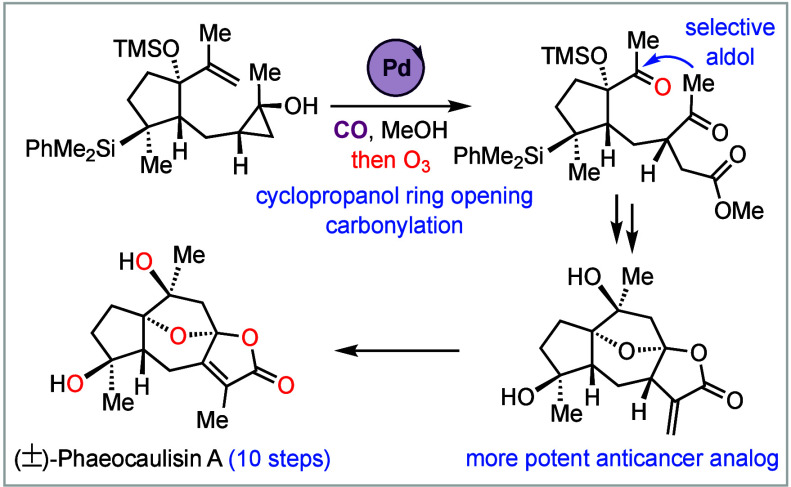

We report an efficient total synthesis of (±)-phaeocaulisin A, a guaianolide sesquiterpene natural product possessing a complex tetracyclic skeleton embedded with an oxaspirolactone and a fused bicyclic lactone, four oxygen-containing stereocenters, and an 8-oxabicyclo[3.2.1]octane core. Our synthesis features a novel palladium-catalyzed cyclopropanol ring-opening carbonylation to access a key γ-ketoester, a chemo- and stereoselective aldol cyclization to form the seven-membered carbocycle, and a cascade ketalization–lactonization to construct the desired tetracyclic skeleton. With these strategically important C–C and C–O bond formation transformations, a 10-step total synthesis of (±)-phaeocaulisin A was achieved. We further developed the cyclopropanol ring-opening carbonylation chemistry to provide an alternative approach to prepare γ-ketoesters. Biologically, the penultimate intermediate with an α-methylene γ-butyrolactone moiety was identified as a promising lead compound with anticancer proliferation activity against a panel of triple-negative or HER2+ breast cancer cell lines.

Guaianolides are a large group of sesquiterpene lactones of chemotaxonomic and biological importance. Guaianolide natural products contain two main subfamilies, 8,12-guaianolides (Scheme 1A, **1**) and 6,12-guaianolides (**2**), based on the connection pattern of their 5/7/5-tricyclic skeleton.^1^ Phaeocaulisins A (**3**), B (**4**), and H (**5**) and related analogs belong to the 8,12-guaianolide subfamily and were isolated from *Curcuma phaeocaulis* by Qiu, Zhao, and co-workers in 2013.^2^ Phaeocaulisin A has an unusual and highly oxygenated tetracyclic skeleton featuring an 8-oxabicyclo[3.2.1]octane core. Within its tetracyclic skeleton, there exist an oxaspirolactone, a fused bicyclic lactone, and four oxygen-containing stereocenters (C1, C4, C8, and C10). Biologically, phaeocaulisin A was identified as a promising noncytotoxic anti-inflammatory agent showing inhibitory effects on lipopolysaccharide-induced nitric oxide production in RAW 264.7 macrophages with an IC_50_ value at 1.5 μM.^2a,2b^ Preliminary structure–activity relationship studies indicated that its unique 8-oxabicyclo[3.2.1]octane core may account for the superior bioactivity of phaeocaulisin A over the rest of its family members without such a bridged ring system. Additionally, phaeocaulisin A demonstrated antiproliferation activity against A375 human melanoma cells at 5 and 10 μM concentrations.^2c,2d^ All of these suggest that phaeocaulisin A is a promising lead compound for developing cancer and autoimmune disease treatment and is worth further investigation.

**Scheme 1:**
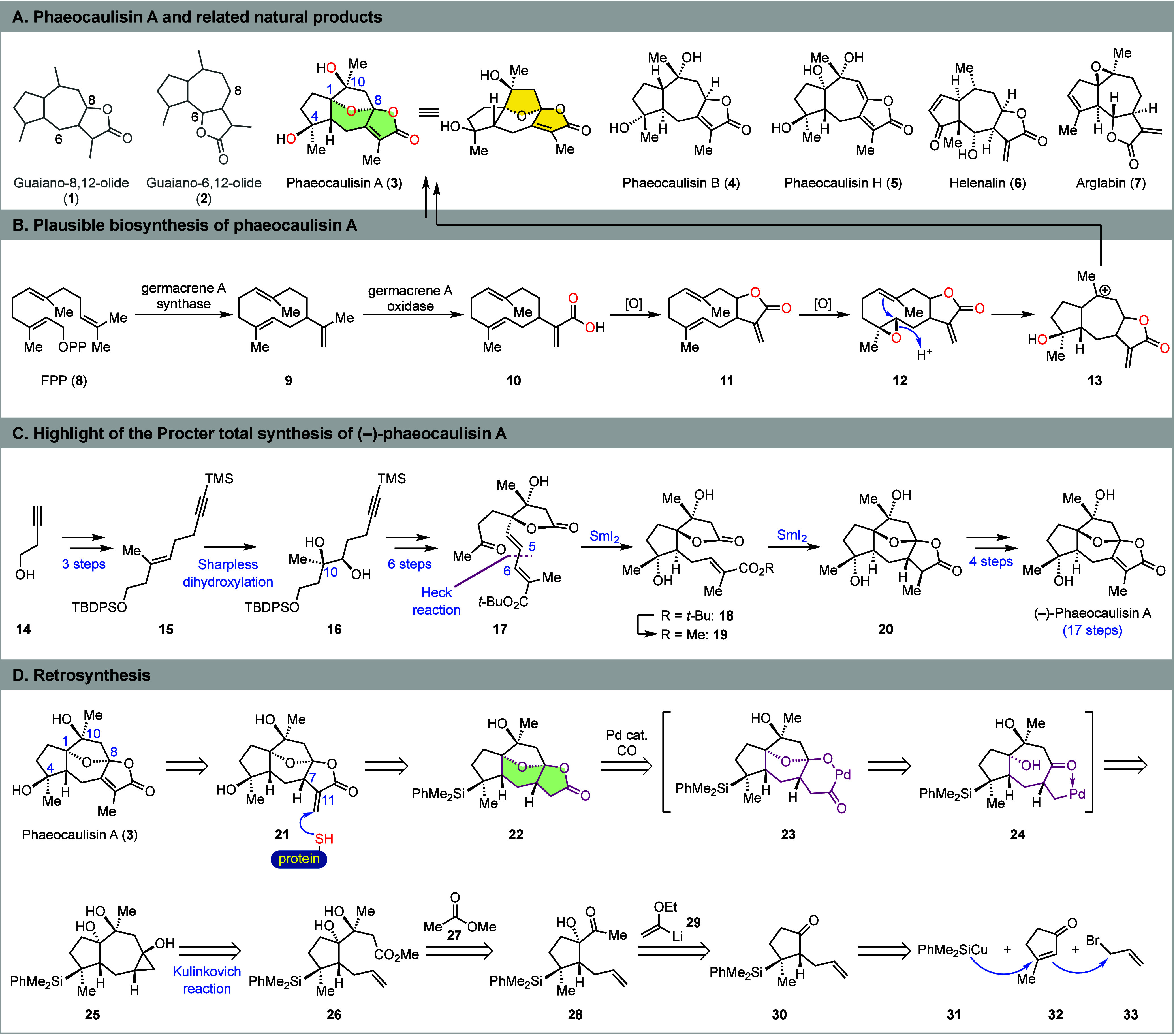
Structure, Plausible Biosynthesis, Prior Total Synthesis, and Retrosynthetic Analysis of Phaeocaulisin A

Biosynthetically, phaeocaulisins are derived from farnesyl diphosphate (FPP) via key intermediates including germacrene A (**9**) and germacrolide (**11**) (Scheme 1B).^1,3^ An enzymatic epoxidation may convert **11** to its 4,5-epoxide (**12**), which could then undergo a cationic transannular cyclization to form the guaianolide skeleton (**13**), a plausible precursor of phaeocaulisin A and related natural products.

Although significant progresses have been made in the field of guaianolide total synthesis,^4^ there is only one reported total synthesis of phaeocaulisin A (Scheme 1C). In 2022, Procter and co-workers achieved an elegant total synthesis of (−)-phaeocaulisin A in 17 longest linear sequence (LLS) steps from 3-butyn-1-ol (**14**).^5^ They first prepared dienolate **17** in 10 steps, including a Sharpless dihydroxylation to install the C10 tertiary alcohol and control its absolute stereochemistry and a Heck reaction to form the C5–C6 bond. They then used two highly efficient samarium(II) iodide-mediated reductive cyclizations (**17** → **18** and **19** → **20**) to deliver advanced tetracyclic intermediate **20**, en route to (−)-phaeocaulisin A, the enantiomer of the naturally occurring one.

We have been innovating catalytic carbonylation methodologies and strategies to facilitate complex natural product total synthesis.^6^ In particular, we have developed palladium-catalyzed hydroxycyclopropanol ring-opening carbonylative lactonizations to synthesize both oxaspirolactones^7^ and fused bicyclic lactones.^8^ We took note of phaeocaulisin A because of the oxaspirolactone and fused bicyclic lactone moieties embedded in its tetracyclic ring system. Retrosynthetically (Scheme 1D), with our continued interest in protein covalent modification,^9^ we envisioned **21** with an α-methylene γ-butyrolactone moiety as an advanced intermediate. Such an α-methylene γ-butyrolactone moiety is frequently found in many guaianolide sesquiterpenes such as helenalin (**6**)^10^ and arglabin (**7**)^11^ with a broad range of biological activities and can potentially form a covalent bond with certain cellular proteins.^12^ Thus, we were curious if **21** would be more biologically active than **3** or exhibit new biological functions because of its α-methylene γ-butyrolactone moiety. The α-methylene γ-butyrolactone of **21** could be isomerized to the desired endocyclic α,β-unsaturated γ-butyrolactone of **3**.^13^ Notably, Procter and co-workers have reached the C7 epimer of **21**, but failed to isomerize it to phaeocaulisin A,^5^ indicating that such isomerization may not be straightforward. Compound **21** could be synthesized from **22** via a sequence of Fleming–Tamao oxidation^14^ and an α-methylenation reaction. We then wondered about the possibility of building the fused bicyclic lactone moiety of **22** via the palladium-catalyzed cyclopropanol ring-opening carbonylative lactonization we developed previously.^7,8^ Thus, intermediate **25** was designed. We were hoping that a sequence of C–C cleavage, acetal formation, and carbonylative lactonization (**25** → **24** → **23** → **22**) would convert **25** to **22**. To access **25**, we proposed an intramolecular Kulinkovich reaction^15^ of **26** (or its lactone form) to construct the 7,3-fused ring system.^16^ Compound **26** could be accessed from **28** via a chelation-controlled 1,2-addition with an enolate derived from **27**. Compound **28** could be quickly assembled from commercially available 3-methyl-2-cyclopentenone **32** via conjugate silyl addition, α-allylation, and 1,2-addition.

Our synthesis commenced with **32**, which was converted to **30** in 74% yield as a single diastereomer using a conjugate silyl addition followed by trapping the enolate with allyl bromide (Scheme 2A).^17^ Subsequent 1,2-addition of (1-ethoxyvinyl)lithium **29** to **30** gave α-hydroxyketone **28** in 69% yield after a one-pot hydrolysis of the enol ether. The subsequent aldol reaction of **28** with the lithium enolate derived from **27** occurred smoothly, but the initial aldol product underwent spontaneous lactonization to afford spirocyclic lactone **34** (CCDC 2292481) in 58% yield. Unfortunately, various Kulinkovich cyclopropanol synthesis conditions failed to produce cyclopropanol **36** from **34** or its TMS ether **35** despite the proximity of the lactone and the terminal olefin as indicated by the crystal structure of **34** (see the Supporting Information).

**Scheme 2:**
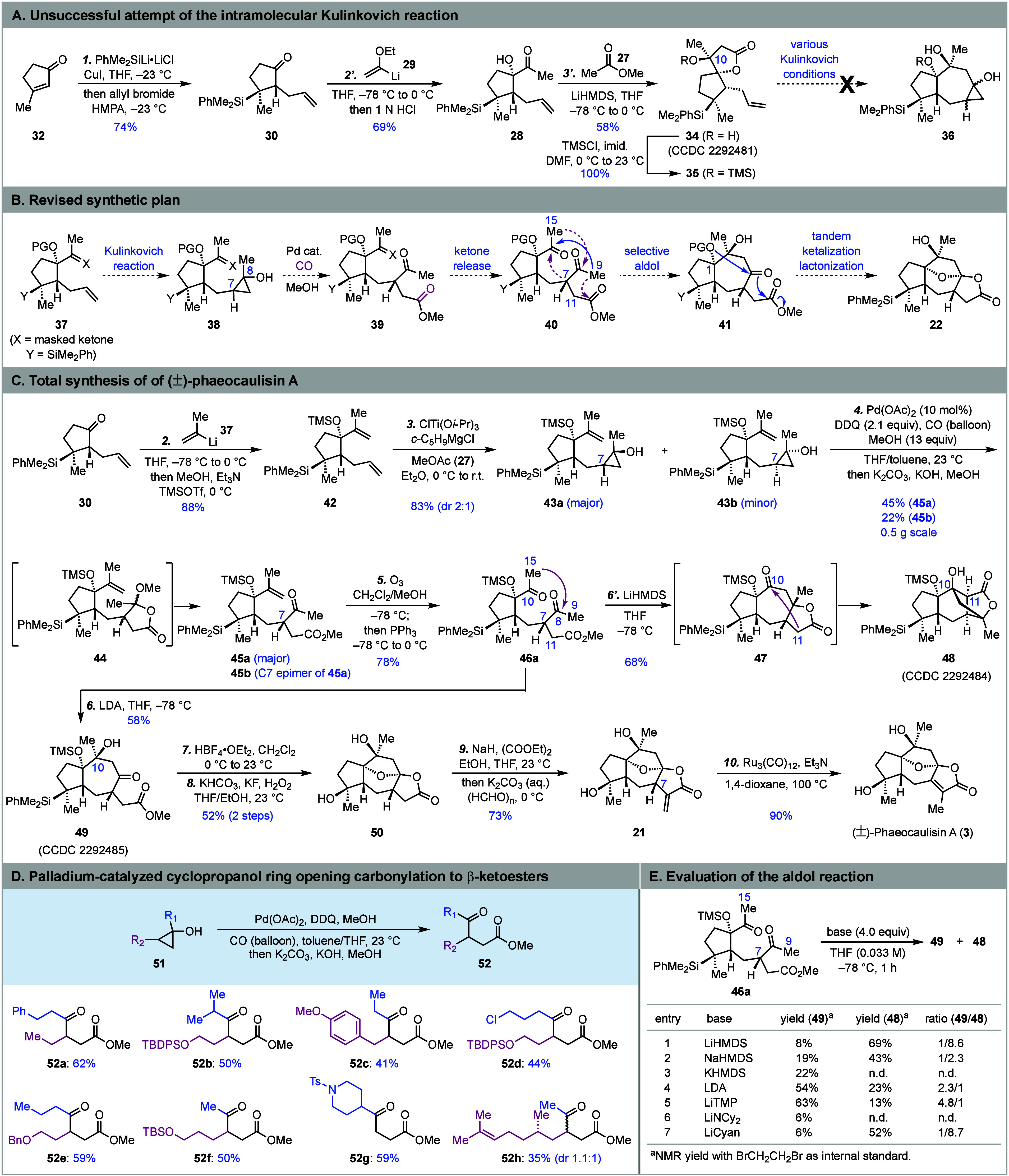
Total Syntheses of (±)-Phaeocaulisin A and Cyclopropanol Ring-Opening Carbonylation to γ-Ketoesters

We then proposed an intermolecular Kulinkovich reaction^18^ to synthesize cyclopropanol **38** from **37** (Scheme 2B). A cyclopropanol ring-opening carbonylation would convert **38** to γ-ketoester **39**. A subsequent intramolecular aldol reaction was envisioned to close the seven-membered ring and produce **41**. After removal of the protecting group on the C1 tertiary alcohol, tandem ketalization–lactonization should deliver the same advanced tetracyclic intermediate **22**. Several challenges associated with this new approach should be noted. First, it would be difficult to control the stereochemical outcome of the intermolecular Kulinkovich reaction. Second, there was no report about unprotected cyclopropanol ring-opening carbonylation to γ-ketoesters.^19^ In 1980, Murai and co-workers reported a synthesis of γ-ketoesters from siloxycyclopropanes,^20^ which requires one equivalent of toxic Hg(OAc)_2_ and one equivalent of PdCl_2_. Third, there are three carbonyl functionalities (two methyl ketones and one ester) and four nucleophilic sites (C9, C15, C7, C11) in **40**, and how to ensure the desired intramolecular aldol reaction was considered as a big challenge because there are several competitive aldol or Claisen reaction pathways, three of which are indicated by the dashed arrows.

In the revised approach (Scheme 2C), **42** was prepared from **30** via a 1,2-addition with **37** followed by one-pot TMS protection. Following Cha and co-workers’ protocol,^18c^ a mixture (2:1) of cyclopropanols **43a** and **43b** was synthesized from **42** in 83% yield, which was then treated with an excess amount of Pd(OAc)_2_ together with triethylamine in methanol under a carbon monoxide balloon. We were delighted to isolate a mixture containing γ-ketoester **45a** as the major product (see the Supporting Information). To render this reaction catalytic, we identified DDQ as an external oxidant, which allows the use of 10 mol % of Pd(OAc)_2_. Interestingly, under the catalytic conditions, acetal lactone **44** was identified as the primary carbonylation product. A subsequent one-pot alkaline workup with a mixture of K_2_CO_3_ and KOH was needed to open the lactone and convert **44** to a 2:1 separable mixture of γ-ketoesters **45a** and **45b** (the C7-epimer of **45a**, not shown) in total 67% yield. To evaluate the generality of this transformation, eight other cyclopropanols, **51a**–**51h**, were prepared and subjected to the same one-pot reaction conditions to give γ-ketoesters **52a**–**52h** in moderate yields (Scheme 2D).

After establishing the carbonylation method to prepare **45a**, the isopropenyl group was converted to the required methyl ketone via ozonolysis and **46a** was produced in 78% yield. We then focused on the aldol cyclization to close the seven-membered ring.^21^ When **46a** was treated with LiHMDS in THF at –78 °C, to our surprise compound **48** (CCDC 2292484) with an unprecedented tetracyclic skeleton was obtained as the major product in 68% yield. The formation of **48** was proposed to start with one of the undesired aldol cyclizations between C15 and C8. The primary aldol product then reacted with the ester group to form tricyclic lactone **47**. Upon further deprotonation at the α-position (C11) of the lactone, a second intramolecular aldol reaction occurred between C11 and C10 to produce **48**. Additionally, the desired product **49** could be observed by crude NMR as the minor product. After evaluating a list of bases (Scheme 2E), LDA and LiTMP were identified as proper bases to produce desired product **49** (CCDC 2292485) as a major product in 54% and 63% NMR yield, respectively, on a small scale, together with **48** as a minor product. While slightly higher yield and selectivity were obtained with LiTMP at the exploration stage, the use of LDA worked better for scale up. The selectivity change is presumably due to the stronger basicity of LDA and LiTMP, which prefer deprotonation at the C9 position first. Notably, the C7 stereochemistry influences the aldol cyclization significantly as well. When the C7 epimer of **46a** (**46b**) was used for the aldol cyclization, six-membered ring closure with a bond formation between C11 and C10 started to dominate (see the Supporting Information).

With **49** in hand, we then removed the TMS group on the angular hydroxy group with fluoroboric acid to trigger a spontaneous ketalization and lactonization process to form the oxa bridge and γ-butyrolactone in one step. Under the same reaction conditions, electrophilic cleavage of the Si–Ph bond occurred as well. The resulting tetracyclic intermediate with a silyl fluoride was next subjected to Fleming–Tamao oxidation with hydrogen peroxide and potassium fluoride to give **50** in 52% yield over two steps. To introduce the exocyclic methylene moiety, we first explored the protocols of using Eschenmoser’s salt^22^ and were unsuccessful. We then examined a one-pot α-carboxylation (NaH, (COOEt)_2_) and α-hydroxymethylation (K_2_CO_3_, (HCHO)_*n*_) followed by elimination to install the α-methylene group^23^ and were able to isolate **21** in 73% yield. To our delight, final ruthenium-catalyzed alkene migration^13^ of **21** successfully gave (±)-phaeocaulisin A in 90% yield. Compound **21** and its C7 epimer displayed very different reactivities, as Procter and co-workers were not able to isomerize the latter to phaeocaulisin A.^5^ Overall, starting from 3-methyl-2-cyclopentenone, (±)-phaeocaulisin A was prepared in 10 steps.

With a concise total synthesis of phaeocaulisin A established, we started to assess its anticancer activity. We were also interested in **21** because of its α-methylene γ-butyrolactone moiety, as it has been found in plenty of anticancer natural products and could function as a reactive site for protein covalent modification. Advanced intermediate **50** was included for comparison, as well. Given the challenges and limited options for treating triple-negative breast cancer (TNBC) and HER2+ breast cancer, we evaluated the effects of (±)-phaeocaulisin A (**3**), **21**, and **50** on a panel of TNBC or HER2+ breast cancer cell lines: MDA-MB-468 (TNBC), SKBR3 (HER2+), and MDA-MB-231 (TNBC). The assessment was performed by using a colony formation assay to measure their effects on cell survival and proliferation. Our result revealed that compound **21** is significantly more potent than both phaeocaulisin A (**3**) and **50**, leading to a substantial reduction of cell survival. In contrast, phaeocaulisin A (**3**) and **50** did not show significant differences across these cell lines (Figure 1A–C). Compound **21** exhibits strong inhibitory effects, with IC_50_ values of 0.248 μM, 1.161 μM, and 2.693 μM for MDA-MB-468, SKBR3, and MDA-MB-231, respectively (Figure 1D). Notably, **21** remains effective against MDA-MB-231, a cell line resistant to phaeocaulisin A (**3**) and **50** (Figure 1C). These findings support our hypothesis that the α-methylene γ-butyrolactone moiety enhances the biological activity of **21**, which could be a promising lead compound for cancer therapy.

**Figure 1:**
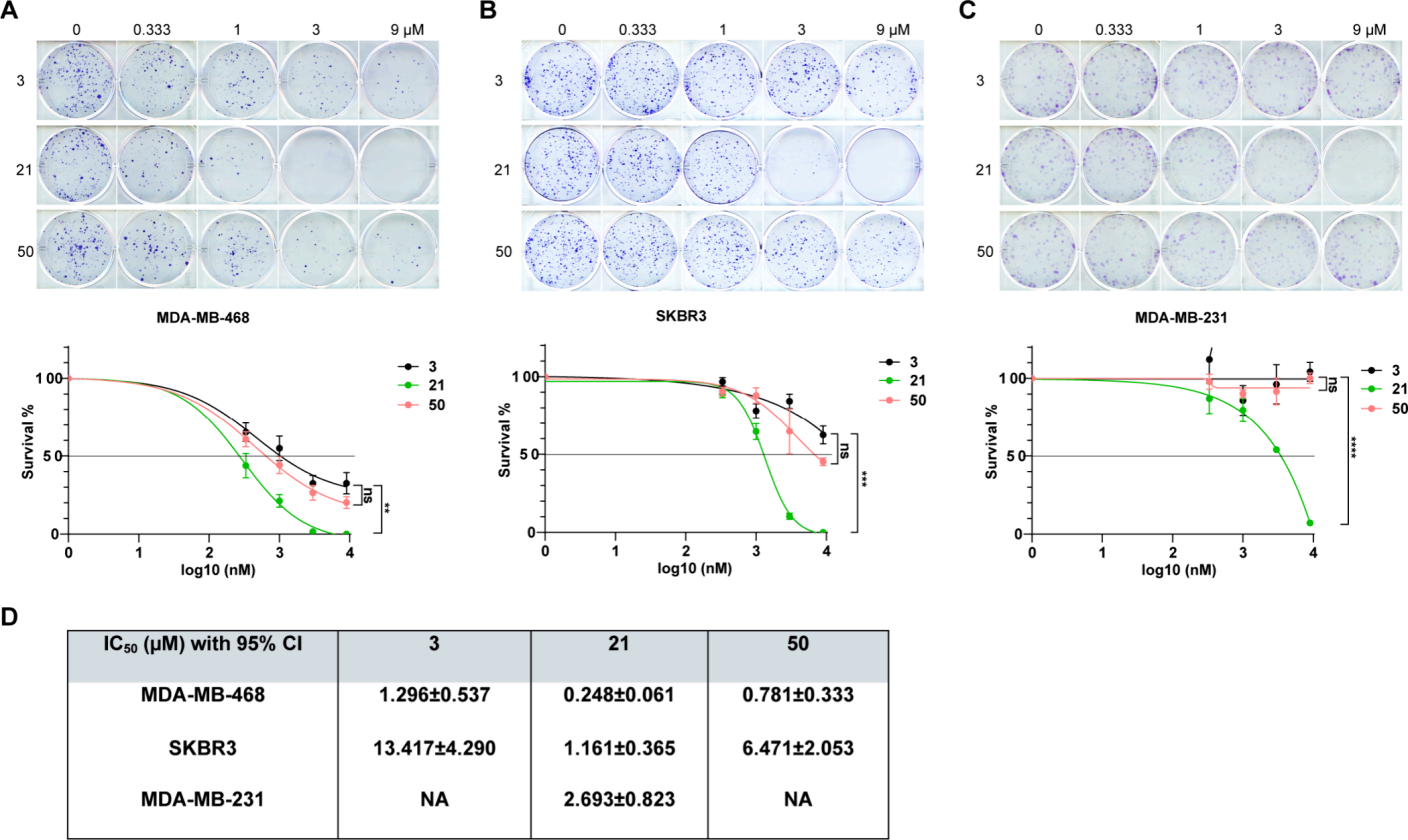
Inhibitory effects of (±)-phaeocaulisin A (**3**), **21**, and **50** on cancer cell survival. (A–C) Representative images of the colony formation of MDA-MB-468 (A), SKBR3 (B), and MDA-MB-231(C) under the treatment of different compounds (top) and the corresponding dose–response curve (bottom). **p < 0.01, ***p < 0.001, ****p < 0.0001, and ns indicates not significant by Student’s *t* test. Data (mean ± SEM) are representative of three independent experiments. (D) The summary of the IC_50_. CI: confidence interval. NA: not available. IC_50_ of **3** and **50** for MDA-MB-231 is not available, as they did not show inhibitory effects within the range of the concentration tested.

In summary, we completed a concise total synthesis of (±)-phaeocaulisin A from commercially available 3-methyl-2-cyclopentenone. We developed a novel palladium-catalyzed cyclopropanol ring-opening carbonylation to access a key γ-ketoester and expand the portfolio of palladium-catalyzed cyclopropanol ring-opening chemistry.^24^ From the γ-ketoester, we realized a chemo- and stereoselective intramolecular aldol reaction to close the seven-membered ring and a cascade ketalization–lactonization reaction to build the tetracyclic core. These enabling transformations allowed us to complete a 10-step total synthesis of (±)-phaeocaulisin A in 3.8% total yield. Further biological evaluation of phaeocaulisin A and its analogs **21** and **50** against a panel of triple-negative or HER2+ breast cancer cell lines identified **21** with an α-methylene γ-butyrolactone moiety as a promising lead compound for further development.

## Supporting Information Available

Experimental procedures and NMR spectra for all new compounds (PDF)

## ABBREVIATIONS

TMS: trimethylsilyl
HMPA: hexamethylphosphoramide
DDQ: 2,3-dichloro-5,6-dicyano-1,4-benzoquinone
THF: tetrahydrofuran
Li/Na/KHMDS: lithium/sodium/potassium bis(trimethylsilyl)amide
LDA: lithium diisopropylamide
LiTMP: lithium tetramethylpiperidide
LiNCy_2_: lithium dicyclohexylamide
LiCyan: lithium *N*-cyclohexyl anilide
TNBC: triple-negative breast cancer.
