# Supplementary material for: Ten-Step Total Synthesis of (±)-Phaeocaulisin A Enabled by Cyclopropanol Ring-Opening Carbonylation
Source: J Am Chem Soc. 2024 Nov 12;146(47):32276–82. doi: 10.1021/jacs.4c12121 (PMC11613322; doi:10.1021/jacs.4c12121)
Supplement: Supplementary file 1 — ja4c12121_si_001.pdf [file ja4c12121_si_001.pdf]

Supporting Information

Ten-Step Total Synthesis of (±)-Phaeocaulisin A Enabled by Cyclopropanol Ring-Opening Carbonylation

Chang Liu,[1] Mingyu Zhang,[1] Lidan Zeng,[2] Yong Wan,*[,2] and Mingji Dai*[,1,2]

[1]Department of Chemistry, Emory University, Atlanta, GA 30322, United States

[2]Department of Pharmacology and Chemical Biology, School of Medicine, Emory University, Atlanta, GA 30322, United States

Table of Contents

Part 1. Experimental procedures and spectra data..S3

Part 2. X-ray structures and analysis data...S32

Part 3. ^{1}H and ^{13}C NMR spectra...S36

Part 1. Experimental procedures and spectra data.

General Methods. NMR spectra were recorded on Bruker spectrometers (^1H at 400 MHz, 600 MHz, 800 MHz and ^{13}C at 101, 151, 201 MHz). Chemical shifts (δ) were given in ppm with reference to solvent signals [^1H NMR: $CDCl_3$ (7.26), CD_3OD (3.31); ^{13}C NMR: $CDCl_3$ (77.2), CD_3OD (49.0)]. All reactions were carried out under an inert argon atmosphere with dry solvents under anhydrous conditions unless otherwise stated. Tetrahydrofuran (THF), toluene (PhMe), dichloromethane (DCM) and dimethylformamide (DMF) were dried by passage through activated alumina columns under an argon atmosphere in a solvent purification system. All other solvents and reagents were used as obtained from Sigma Aldrich, Fisher Scientific, and Oakwood without further purification. Reactions were monitored by thin layer chromatography (TLC), using UV light as the visualizing agent and/or cerium molybdate (CAM) and heat as a developing agent. Column chromatography was performed on silica gel. Room temperature (rt) is around 23 °C.

1.1 Total Synthesis of (±)-Phaeocaulisin A

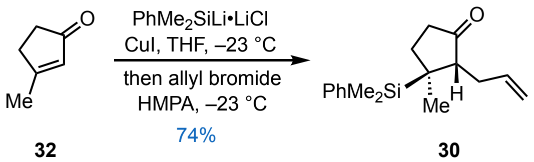

32 30

Compound 30 was prepared according to a known procedure with modifications. [1]

Preparation of $PhSiMe_2Li·LiCl$ solution: Under argon, THF (96 mL) was added to a flame-dried flask charged with lithium metal (1.8 g, 0.26 mol). Then chloro(dimethyl)phenylsilane (10 mL, 60 mmol) was added dropwise (a water bath was used to maintain the reaction temperature). The reaction mixture was stirred overnight and used without titration.

Under argon, the above $PhSiMe_2Li·LiCl$ solution (*calc.* 2.3 equiv., 100 mL, *calc.* 56 mmol) was transferred to a flame-dried flask charged with copper(I) iodide (1.05 equiv., 4.8 g, 25 mmol) at –23 °C. After stirring at –23 °C for 3 h, 3-methyl-2-cyclopentenone (**32**, 1.0 equiv., 2.4 mL, 24 mmol) was added at –23 °C, and the stirring continued for 1 hour. Then a solution of allyl bromide (4.1 equiv., 8.6 mL, 100 mmol) in HMPA (48 mL) was added at –23 °C. After the reaction was complete (~1 hour), saturated NH_4Cl solution (48 mL) was added to the reaction

mixture and the desired organic material was extracted with ethyl acetate (100 mL×3). The combined organic layer was washed with NH_4Cl solution (×3) then brine (×3), dried over sodium sulfate, filtered, and concentrated under reduced pressure.

The above reaction was carried out twice, and the combined residue was purified by silica gel column chromatography (hexanes to 5% EtOAc in hexanes) to give ketone **30** (9.76 g, 74% in average) as a pale-yellow liquid.

Note: The original paper [1] only reported a low-resolution proton NMR spectrum of **30** (in CCl_4). We reported its detailed characterization data.

Compound 30.

^{1}H NMR (600 MHz, $CDCl_3$) δ 7.54–7.51 (m, 2H), 7.42–7.35 (m, 3H), 5.97 (dddd, J = 17.1, 10.1, 8.0, 5.7 Hz, 1H), 5.02–4.96 (m, 2H), 2.22–2.11 (m, 3H), 2.05 (dd, J = 9.0, 3.1 Hz, 1H), 1.98–1.86 (m, 2H), 1.59 (ddd, J = 13.0, 6.5, 4.4 Hz, 1H), 0.92 (s, 3H), 0.39 (s, 3H), 0.38 (s, 3H).

^{13}C NMR (101 MHz, $CDCl_3$) δ 221.3, 137.9, 136.4, 134.7, 129.6, 128.0, 115.3, 55.9, 33.5, 30.5, 29.8, 29.5, 16.3, –5.0, –5.5.

IR (film): 3070, 2956, 2923, 2864, 1733, 1638, 1427, 1260, 1109, 814, 771, 737, 701 cm^{-1}.

HRMS (APCI): *m/z* Calc. for $C_{17}H_{25}OSi$ $[M+H]^+$: 273.1667, found: 273.1669;

TLC (9% EtOAc in hexanes): R_f = 0.46.

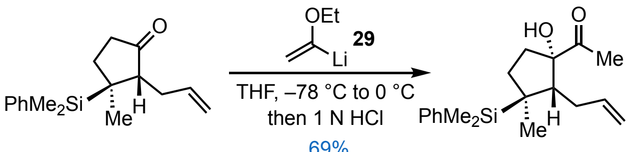

30 28

1,2-addition of 1-Ethoxyvinyllithium to 30.

Under argon, to a solution of ethyl vinyl ether (12.2 equiv., 2.3 mL, 24.2 mmol) in THF (12 mL) was added *tert*-butyl lithium (5.1 equiv., 1.7 M in THF, 5.9 mL, 10.0 mmol) at –78 °C. The reaction mixture was then stirred at 0 °C for 30 min. Subsequently, a solution of **30** (1.0 equiv., 540 mg, 2.0 mmol) in THF (9.0 mL) was added dropwise at –78 °C. The reaction mixture was stirred at –78 °C for 1.5 hours, then allowed to warm to 0 °C over 2.5 hours. After the reaction was complete (usually 1 hour at 0 °C), saturated NH_4Cl solution was added to the

reaction mixture and the desired organic material was extracted with ethyl acetate (50 mL×3). The combined organic layer was washed with brine, 1 N HCl then brine, dried over sodium sulfate, filtered, and concentrated under reduced pressure. The residue was purified by silica gel column chromatography (5% EtOAc in hexanes) to give ketone **28** (431 mg, 69%) as colorless oil.

Compound 28.

^{1}H NMR (400 MHz, CDCl$_3$) δ 7.57 (dd, *J* = 6.6, 3.0 Hz, 2H), 7.41–7.34 (m, 3H), 5.44 (ddt, *J* = 17.2, 10.0, 7.3 Hz, 1H), 4.92 (d, *J* = 17.2 Hz, 1H), 4.84 (d, *J* = 10.0 Hz, 1H), 4.00 (s, 1H), 2.29 (td, *J* = 12.6, 7.6 Hz, 1H), 2.14 (dd, *J* = 11.5, 2.8 Hz, 1H), 2.02–1.91 (m, 1H), 1.88 (s, 3H), 1.79 (dd, *J* = 13.6, 7.0 Hz, 1H), 1.69–1.53 (m, 3H), 1.09 (s, 3H), 0.38 (s, 6H).

^{13}C NMR (101 MHz, CDCl$_3$) δ 212.5, 137.3, 137.0, 134.6, 129.5, 127.9, 116.9, 88.6, 51.5, 38.6, 36.3, 31.0, 30.7, 23.5, 18.7, –5.2, –5.4.

HRMS (ESI): *m/z* Calc. for $C_{19}H_{29}O_2Si$ $[M+H]^+$: 317.1931, found: 317.1933.

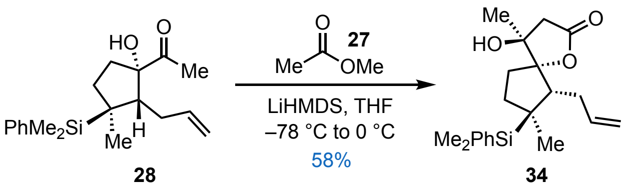

Condensation between 28 and methyl acetate.

Under argon, to a solution of LiHMDS (2.5 equiv., 1.0 M in THF, 1.5 mL, 1.5 mmol) in THF (12 mL) was added a solution of methyl acetate in THF (1.1 equiv., *V*:*V* = 1 : 3, 0.21 mL, 0.66 mmol) dropwise at –78 °C. The reaction mixture was stirred at –78°C for 1 hour, followed by the addition of a solution of **28** (1.0 equiv., 193 mg, 0.61 mmol) in THF (3.0 mL). The reaction mixture was stirred at -78 °C for 15 minutes then at 0 °C for 1 hour. NH_4Cl solution was added, and the resulting mixture was extracted with ethyl acetate (10 mL×3). The combined organic layer was washed with brine, dried over sodium sulfate, filtered, and concentrated under reduced pressure. The residue was purified by silica gel column chromatography (50% EtOAc in hexanes) to give lactone **34** (127 mg, 58%) as white solid.

Compound 34.

^{1}H NMR (400 MHz, CDCl$_3$) δ 7.53 (dd, *J* = 6.5, 3.0 Hz, 2H), 7.41–7.32 (m, 3H), 5.83 (dddd,

J = 17.3, 10.0, 8.8, 5.7 Hz, 1H), 5.12 (d, J = 17.3 Hz, 1H), 5.04 (d, J = 10.0 Hz, 1H), 2.61 (d, J = 17.2 Hz, 1H), 2.44 (d, J = 17.2 Hz, 1H), 2.33 (dt, J = 13.7, 9.3 Hz, 1H), 2.25 (dd, J = 9.7, 2.6 Hz, 1H), 2.19 (s, 1H), 2.01–1.87 (m, 2H), 1.84 (ddd, J = 13.7, 6.8, 2.3 Hz, 1H), 1.54 (td, J = 12.5, 6.8 Hz, 1H), 1.45 (ddd, J = 12.9, 7.5, 2.4 Hz, 1H), 1.05 (s, 3H), 1.01 (s, 3H), 0.37 (s, 3H), 0.36 (s, 3H).

^{13}C NMR (101 MHz, CDCl$_3$) δ 174.8, 139.7, 136.8, 134.6, 129.5, 128.0, 117.2, 100.6, 78.8, 45.3, 44.2, 35.5, 34.0, 31.8, 31.5, 20.5, 17.0, −5.1, −5.4.

IR (film): 3458, 2957, 2921, 1754, 1638, 1427, 1250, 1110, 828, 809, 770, 734, 702 cm^{-1}.

HRMS (ESI): *m/z* Calc. for $C_{21}H_{31}O_3Si$ [M+H]$^+$: 359.2037, found: 359.2038.

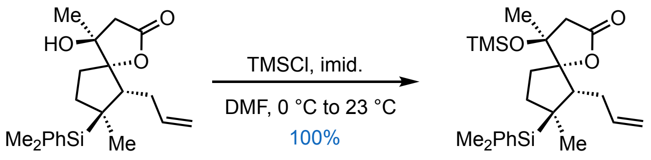

34 35

TMS protection of 34.

Under argon, to a solution of **34** (1 equiv., 72 mg, 0.20 mmol) and imidazole (20 equiv., 0.27 g, 4.0 mmol) in DMF (1.2 mL) was added chlorotrimethylsilane (10 equiv., 0.25 mL, 2.0 mmol) dropwise at 0 °C. The reaction mixture was slowly warmed to ambient temperature and stirred for 2 days. Methanol (0.08 mL) was added to quench the reaction. The reaction mixture was diluted with ethyl acetate (20 mL) and washed with water (20 mL×3) then brine (20 mL). The organic layer was washed with brine, dried over sodium sulfate, filtered, and concentrated under reduced pressure. The residue was purified by silica gel column chromatography (9% EtOAc in hexanes) to give lactone **35** (87 mg, 100%) as white solid.

Compound 35.

^{1}H NMR (400 MHz, CDCl$_3$) δ 7.55–7.49 (m, 2H), 7.39–7.31 (m, 3H), 5.75 (dddd, J = 16.3, 10.1, 8.2, 5.5 Hz, 1H), 4.98 (dd, J = 17.1, 2.0 Hz, 1H), 4.90 (d, J = 10.6, 1.4 Hz, 1H), 2.57 (d, J = 17.0 Hz, 1H), 2.49 (d, J = 16.9 Hz, 1H), 2.38–2.25 (m, 2H), 2.11–2.00 (m, 1H), 1.85–1.74 (m, 1H), 1.70–1.56 (m, 2H), 1.45–1.35 (m, 1H), 1.07 (s, 3H), 1.04 (s, 3H), 0.34 (s, 3H), 0.32 (s, 3H), 0.09 (s, 9H).

^{13}C NMR (101 MHz, CDCl$_3$) δ 175.1, 140.2, 137.6, 134.8, 129.2, 127.8, 114.9, 101.7, 81.3,

46.0, 44.2, 35.0, 34.1, 32.0, 30.6, 22.2, 18.0, 2.3, –4.4, –4.6.

IR (film): 2956, 1775, 1428, 1251, 1108, 1004, 839, 810, 768, 736, 702 cm^{-1}.

HRMS (ESI): *m/z* Calc. for $C_{24}H_{39}O_3Si_2$ $[M+H]^+$: 431.2432, found: 431.2430.

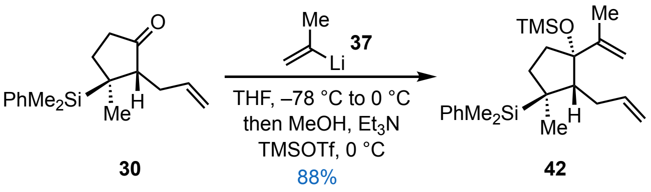

One-pot synthesis of 42 from 30.

Under argon, *tert*-butyllithium (5.0 equiv., 1.7 M in pentane, 1.35 mL, 2.5 mmol) was added to a solution of 2-bromopropene (2.5 equiv., 0.11 mL, 1.2 mmol) in THF (10 mL) at –78 °C. After stirring at –78 °C for 30 minutes, a solution of **30** (136 mg, 0.5 mmol) in THF (5 mL) was added. The reaction mixture was allowed to warm up to 0 °C over 3 hours, then stirred at 0 °C for 3 hours. Methanol (2.5 equiv., 50 µL, 1.2 mmol) was added, followed by the addition of triethylamine (15 equiv., 1.05 mL, 7.5 mmol) and trimethylsilyl trifluoromethanesulfonate (10 equiv., 0.91 mL, 5.0 mmol) at 0 °C. After stirring for 30 minutes at 0 °C, methanol (10 equiv., 0.20 mL, 4.8 mmol) then $NaHCO_3$ solution (20 mL) was added to the reaction mixture, and the desired organic material was extracted with ethyl acetate (20 mL×3). The combined organic layer was washed with brine, dried over sodium sulfate, filtered, and concentrated under reduced pressure. The residue was purified by flash silica gel column chromatography (hexanes) to give silyl ether **42** (170 mg, 88%) as a colorless liquid.

Compound 42.

^{1}H NMR (400 MHz, $CDCl_3$) δ 7.55–7.50 (m, 2H), 7.38–7.30 (m, 3H), 5.74 (dddd, *J* = 17.3, 10.2, 8.6, 5.7 Hz, 1H), 4.94–4.90 (m, 1H), 4.87–4.73 (m, 3H), 2.37–2.26 (m, 1H), 1.95–1.73 (m, 3H), 1.68–1.62 (m, 1H), 1.58–1.50 (m, 2H), 1.44 (br s, 3H), 1.07 (s, 3H), 0.31 (s, 3H), 0.30 (s, 3H), 0.10 (s, 9H).

^{13}C NMR (101 MHz, $CDCl_3$) δ 149.2, 140.6, 138.1, 134.8, 129.1, 127.7, 113.9, 111.6, 89.4, 51.3, 37.4, 36.3, 30.6, 29.3, 20.1, 19.7, 2.7, –4.9, –5.2.

IR (film): 3070, 2954, 2869, 1638, 1427, 1250, 1111, 836, 809, 768, 735, 701 cm^{-1}.

HRMS (APCI): *m/z* Calc. for $C_{23}H_{39}OSi_2$ $[M+H]^+$: 387.2534, found: 387.2529;

TLC (9% EtOAc in hexanes): $R_f = 0.78$.

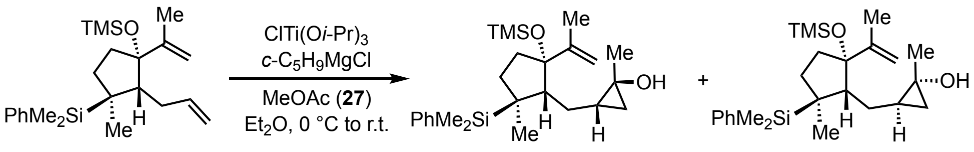

42 **43a** (major) **43b** (minor)

Kulinkovich reaction between 42 and methyl acetate.

Under argon, ClTi(O*i*-Pr)$_3$ (2.5 equiv., 1.20 mL, 5.0 mmol) was added to a flame-dried flask charged with **42** (1 equiv., 760 mg, 2.0 mmol), methyl acetate (2.5 equiv., 0.40 mL, 5.0 mmol) and anhydrous diethyl ether (40 mL). Then cyclopentylmagnesium chloride (5.0 equiv., 2.0 M in diethyl ether, 5.0 mL, 10 mmol) was added via syringe pump over 75 minutes at 0 °C. After completion of addition, the reaction mixture was stirred at 23 °C for 1 hour before addition of saturated NH_4Cl aqueous solution (1.6 mL). The reaction mixture was stirred until it became a grey suspension, then it was filtered over Celite (washed by EtOAc) and concentrated under reduced pressure. The residue was purified by flash silica gel column chromatography (5~10% EtOAc in hexanes) to give a mixture of cyclopropanols **43a** and **43b** (*dr* = 2.0:1, 701 mg, 83%) as colorless oil, together with recovered **42** (101 mg, 13% recovery) as colorless oil. This mixture of cyclopropanols was directly used in the next step.

Analytical samples of **43a** and **43b** were prepared through preparative TLC (20% EtOAc in hexanes, contains 0.1% Et_3N).

Compound 43a:

^{1}H NMR (400 MHz, CDCl$_3$) δ 7.56–7.51 (m, 2H), 7.37–7.30 (m, 3H), 4.97–4.93 (m, 1H), 4.79–4.74 (m, 1H), 1.96–1.84 (m, 2H), 1.76–1.59 (m, 4H), 1.52 (br s, 3H), 1.20 (s, 3H), 1.13–1.02 (m, 1H), 1.07 (s, 3H), 0.70–0.60 (m, 2H), 0.31 (s, 3H), 0.29 (s, 3H), 0.12 (s, 9H), -0.14 (dd, J = 6.6, 5.0 Hz, 1H).

^{13}C NMR (101 MHz, CDCl$_3$) δ 150.0, 138.2, 134.8, 129.2, 127.8, 111.1, 89.8, 56.5, 50.2, 37.8, 36.4, 29.3, 25.7, 25.0, 20.4, 20.3, 20.1, 19.7, 2.9, −4.8, −5.2.

HRMS (ESI): *m/z* Calc. for $C_{25}H_{42}O_2NaSi_2$ $[M+Na]^+$: 453.2616, found: 453.2614;

TLC (20% EtOAc in hexanes): $R_f = 0.65$.

Compound 43b:

^{1}H NMR (400 MHz, CDCl$_3$) δ 7.56–7.51 (m, 2H), 7.37–7.30 (m, 3H), 4.89–4.85 (m, 1H), 4.77–4.73 (m, 1H), 1.94–1.85 (m, 2H), 1.80–1.64 (m, 2H), 1.59–1.49 (m, 1H), 1.51 (br s, 3H), 1.42 (dd, *J* = 14.1, 8.3 Hz, 1H), 1.32 (s, 3H), 1.12–1.03 (m, 1H), 1.07 (s, 3H), 0.96 (dq, *J* = 9.5, 6.9 Hz, 1H), 0.68 (dd, *J* = 10.2, 5.1 Hz, 1H), 0.33 (s, 3H), 0.31 (s, 3H), 0.11 (s, 9H), -0.12 (dd, *J* = 6.5, 5.0 Hz, 1H).

^{13}C NMR (101 MHz, CDCl$_3$) δ 150.9, 138.2, 134.8, 129.1, 127.7, 110.3, 89.9, 56.1, 51.3, 38.4, 36.9, 30.2, 26.8, 25.1, 21.8, 21.1, 19.9, 18.9, 2.8, –4.9, –4.9.

HRMS (ESI): *m/z* Calc. for $C_{25}H_{42}O_2NaSi_2$ [M+Na]$^+$: 453.2616, found: 453.2617.

TLC (20% EtOAc in hexanes): R_f = 0.62.

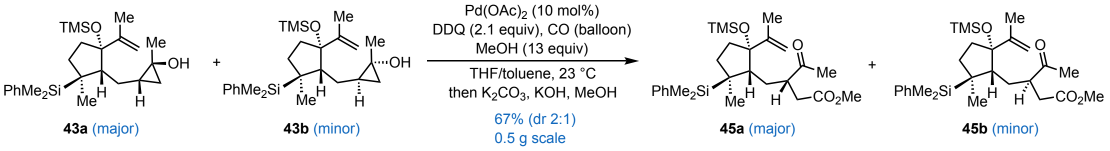

Catalytic ring-opening carbonylation of 43.

Under a balloon pressure of CO, a solution of **43** (1 equiv., 480 mg, 1.1 mmol, **43a**:**43b** = 2.0:1) in toluene (12 mL) was rapidly added to an oven-dried 100 mL flask charged with palladium(II) acetate (11 mol%, 27.5 mg, 0.12 mmol), 2,3-dichloro-5,6-dicyano-1,4-benzoquinone (2.1 equiv., 528 mg, 2.3 mmol), anhydrous methanol (13 equiv., 0.60 mL, 15 mmol) and THF (12 mL). The reaction mixture was stirred vigorously at 23 °C overnight. After full consumption of cyclopropanols, the reaction mixture was exposed to open air and diluted with anhydrous methanol (24 mL). Potassium carbonate (8.2 equiv., 1.25 g, 9.0 mmol) and potassium hydroxide (10 equiv., 0.61 g, 11 mmol) were added sequentially. After the reaction was complete (usually less than 10 minutes using TLC analysis), water (100 mL) was added, and the desired organic material was extracted with diethyl ether (75 mL×4). The combined organic layer was washed with brine, NH_4Cl solution and brine sequentially, dried over sodium sulfate, filtered, and concentrated under reduced pressure. The residue was purified by silica gel column chromatography (5% to 10% EtOAc in hexanes) to give ketoester **45a** (245 mg, 45%) and ketoester **45b** (121 mg, 22%) both as colorless oil.

Note: Adding external inorganic bases (K_3PO_4, K_2CO_3 or NaOAc) at the beginning of the carbonylation reaction led to the formation of the corresponding cyclopropyl methyl carbonates

only, without any ring-opening product detected.

Compound 45a:

^{1}H NMR (600 MHz, CDCl$_3$) δ 7.51 (dd, *J* = 6.7, 2.3 Hz, 2H), 7.38–7.33 (m, 3H), 5.00 (br s, 1H), 4.72 (br s, 1H), 3.62 (s, 3H), 3.32–3.26 (m, 1H), 2.50 (dd, *J* = 16.8, 11.2 Hz, 1H), 2.19 (s, 3H), 2.13 (dd, *J* = 16.9, 3.1 Hz, 1H), 2.09–2.03 (m, 1H), 1.91–1.76 (m, 4H), 1.56–1.49 (m, 1H), 1.40 (s, 3H), 1.01 (s, 3H), 0.90–0.82 (m, 1H), 0.31 (s, 6H), 0.17 (s, 9H).

^{13}C NMR (101 MHz, CDCl$_3$) δ 212.0, 173.3, 150.8, 137.2, 134.6, 129.4, 127.9, 110.6, 89.1, 51.7, 46.6, 45.2, 39.2, 36.3, 33.9, 31.3, 29.1, 28.6, 19.3, 17.7, 2.8, –5.0, –5.1.

IR (film): 2953, 2869, 1738, 1715, 1251, 1165, 837, 810, 769, 737, 703 cm^{-1}.

HRMS (ESI): *m/z* Calc. for $C_{27}H_{44}O_4NaSi_2$ $[M+Na]^+$: 511.2670, found: 511.2673.

TLC (20% EtOAc in hexanes): R_f = 0.53.

Compound 45b:

^{1}H NMR (600 MHz, CDCl$_3$) δ 7.54–7.50 (m, 2H), 7.36–7.30 (m, 3H), 4.96 (d, *J* = 2.0 Hz, 1H), 4.76 (t, *J* = 1.7 Hz, 1H), 3.63 (s, 3H), 3.14 (tt, *J* = 9.4, 4.7 Hz, 1H), 2.54 (dd, *J* = 16.4, 9.9 Hz, 1H), 2.21 (dd, *J* = 16.4, 4.5 Hz, 1H), 2.09 (s, 3H), 1.84–1.71 (m, 4H), 1.54 (dd, *J* = 9.4, 4.9 Hz, 1H), 1.52–1.46 (m, 2H), 1.35 (brs, 3H), 1.03 (s, 3H), 0.34 (s, 3H), 0.32 (s, 3H), 0.11 (s, 9H).

^{13}C NMR (151 MHz, CDCl$_3$) δ 213.2, 172.8, 150.9, 137.6, 134.9, 129.2, 127.8, 110.9, 89.1, 51.8, 48.0, 44.9, 38.7, 38.0, 36.5, 31.2, 31.1, 29.7, 19.5, 17.9, 2.6, –5.1, –5.1.

IR (film): 2953, 2868, 1739, 1711, 1250, 1111, 861, 837, 811, 770, 737, 703 cm^{-1}.

HRMS (ESI): *m/z* Calc. for $C_{27}H_{45}O_4NaSi_2$ $[M+H]^+$: 489.2851, found: 489.2857.

TLC (20% EtOAc in hexanes): R_f = 0.56.

Note: Two conditions were tried to epimerize **45b** into **45a** (1:20 DBU/toluene, 110 °C or 1:10 *i*-Pr_2NH/MeOH, 60 °C), but no reaction occurred.

Isolation of the acetal-lactone intermediate 44.

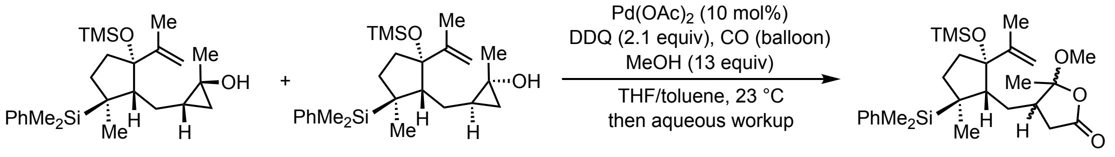

43a (major) **43b** (minor) **44**

Note: Without addition of bases and more methanol, the intermediate acetal-lactone **44** was

isolated after aqueous workup and column purification as a mixture of all four diastereomers (*dr* = 1:0.6:0.24:0.15 based on ^{1}H-NMR). The major diastereomer of **44**, namely **44a**, was obtained through preparative TLC (10% EtOAc in hexanes). The other three diastereomers of **44** formed an inseparable mixture. **44a** can be cleanly transformed into **45a** by treatment with potassium carbonate in methanol at ambient temperature in 10 minutes.

Compound 44a.

^{1}H NMR (600 MHz, $CDCl_3$) δ 7.53–7.48 (m, 2H), 7.38–7.32 (m, 3H), 4.87–4.84 (m, 1H), 4.66–4.63 (m, 1H), 3.29 (s, 3H), 2.65–2.57 (m, 1H), 2.24 (dd, *J* = 16.7, 8.2 Hz, 1H), 2.18 (dd, *J* = 16.7, 12.0 Hz, 1H), 1.89 (ddd, *J* = 13.4, 10.0, 7.5 Hz, 1H), 1.85–1.79 (m, 2H), 1.73 (ddd, *J* = 12.7, 10.0, 7.8 Hz, 1H), 1.65 (dd, *J* = 12.1, 2.3 Hz, 1H), 1.51 (ddd, *J* = 12.7, 7.5, 3.9 Hz, 1H), 1.47 (s, 3H), 1.37 (d, *J* = 1.2 Hz, 3H), 1.22 (ddd, *J* = 13.6, 11.9, 2.2 Hz, 1H), 1.03 (s, 3H), 0.32 (s, 3H), 0.31 (s, 3H), 0.12 (s, 9H).

^{13}C NMR (151 MHz, $CDCl_3$) δ 177.1, 151.5, 137.3, 134.6, 129.3, 127.9, 110.3, 109.9, 88.7, 50.3, 47.8, 43.9, 39.5, 36.3, 34.3, 31.8, 25.8, 19.5, 19.3, 17.3, 2.6, –4.9, –5.2.

IR (film): 2954, 2869, 2838, 1786, 1251, 1062, 915, 838, 768, 703 cm^{-1}.

HRMS (APCI): *m/z* Calc. for $C_{27}H_{45}O_4Si_2$ $[M+H]^+$: 489.2851, found: 489.2861.

$C_{26}H_{41}O_3Si_2$ $[M-OMe]^+$: 457.2589, found: 457.2595.

TLC (20% EtOAc in hexanes): R_f = 0.59.

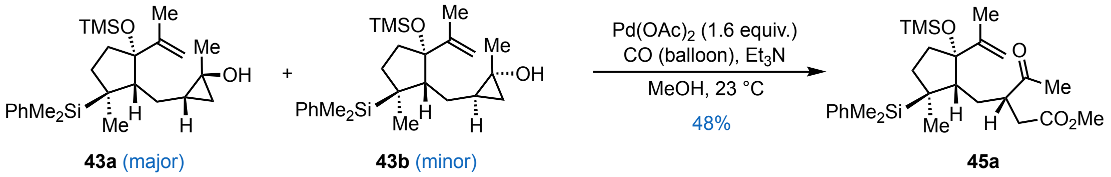

Stoichiometric version of ring-opening carbonylation of 43.

Under a balloon pressure of CO, a solution of **43** (1.0 equiv., 300 mg, 0.70 mmol, **43a**:**43b** = 2.0:1) in anhydrous methanol (5.0 mL) was rapidly added to a flame-dried 25 mL microwave tube charged with palladium(II) acetate (1.6 equiv., 250 mg, 1.1 mmol), triethyl amine (3.3 equiv., 0.32 mL, 2.3 mmol) and methanol (2.5 mL). The reaction mixture was stirred vigorously at 23 °C overnight. NH_4Cl solution was added to the reaction mixture and the desired organic material was extracted with ethyl acetate (50 mL×3). The combined organic layer was washed

with brine, dried over sodium sulfate, filtered, and concentrated under reduced pressure. The residue was purified by silica gel column chromatography (6% EtOAc in hexanes) to give impure ketoester **45a** (234 mg, ~70% NMR purity, 48% calculated yield) as colorless oil. This impure material went through the next ozonolysis to afford dione **46a** (151 mg, 44% yield from **43** over 2 steps).

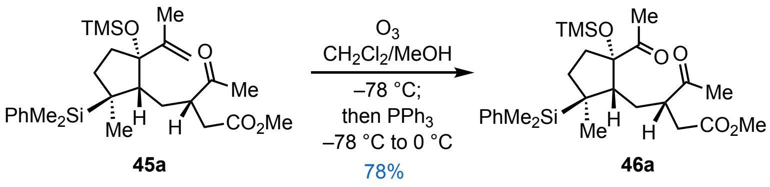

Ozonolysis of 45a.

At –78 °C, ozone was bubbled through a solution of **45a** (1.0 equiv., 245 mg, 0.50 mmol) in DCM/MeOH (1:1, 24 mL) until the solution became deep blue and lasted for 2 minutes. It was purged with O_2 until it became colorless, then a solution of PPh_3 (2.7 equiv., 360 mg, 1.4 mmol) in DCM (~3 mL) was added at –78 °C. The reaction mixture was stirred at –78 °C for 90 minutes and then at 23 °C for 30 minutes, before it was concentrated under reduced pressure. The residue was purified by silica gel column chromatography (5-10% EtOAc in hexanes) to give pure dione **46a** (170 mg, 69%) as colorless oil. Impure fractions were combined and purified by another column chromatography (10% EtOAc in hexanes) to give extra **46a** (21 mg, 9%; total 78%).

Compound 46a:

^{1}H NMR (600 MHz, CDCl$_3$) δ 7.55–7.51 (m, 2H), 7.41–7.35 (m, 3H), 3.62 (s, 3H), 2.62 (tt, *J* = 11.5, 2.5 Hz, 1H), 2.52 (dd, *J* = 16.7, 11.0 Hz, 1H), 2.19 (s, 3H), 2.11–2.02 (m, 2H), 1.99–1.93 (m, 2H), 1.88–1.77 (m, 2H), 1.82 (s, 3H), 1.63 (ddd, *J* = 12.9, 7.4, 5.6 Hz, 1H), 1.03 (s, 3H), 0.87 (ddd, *J* = 14.0, 11.8, 2.5 Hz, 1H), 0.34 (s, 3H), 0.33 (s, 3H), 0.15 (s, 9H).

^{13}C NMR (151 MHz, CDCl$_3$) δ 211.3, 210.8, 172.7, 136.8, 134.6, 129.7, 128.1, 92.2, 51.9, 48.9, 45.6, 39.3, 36.8, 33.8, 31.8, 29.2, 27.8, 24.6, 17.8, 2.6, –5.1, –5.2.

IR (film): 2953, 1737, 1714, 1249, 1141, 840, 809, 769, 737, 703 cm^{-1}.

HRMS (ESI): *m/z* Calc. for $C_{26}H_{42}O_5NaSi_2$ [M+Na]$^+$: 513.2463, found: 513.2469.

TLC (20% EtOAc in hexanes): R_f = 0.40.

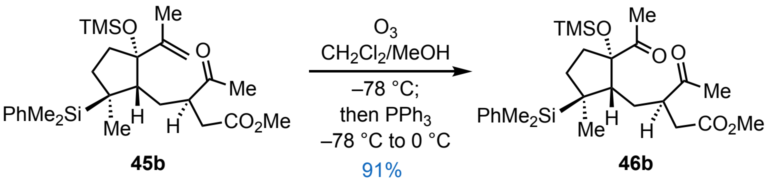

Ozonolysis of 45b.

At –78 °C, ozone was bubbled through a solution of **45b** (1.0 equiv., 64.2 mg, 0.13 mmol) in DCM/MeOH (1:1, 6.4 mL) until the solution became deep blue and lasted for 2 minutes. It was purged with O_2 until it became colorless, then a solution of PPh_3 (2.7 equiv., 93 mg, 0.36 mmol) in DCM (~1 mL) was added at –78 °C. The reaction mixture was stirred at –78 °C for 1 hour and then at 23 °C for 1 hour, before it was concentrated under reduced pressure. The residue was purified by silica gel column chromatography (4% to 6% EtOAc in hexanes) to give dione **46b** (58.8 mg, 91%) as colorless oil.

Compound 46b:

^{1}H NMR (600 MHz, $CDCl_3$) δ 7.57 (dd, *J* = 6.4, 3.0 Hz, 2H), 7.37–7.34 (m, 3H), 3.63 (s, 3H), 2.70 (dddd, *J* = 10.3, 8.5, 4.9, 3.2 Hz, 1H), 2.53 (dd, *J* = 15.9, 8.8 Hz, 1H), 2.34 (dd, *J* = 15.9, 5.0 Hz, 1H), 2.12 (dd, *J* = 11.3, 3.2 Hz, 1H), 2.06 (s, 3H), 1.88–1.78 (m, 3H), 1.77 (s, 3H), 1.60–1.52 (m, 2H), 1.48 (ddd, *J* = 13.7, 10.6, 3.3 Hz, 1H), 1.01 (s, 3H), 0.37 (s, 3H), 0.36 (s, 3H), 0.09 (s, 9H).

^{13}C NMR (150 MHz, $CDCl_3$) δ 211.8, 211.6, 172.1, 137.1, 134.9, 129.3, 127.8, 92.0, 51.9, 49.2, 45.7, 39.4, 38.6, 36.8, 32.5, 29.9, 29.3, 24.6, 17.2, 2.3, –5.2, –5.3.

IR (film): 2952, 1740, 1713, 1249, 1145, 839, 810, 770, 738, 703 cm^{-1}.

HRMS (ESI): *m/z* Calc. for $C_{26}H_{43}O_5Si_2$ $[M+H]^+$: 491.2644, found: 491.2650.

TLC (20% EtOAc in hexanes): R_f = 0.45.

Table S1. Effect of bases on aldol cyclization of 46a.

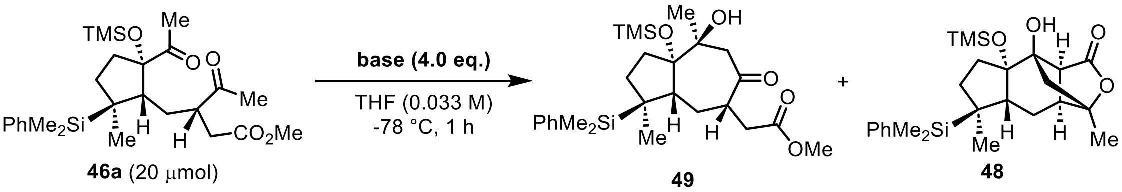

Entry	Base	Yield of **49**[a]	Yield of **48**[a]	Ratio (**49**:**48**)
1	LiHMDS (1.0 M in THF)[b]	8%	69%	1:8.6
2	NaHMDS (1.0 M in THF)[b]	19%	43%	1:2.3
3[c]	KHMDS (1.0 M in THF)[b]	22%	n.d.	n.d.
4	LDA (2.0 M in THF/EtPh/heptanes)[b]	54%	23%	2.3:1
5	LiTMP (0.5 M in THF/hexanes)[d]	63%	13%	4.8:1
6[e]	LiNCy2 (0.4 M in THF/hexanes)[d]	6%	n.d.	n.d.
7	LiCyan (0.2 M in THF/hexanes)[d]	6%	52%	1:8.7

[a] NMR yield after aqueous workup (using 5.0 μl $BrCH_2CH_2Br$ as internal standard). No starting material has been detected in all entries except entry 6. [b] Purchased commercially and used without further purification. [c] A complex mixture was formed. [d] Freshly prepared from the corresponding secondary amine and *n*-butyllithium (2.5 M in hexanes) in THF. 2,2,6,6-Tetramethylpiperidine and dicyclohexylamine were purchased commercially and used without further purification. CyanH was prepared according to the reported protocol.[2] [e] <67% conversion based on NMR.

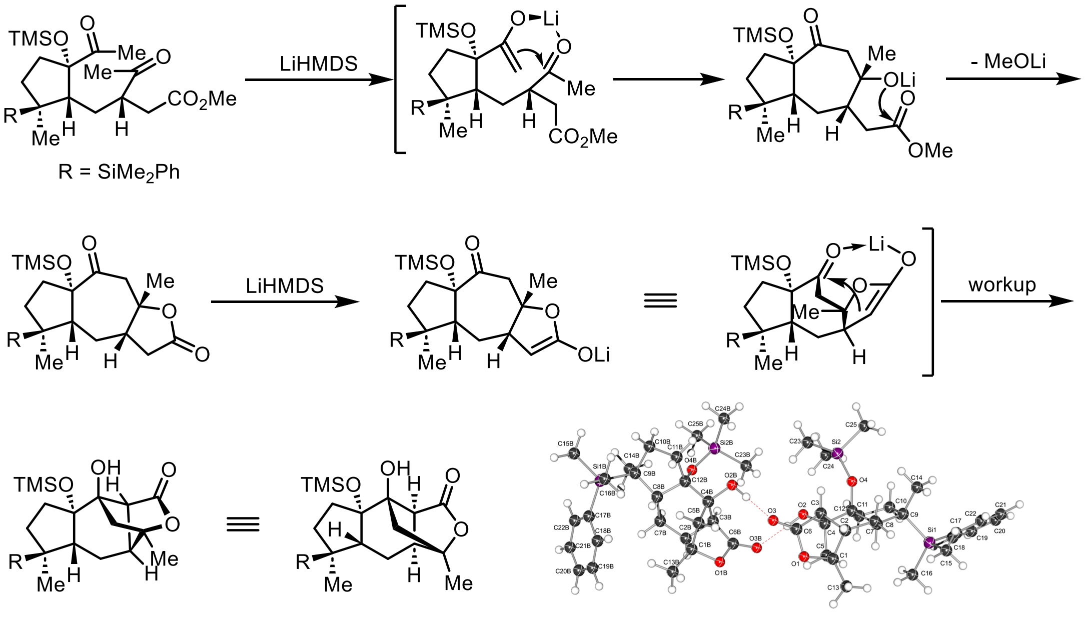

Figure S1. Proposed mechanism for the unexpected formation of 48.

Discussion

Acidity of α-H of methyl ketones:

49 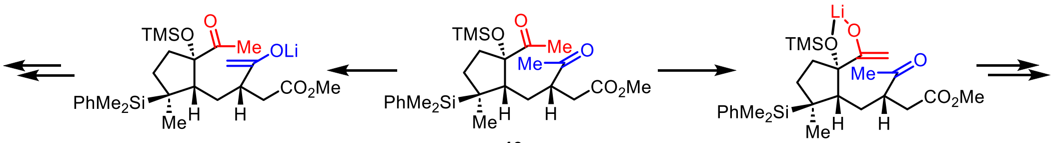 48

46a

The α-methyl group shown in red should be more sterically hindered and more acidic than the one shown in blue due to adjacent quaternary carbon center with a TMS ether. The corresponding **'red enolate'** may be further stabilized by the weak coordinating and electron-withdrawing -OTMS group. Therefore, in order to form the blue enolate, a bulky and stronger base is needed.

Basicity [3] of lithium amides:

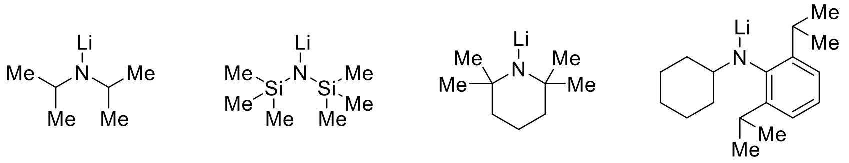

	LDA	LiHMDS	LiTMP	LiCyan
pK_a of amine in THF	35.7	29.5	37.3	not known

Although pK_a of CyanH is not available, pK_a of a secondary amine with an aryl and an alkyl substituent can be predicted to be lower than pK_a of di-alkyl amines.

Thus, we propose that a stronger and bulky base (LDA or LiTMP) leads to the formation of **49**, while a weaker base (LiHMDS or LiCyan) leads to the formation of **48**.

Table S2. Aldol cyclization of 46b.

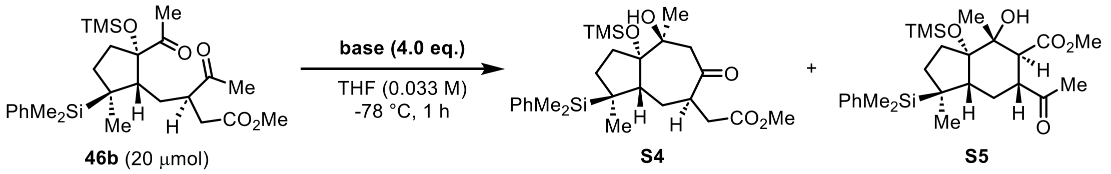

Entry	Base	Yield of **S4**[a]	Yield of **S5**[a]
1	LiHMDS (1.0 M in THF)[b]	18%	17%
2	NaHMDS (1.0 M in THF)[b]	26%	n.d.
3	KHMDS (1.0 M in THF)[b]	21% (20%[c])	n.d.
4	LDA (2.0 M in THF/EtPh/heptanes)[b]	20%	35%

[a] NMR yield after aqueous workup (using 5.0 μl $BrCH_2CH_2Br$ as internal standard). No starting material has been detected in all entries. [b] Purchased commercially and used without further purification. [c] Isolated yield (Procedure: 0.32 mL KHMDS was added to a solution of 40 mg **46b** in 2.0 mL THF at −78 °C. Quenched after stirring at −78 °C for 30 min. 7.8 mg of **S4** was isolated after aqueous workup and column chromatography).

The aldol cyclization of **46b** usually afforded a complex mixture, from which **S4** and **S5** were isolated after column chromatography (9% to 20% EtOAc in hexanes) and preparative TLC (20% EtOAc in hexanes). The formation of byproduct **S5** should involve an ester enolate intermediate. Stereochemistry of **S4** and **S5** was assigned based on NOESY and coupling constants (**Figure S2**).

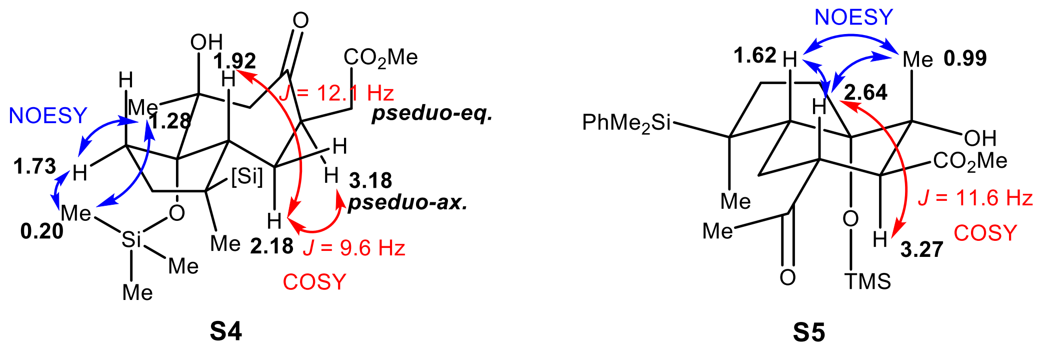

Figure S2. Structures of S4 and S5 indicated by COSY and NOESY.

Compound S4.

^{1}H NMR (800 MHz, CDCl$_3$) δ 7.47 (dd, *J* = 7.2, 2.1 Hz, 2H), 7.39–7.32 (m, 3H), 3.65 (s, 3H), 3.25 (d, *J* = 11.4 Hz, 1H), 3.18 (dp, *J* = 9.3, 4.5 Hz, 1H), 2.61 (dd, *J* = 16.9, 9.3 Hz, 1H), 2.16 (td, *J* = 12.8, 9.6 Hz, 1H), 2.11 (d, *J* = 11.4 Hz, 1H), 1.99 (br s, 1H), 1.95–1.89 (m, 2H), 1.86 (ddd, *J* = 12.3, 8.2, 3.9 Hz, 1H), 1.81 (dt, *J* = 13.4, 9.0 Hz, 1H), 1.73 (ddd, *J* = 13.5, 7.2, 3.9 Hz, 1H), 1.42 (ddd, *J* = 12.1, 9.9, 7.2 Hz, 1H), 1.28 (s, 3H), 0.99 (s, 3H), 0.78 (dt, *J* = 14.0, 3.4 Hz, 1H), 0.26 (s, 3H), 0.24 (s, 3H), 0.19 (s, 9H).

^{13}C NMR (151 MHz, CDCl$_3$) δ 212.4, 173.4, 137.4, 134.7, 129.3, 127.9, 91.9, 74.4, 52.9, 51.8, 46.0, 44.0, 36.2, 35.2, 33.1, 29.6, 27.3, 25.0, 19.6, 3.2, –5.4, –5.7.

IR (film): 3456, 2953, 2868, 1737, 1697, 1251, 1134, 1112, 835, 810, 770, 731, 702 cm^{-1}.

HRMS (APCI): *m/z* Calc. for $C_{26}H_{43}O_5Si_2$ $[M+H]^+$: 491.2644, found: 491.2648.

TLC (20% EtOAc in hexanes): R$_f$ = 0.30.

Compound S5.

^{1}H NMR (800 MHz, CDCl$_3$) δ 7.55–7.52 (m, 2H), 7.40–7.35 (m, 3H), 3.69 (s, 3H), 3.40 (br s, 1H), 3.27 (d, *J* = 11.6 Hz, 1H), 2.64 (td, *J* = 12.2, 4.0 Hz, 1H), 2.01 (dd, *J* = 12.7, 8.0 Hz, 1H), 1.99 (s, 3H), 1.84 (dd, *J* = 12.8, 6.8 Hz, 1H), 1.62 (dd, *J* = 12.6, 3.4 Hz, 1H), 1.53 (td, *J* = 12.7, 6.9 Hz, 1H), 1.39 (q, *J* = 12.5 Hz, 1H), 1.13 (td, *J* = 12.7, 8.0 Hz, 1H), 0.99 (s, 3H), 0.98 (s, 3H), 0.88 (dt, *J* = 12.0, 3.7 Hz, 1H), 0.30 (s, 6H), 0.15 (s, 9H).

^{13}C NMR (101 MHz, CDCl$_3$) δ 209.8, 175.0, 137.8, 134.3, 129.3, 127.8, 88.2, 75.6, 52.3, 50.8, 50.2, 47.7, 35.3, 32.1, 28.7, 25.9, 25.2, 21.5, 19.9, 3.2, –6.0, –6.3.

IR (film): 2951, 2867, 1735, 1710, 1248, 1222, 1109, 838, 807, 769, 736, 701 cm^{-1}.

HRMS (APCI): *m/z* Calc. for $C_{26}H_{43}O_5Si_2$ $[M-H]^-$: 489.2498, found: 489.2500.

TLC (20% EtOAc in hexanes): R$_f$ = 0.40.

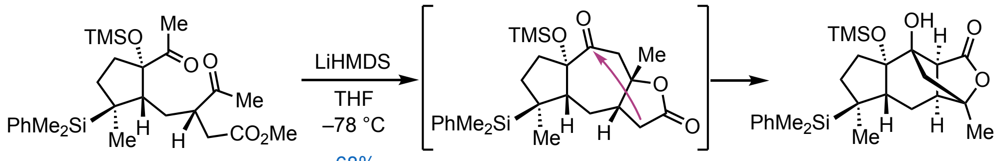

46a **47** **48**

Aldol cyclization of 46a using LiHMDS as base.

At −78 °C, to a solution of **46a** (1.0 equiv., 30.0 mg, 61 μmol) in THF (1.0 mL) was added

LiHMDS (4.0 equiv., 0.24 mL, 1.0 M in THF, 0.24 mmol). The reaction mixture was stirred at –78 °C for 1 hour. Saturated NH_4Cl solution was added to the reaction mixture and the desired organic material was extracted with ethyl acetate (10 mL×3). The combined organic layer was washed with brine, dried over sodium sulfate, filtered, and concentrated under reduced pressure. The residue was purified by silica gel column chromatography (20% EtOAc in hexanes) to give lactone **48** (19.0 mg, 68%) as white needles.

Compound 48.

^{1}H NMR (400 MHz, $CDCl_3$) δ 7.51 (dd, J = 7.6, 1.7 Hz, 2H), 7.42–7.32 (m, 3H), 2.99 (s, 1H), 2.34–2.30 (m, 1H), 2.14 (dd, J = 13.3, 8.6 Hz, 1H), 1.81 (dd, J = 12.8, 7.2 Hz, 1H), 1.79 (br s, 1H), 1.70–1.49 (m, 5H), 1.41 (s, 3H), 1.20 (dd, J = 13.3, 4.1 Hz, 1H), 1.13 (s, 3H), 0.98–0.86 (m, 1H), 0.32 (s, 3H), 0.30 (s, 3H), 0.19 (s, 9H).

^{13}C NMR (101 MHz, $CDCl_3$) δ 176.6, 137.6, 134.3, 129.6, 128.0, 92.1, 89.1, 83.0, 55.3, 54.3, 47.2, 45.4, 34.7, 31.9, 25.9, 20.5, 19.2, 17.2, 3.0, –5.0, –5.4.

IR (film): 3448, 2953, 2931, 2866, 1759, 1388, 1260, 1111, 842, 826, 809, 769, 737, 703 cm^{-1}.

HRMS (APCI): *m/z* Calc. for $C_{25}H_{37}O_4Si_2$ $[M-H]^-$: 457.2236, found: 457.2235.

TLC (33% EtOAc in hexanes): R_f = 0.63.

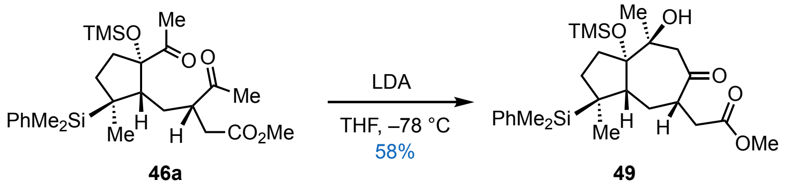

Aldol cyclization of 46a using LDA as base.

At –78 °C, to a solution of **46a** (1.0 equiv., 170 mg, 0.35 mmol) in THF (7.0 mL) was added LDA (4.0 equiv., 0.70 mL, 2.0 M in THF/heptane/ethylbenzene, 1.4 mmol). The reaction mixture was stirred at –78 °C for 1 hour. NH_4Cl solution was added to the reaction mixture and the desired organic material was extracted with ethyl acetate (20 mL×3). The combined organic layer was washed with brine, dried over sodium sulfate, filtered, and concentrated under reduced pressure. The residue was purified by silica gel column chromatography (10-20% EtOAc in hexanes) to give ketone **49** (98 mg, 58%) as white solid.

Compound 49:

^{1}H NMR (400 MHz, $CDCl_3$) δ 7.57–7.52 (m, 2H), 7.40–7.31 (m, 3H), 3.63 (s, 3H), 2.99 (d, *J* = 15.1 Hz, 1H), 2.90–2.80 (m, 1H), 2.55 (dd, *J* = 15.1, 2.3 Hz, 1H), 2.48 (dd, *J* = 16.4, 8.1 Hz, 1H), 2.37 (d, *J* = 15.2 Hz, 1H), 2.16 (dd, *J* = 16.4, 5.6 Hz, 1H), 2.01–1.93 (m, 1H), 1.72–1.65 (m, 1H), 1.58–1.31 (m, 3H), 1.27 (s, 3H), 0.96 (s, 3H), 0.78 (dd, *J* = 13.7, 2.8 Hz, 1H), 0.30 (s, 3H), 0.29 (s, 3H), 0.13 (s, 9H).

^{13}C NMR (101 MHz, $CDCl_3$) δ 212.2, 173.0, 137.7, 134.5, 129.3, 127.8, 91.3, 74.1, 54.7, 51.7, 47.4, 46.5, 37.4, 34.6, 33.4, 28.6, 27.7, 27.3, 20.4, 3.0, −6.0, −6.2.

IR (film): 3489, 2952, 2867, 1738, 1695, 1251, 1112, 837, 810, 770, 737,702 cm^{-1}.

HRMS (ESI): *m/z* Calc. for $C_{26}H_{43}O_5Si_2$ $[M+H]^+$: 491.2644, found: 491.2648.

TLC (33% EtOAc in hexanes): R_f = 0.53.

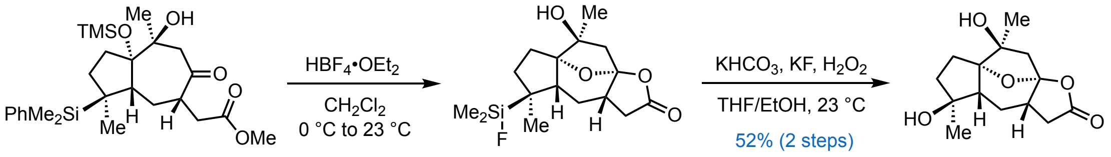

Flemming-Tamao oxidation of 49.

To a solution of **49** (1.0 equiv., 36.0 mg, 73 μmol) in CH_2Cl_2 (1.6 mL) was added tetrafluoroboric acid diethyl ether complex (8.0 equiv., 0.08 mL, 0.58 mmol) at 0 °C. The reaction mixture was stirred at 0 °C for 30 min then at 23°C for 75 min. After the reaction was complete, the reaction mixture was added to cold $NaHCO_3$ solution (20 mL) and extracted with dichloromethane (10 mL×3). The combined organic layer was washed with brine, dried over sodium sulfate, filtered, and concentrated under reduced pressure to afford crude **S6**, which was redissolved in THF/EtOH (1.8 mL/1.8 mL). Potassium bicarbonate (3.0 equiv., 21.6 mg, 0.22 mmol), potassium fluoride (3.0 equiv., 12.6 mg, 0.22 mmol) and hydrogen peroxide (10 equiv., 30% aqueous solution, 72 μL, 0.72 mmol) were added sequentially. The reaction mixture was stirred at 23°C overnight, then diluted with ethyl acetate. Saturated $NaHCO_3$ (5 mL) and $Na_2S_2O_3$ (5 mL) aqueous solution were added, and the desired organic material was extracted with ethyl acetate (10 mL×3). The combined organic layer was washed with brine, dried over sodium sulfate, filtered, and concentrated under reduced pressure. The residue was purified by flash silica gel column chromatography (3% MeOH in CH_2Cl_2) to give diol **50** (10.2 mg, 52%

over 2 steps) as off-white solid.

Compound S6 (crude):

^{1}H NMR (400 MHz, $CDCl_3$) δ 2.88–2.77 (m, 1H), 2.74 (dd, *J* = 13.4, 5.2 Hz, 1H), 2.44–2.30 (m, 3H), 2.07 (d, *J* = 13.8 Hz, 1H), 1.95 (dddd, *J* = 13.5, 9.8, 6.9, 3.5 Hz, 1H), 1.89–1.75 (m, 3H), 1.64 (dt, *J* = 13.8, 5.8 Hz, 1H), 1.41 (s, 3H), 1.39–1.30 (m, 1H), 0.94 (d, *J* = 2.2 Hz, 3H), 0.26 (d, *J* = 1.4 Hz, 3H), 0.24 (d, *J* = 1.6 Hz, 3H).

^{13}C NMR (101 MHz, $CDCl_3$) δ 175.4, 107.9, 95.2, 76.2, 50.7, 41.2, 39.0, 35.9, 34.3, 32.2 (d, *J* = 11.5 Hz), 31.3, 27.4, 27.3, 16.7, –3.7 (d, *J* = 14.4 Hz), –4.3 (d, *J* = 15.5 Hz).

^{19}F NMR (376 MHz, $CDCl_3$) δ –158.3.

TLC (50% EtOAc in hexanes): R_f = 0.55.

Compound 50:

^{1}H NMR (400 MHz, $CDCl_3$) δ 2.85–2.75 (m, 1H), 2.45 (d, *J* = 13.8 Hz, 1H), 2.42–2.26 (m, 3H), 2.14–2.04 (m, 2H), 2.03–1.96 (m, 1H), 1.88–1.77 (m, 2H), 1.67 (ddd, *J* = 12.3, 7.1, 2.0 Hz, 1H), 1.44 (s, 3H), 1.21 (s, 3H), 1.09 (ddd, *J* = 14.1, 12.2, 9.7 Hz, 1H).

^{13}C NMR (101 MHz, $CDCl_3$) δ 175.3, 107.9, 94.2, 81.2, 75.6, 50.6, 49.3, 42.0, 38.1, 35.5, 28.5, 28.5, 25.9, 23.4.

(Note: The solubility of **50** in $CDCl_3$ is about 5 mg/mL only.)

^{1}H NMR (600 MHz, MeOD) δ 2.82 (dd, *J* = 17.8, 9.3 Hz, 1H), 2.41 (dtd, *J* = 9.9, 8.7, 6.8 Hz, 1H), 2.38–2.32 (m, 2H), 2.30 (d, *J* = 13.7 Hz, 1H), 2.15 (td, *J* = 13.5, 6.9 Hz, 1H), 2.06 (dd, *J* = 13.8, 0.7 Hz, 1H), 1.98 (ddd, *J* = 12.2, 6.8, 5.2 Hz, 1H), 1.79 (td, *J* = 12.9, 7.0 Hz, 1H), 1.67 (dd, *J* = 13.8, 7.0 Hz, 1H), 1.62 (dddd, *J* = 12.7, 6.9, 1.8, 0.8 Hz, 1H), 1.36 (s, 3H), 1.23 (s, 3H), 1.07 (ddd, *J* = 14.0, 12.3, 10.1 Hz, 1H).

^{13}C NMR (151 MHz, MeOD) δ 177.7, 109.5, 96.0, 81.8, 75.8, 51.6, 49.4, 42.6, 38.6, 36.2, 29.9, 29.4, 25.9, 23.5.

IR (film): 3453, 3309, 2965, 2937, 1787, 1450, 1364, 1269, 1221, 1139, 1011, 923, 816 cm^{-1}.

HRMS (APCI): *m/z* Calc. for $C_{14}H_{19}O_5$ $[M-H]^-$: 267.1238, found: 267.1236.

TLC (10% MeOH in CH_2Cl_2): R_f = 0.34.

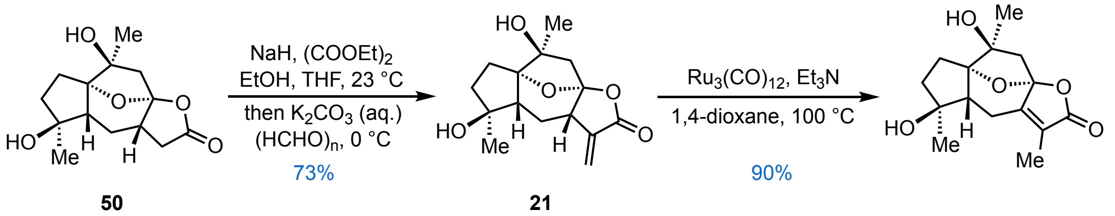

Synthesis of (±)-Phaeocaulisin A (3).

To a suspension of sodium hydride (60 wt% in mineral oil, 4.9 equiv., 4.6 mg, 0.12 mmol) in THF (0.25 mL) was added a solution of **50** (1.0 equiv., 6.3 mg, 23 μmol) and diethyl oxalate (7.8 equiv., 25.0 μL, 184 μmol) in THF (1.0 mL) at 0 °C. Then ethanol (15 equiv., 20 μL, 0.34 mmol) was added. The resulting suspension was stirred at 23 °C for 1 hour. Then potassium carbonate solution (20 wt% in H_2O, 0.25 mL) and paraformaldehyde (40 mg) were added at 0 °C, followed by stirring at 23 °C for 15 minutes. Brine (10 mL) was added, and the desired organic material was extracted with ethyl acetate (10 mL×3). The combined organic layer was dried over sodium sulfate, filtered, and concentrated under reduced pressure. The residue was purified by flash silica gel column chromatography (3% MeOH in CH_2Cl_2) to give unsaturated lactone **21** (4.8 mg, 73%), which was redissolved in dioxane (2.0 mL). To this solution, triruthenium dodecacarbonyl (1.1 equiv., 13.2 mg, 21 μmol) and triethyl amine (8.0 equiv., 20 μL, 0.14 mmol) were added. The reaction mixture was stirred at 100 °C for 75 minutes. After cooling to ambient temperature, the reaction mixture was diluted with ethyl acetate and concentrated under reduced pressure. The residue was purified by silica gel column chromatography (3% to 5% MeOH in CH_2Cl_2) to give (±)-phaeocaulisin A (**3**, 4.3 mg, 90%) as white solid.

Compound 21:

^{1}H NMR (400 MHz, MeOD) δ 6.24 (d, *J* = 3.2 Hz, 1H), 5.73 (d, *J* = 2.8 Hz, 1H), 2.91 (ddt, *J* = 10.4, 7.3, 3.0 Hz, 1H), 2.46 (ddd, *J* = 14.0, 5.2, 1.7 Hz, 1H), 2.31 (d, *J* = 13.7 Hz, 1H), 2.19–2.13 (m, 2H), 2.09 (d, *J* = 13.7 Hz, 1H), 1.73 (td, *J* = 13.0, 7.0 Hz, 1H), 1.66 (dd, *J* = 13.8, 7.0 Hz, 1H), 1.61 (ddd, *J* = 12.7, 6.5, 1.4 Hz, 1H), 1.38 (s, 3H), 1.26 (s, 3H), 1.09 (ddd, *J* = 14.0, 12.1, 10.5 Hz, 1H).

^{13}C NMR (201 MHz, MeOD) δ 170.6, 141.0, 123.8, 107.0, 96.1, 81.7, 76.2, 51.2, 48.7 (overlapped with solvent peak based on HSQC), 46.9, 38.6, 30.2, 27.8, 26.3, 23.6.

IR (film): 3350, 2952, 1766, 1651, 1115, 1015 cm^{-1}.

HRMS (APCI): *m/z* Calc. for $C_{15}H_{19}O_5$ $[M-H]^-$: 279.1238, found: 279.1236.

TLC (10% MeOH in CH_2Cl_2): R_f = 0.34 (same as **50**).

Phaeocaulisin A (3):

^{1}H NMR (600 MHz, MeOD) δ 2.93 (dd, *J* = 16.9, 0.9 Hz, 1H), 2.84 (ddq, *J* = 16.9, 9.0, 2.2 Hz, 1H), 2.62 (d, *J* = 8.8 Hz, 1H), 2.27 (d, *J* = 13.8 Hz, 1H), 2.16 (d, *J* = 13.8 Hz, 1H), 2.08 (ddd, *J* = 14.2, 11.2, 7.5 Hz, 1H), 1.88 (ddd, *J* = 13.5, 9.1, 7.5 Hz, 1H), 1.84–1.77 (m, 1H), 1.79 (d, *J* = 2.1 Hz, 3H), 1.64 (ddd, *J* = 13.9, 9.1, 4.6 Hz, 1H), 1.40 (s, 3H), 1.17 (s, 3H).

^{13}C NMR (151 MHz, MeOD) δ 174.1, 162.3, 118.7, 106.9, 96.2, 80.7, 76.8, 50.3, 46.9, 39.6, 28.3, 27.7, 25.8, 21.0, 7.7.

IR (film): 3395, 2967, 1759, 1697, 1104, 1020 cm^{-1}.

HRMS (APCI): *m/z* Calc. for $C_{15}H_{21}O_5$ $[M+H]^+$: 281.1384, found: 281.1388.

TLC (10% MeOH in CH_2Cl_2): R_f = 0.30.

Table S3. Comparison of ^{1}H and ^{13}C NMR data for isolated [4] and synthetic phaeocaulisin A.

Position	^{1}H-NMR (δ, ppm)			^{13}C-NMR (δ, ppm)		
	Isolated	Synthetic	△δ^{a}	Isolated	Synthetic	△δ^{a}
1				96.3	96.2	–0.1
2	1.58, 2.02	1.64, 2.08	+0.06, +0.06	28.5	28.3	–0.2
3	1.82, 1.76	1.88, 1.81^{b}	+0.06, +0.05	39.8	39.6	–0.2
4				80.8	80.7	–0.1
5	2.56	2.62	+0.06	50.4	50.3	–0.1
6	2.87, 2.78	2.93, 2.84	+0.06, +0.06	21.1	21.0	–0.1
7				162.4	162.3	–0.1
8				107.1	106.9	–0.2
9	2.22, 2.12	2.27, 2.16	+0.05, +0.04	47.1	46.9	–0.2
10				76.9	76.8	–0.1
11				118.8	118.7	–0.1
12				174.3	174.1	–0.2
13	1.74	1.79	+0.05	7.9	7.7	–0.2
14	1.11	1.17	+0.06	25.9	25.8	–0.1
15	1.34	1.40	+0.06	27.9	27.7	–0.2

^{a} The differences between chemical shifts may be due to choice of reference. The isolation group used TMS as reference when we used MeOD as reference. Procter group reported the same issue in their synthesis paper. [5] ^{b} The chemical shift of the center of this peak was estimated based on HSQC.

Table S4. Comparison of ^{1}H and ^{13}C NMR data for phaeocaulisin A synthesized by Procter group [5] and phaeocaulisin A synthesized by our group.

Position	^{1}H-NMR (δ, ppm)			^{13}C-NMR (δ, ppm)		
	Ref. 5	This work	$\triangle\delta^a$	Ref. 5	This work	$\triangle\delta^a$
1				96.2	96.2	0.0
2	1.64, 2.08	1.64, 2.08	0.00, 0.00	28.3	28.3	0.0
3	1.88,<br>1.85-1.74	1.88,<br>1.81	0.00	39.6	39.6	0.0
4				80.7	80.7	0.0
5	2.62	2.62	0.00	50.3	50.3	0.0
6	2.93, 2.83	2.93, 2.84	0.00, +0.01	21.0	21.0	0.0
7				162.3	162.3	0.0
8				106.9	106.9	0.0
9	2.27, 2.16	2.27, 2.16	0.00, 0.00	46.9	46.9	0.0
10				76.8	76.8	0.0
11				118.7	118.7	0.0
12				174.1	174.1	0.0
13	1.79	1.79	0.00	7.7	7.7	0.0
14	1.17	1.17	0.00	25.8	25.8	0.0
15	1.40	1.40	0.00	27.7	27.7	0.0

1.2 Synthesis of γ-ketoesters via cyclopropanol ring-opening carbonylation

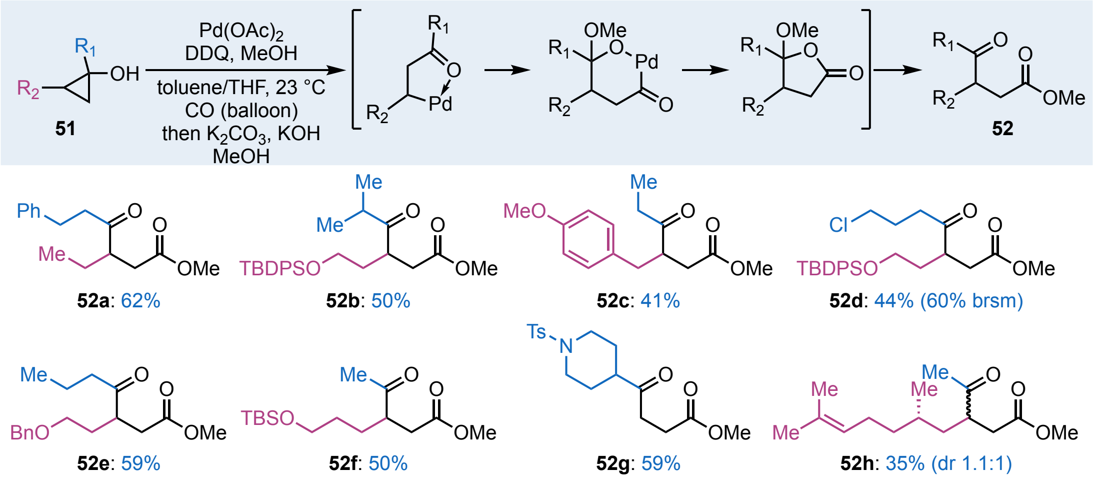

Known cyclopropanols **51a**,[6] **51b**,[7] **51c**,[8] **51e**,[9] **51f**,[10] **51g**,[11] and **51h**[12] and a previously unknown cyclopropanol **51d** were prepared using Kulinkovich reaction from the corresponding methyl/ethyl esters and Grignard reagents (or alkenes and *c*-C_5H_9MgCl).

Representative procedure for Kulinkovich reaction: Preparation of 51d

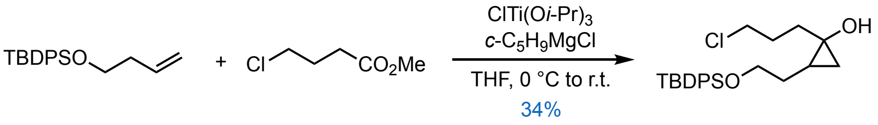

Under argon, ClTi(O*i*-Pr)$_3$ (1.0 equiv., 0.24 mL, 1.0 mmol) was added to a flame-dried flask charged with 4-(*tert*-butyldiphenylsiloxy)-1-butene [13] (1.5 equiv., 0.47 g, 1.5 mmol), methyl 4-chlorobutyrate (1 equiv., 0.13 mL, 1.0 mmol) and anhydrous tetrahydrofuran (10 mL). Then cyclopentylmagnesium chloride (2.2 equiv., 2.0 M in diethyl ether, 1.1 mL, 2.2 mmol) was added via syringe pump over 45 minutes at 0 °C. After completion of addition, the reaction mixture was stirred at 23 °C for 80 minutes before addition of saturated NH_4Cl aqueous solution (0.4 mL). The reaction mixture was stirred until it became a grey suspension, then it was filtered over Celite (washed by EtOAc) and concentrated under reduced pressure. The residue was purified by flash silica gel column chromatography (20% EtOAc in hexanes) to give cyclopropanol **51d** (140 mg, 34%) as colorless oil. The product was dissolved in 3.2 mL anhydrous toluene to prepare its 0.10 M solution for the next step.

^{1}H NMR (400 MHz, CDCl$_3$) δ 7.68 (ddt, *J* = 6.4, 3.7, 1.6 Hz, 4H), 7.47–7.36 (m, 6H), 3.72 (t, *J* = 6.6 Hz, 2H), 3.61 (t, *J* = 6.5 Hz, 2H), 2.00 (dqd, *J* = 7.4, 6.5, 1.1 Hz, 2H), 1.76–1.62 (m,

2H), 1.58–1.49 (m, 1H), 1.39–1.28 (m, 1H), 1.14–1.07 (m, 1H), 1.06 (s, 9H), 0.83 (ddd, J = 10.1, 5.4, 1.3 Hz, 1H), 0.09 (dd, J = 6.5, 5.4 Hz, 1H).

^{13}C NMR (101 MHz, $CDCl_3$) δ 135.8(*2), 134.1(*2), 129.8(*2), 127.8(*2), 63.8, 58.2, 45.4, 32.8, 31.6, 29.3, 27.1, 22.9, 19.5, 19.4.

IR (film): 3338, 3070, 2930, 2857, 1428, 1110, 823, 739, 702, 614 cm^{-1}.

HRMS (APCI): *m/z* Calc. for $C_{24}H_{34}ClO_2Si$ $[M+H]^+$: 417.2011, found: 417.2018.

TLC (20% EtOAc in hexanes): R_f = 0.3.

General procedure for palladium(II)-catalyzed ring-opening carbonylation

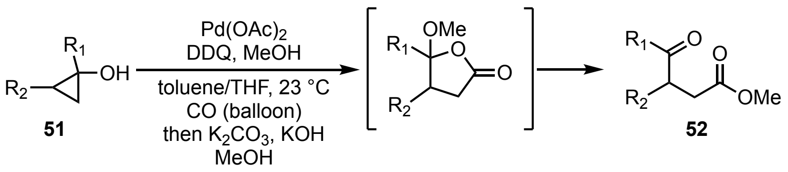

Under a balloon pressure of CO, a solution of the cyclopropanol in toluene (0.10 M, 1 equiv., 1.5 mL, 1.5 mmol) was rapidly added to an oven-dried 10 mL microwave tube charged with palladium(II) acetate (10 mol%, 3.4 mg, 15 μmol), 2,3-dichloro-5,6-dicyano-1,4-benzoquinone (2.0 equiv., 68 mg, 0.3 mmol), anhydrous methanol (12 equiv., 75 μL, 1.8 mmol) and THF (1.5 mL). The reaction mixture was stirred vigorously at 23 °C overnight (or for 3 hours), then the reaction mixture was exposed to open air and diluted with anhydrous methanol (3.0 mL). Potassium carbonate (0.15 g) and potassium hydroxide (0.10 g) were added sequentially. After 5 minutes, water (4 mL) was added, and the desired organic material was extracted with diethyl ether (5 mL×4). The combined organic layer was washed with brine, NH_4Cl solution and brine sequentially, dried over sodium sulfate, filtered, and concentrated under reduced pressure. The residue was purified by flash column chromatography to give the corresponding ketoester product.

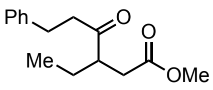

52a: 23.2 mg, 62%; colorless oil; purified by column chromatography (9% ethyl acetate in hexanes).

^{1}H NMR (600 MHz, CDCl$_3$) δ 7.30–7.26 (m, 2H), 7.22–7.17 (m, 3H), 3.64 (s, 3H), 2.96–2.82 (m, 5H), 2.77 (dd, *J* = 16.8, 9.9 Hz, 1H), 2.36 (dd, *J* = 16.8, 4.4 Hz, 1H), 1.62 (tt, *J* = 13.6, 7.5 Hz, 1H), 1.44 (dp, *J* = 14.5, 7.4 Hz, 1H), 0.85 (t, *J* = 7.5 Hz, 3H).

^{13}C NMR (151 MHz, CDCl$_3$) δ 212.0, 173.2, 141.4, 128.6, 128.5, 126.2, 51.9, 48.7, 44.3, 34.7, 29.6, 24.6, 11.4.

IR (film): 3027, 2929, 1734, 1711, 1454, 1436, 1359, 1246, 1196, 1176, 750, 700 cm^{-1}.

HRMS (ESI): *m/z* Calc. for $C_{15}H_{21}O_3$ [M+H]$^+$: 249.1485, found: 249.1486.

TLC (20% EtOAc in hexanes): R$_f$ = 0.4.

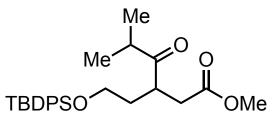

52b (on a 0.10 mmol scale): 22.2 mg, 50%; colorless oil; purified by column chromatography (9% ethyl acetate in hexanes).

^{1}H NMR (400 MHz, CDCl$_3$) δ 7.67–7.62 (m, 4H), 7.47–7.35 (m, 6H), 3.71–3.58 (m, 2H), 3.63 (s, 3H), 3.37 (ddt, *J* = 9.9, 8.7, 4.4 Hz, 1H), 2.81 (hept, *J* = 6.9 Hz, 1H), 2.69 (dd, *J* = 16.8, 9.9 Hz, 1H), 2.28 (dd, *J* = 16.8, 4.3 Hz, 1H), 1.85 (dddd, *J* = 13.8, 7.6, 6.0, 4.6 Hz, 1H), 1.48 (ddt, *J* = 14.0, 8.5, 5.4 Hz, 1H), 1.14 (d, *J* = 7.0 Hz, 3H), 1.08–1.04 (m, 12H).

^{13}C NMR (101 MHz, CDCl$_3$) δ 216.5, 172.9, 135.7, 135.7, 133.6, 133.5, 130.0, 127.9, 61.3, 51.8, 42.3, 39.8, 34.6, 34.0, 27.0, 19.3, 19.0, 18.1.

IR (film): 2931, 2857, 1738, 1711, 1471, 1428, 1169, 1106, 1036, 823, 739, 703, 688, 614 cm^{-1}.

HRMS (APCI): *m/z* Calc. for $C_{26}H_{37}O_4Si$ [M+H]$^+$: 441.2456, found: 441.2465.

TLC (20% EtOAc in hexanes): R$_f$ = 0.6.

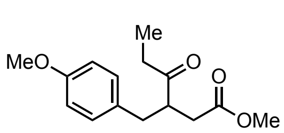

52c (reaction time: 3 h): 16.7 mg, 41%; colorless oil; purified by column chromatography (20% ethyl acetate in hexanes).

^{1}H NMR (600 MHz, CDCl$_3$) δ 7.07–7.03 (m, 2H), 6.84–6.81 (m, 2H), 3.79 (s, 3H), 3.61 (s, 3H), 3.21 (dtd, *J* = 10.2, 7.8, 4.2 Hz, 1H), 2.82 (dd, *J* = 13.6, 7.4 Hz, 1H), 2.77 (dd, *J* = 17.1,

10.2 Hz, 1H), 2.54 (dd, J = 13.6, 8.2 Hz, 1H), 2.51 (dq, J = 18.2, 7.3 Hz, 1H), 2.34 (dd, J = 17.1, 4.2 Hz, 1H), 2.25 (dq, J = 18.2, 7.2 Hz, 1H), 0.97 (t, J = 7.2 Hz, 3H).

^{13}C NMR (151 MHz, CDCl$_3$) δ 213.8, 173.0, 158.5, 130.5, 130.1, 114.2, 55.4, 51.9, 49.3, 37.5, 36.8, 35.5, 7.6.

IR (film): 2938, 2836, 1733, 1712, 1512, 1200, 1177, 1033, 822 cm^{-1}.

HRMS (ESI): *m/z* Calc. for $C_{15}H_{21}O_4$ [M+H]$^+$: 265.1434, found: 265.1437.

TLC (33% EtOAc in hexanes): R$_f$ = 0.6.

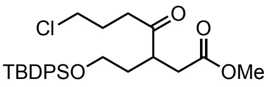

52d: 31.3 mg, 44% (60% b.r.s.m.); colorless oil; purified by column chromatography (9% ethyl acetate in hexanes).

^{1}H NMR (400 MHz, CDCl$_3$) δ 7.64 (ddt, J = 6.4, 3.7, 1.6 Hz, 4H), 7.47–7.36 (m, 6H), 3.71–3.60 (m, 2H), 3.64 (s, 3H), 3.54 (t, J = 6.4 Hz, 2H), 3.20 (dddd, J = 10.1, 7.5, 5.8, 4.1 Hz, 1H), 2.78–2.69 (m, 3H), 2.33 (dd, J = 17.0, 4.1 Hz, 1H), 2.08–1.98 (m, 2H), 1.83 (ddt, J = 14.1, 6.9, 5.8 Hz, 1H), 1.61–1.51 (m, 1H), 1.06 (s, 9H).

^{13}C NMR (101 MHz, CDCl$_3$) δ 212.1, 173.0, 135.7, 133.5, 130.0(*2), 128.0, 127.9, 61.3, 51.9, 44.6, 44.2, 39.2, 35.1, 34.1, 27.0, 26.4, 19.3.

IR (film): 2930, 2857, 1735, 1713, 1428, 1361, 1172, 1106, 823, 738, 703, 688, 614 cm^{-1}.

HRMS (APCI): *m/z* Calc. for $C_{26}H_{36}ClO_4Si$ [M+H]$^+$: 475.2066, found: 475.2077.

TLC (20% EtOAc in hexanes): R$_f$ = 0.5.

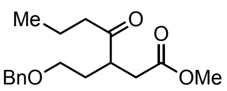

52e: 26.0 mg, 59%; colorless oil; purified by column chromatography (20% ethyl acetate in hexanes).

^{1}H NMR (600 MHz, CDCl$_3$) δ 7.36–7.27 (m, 5H), 4.46 (s, 2H), 3.63 (s, 3H), 3.49–3.42 (m, 2H), 3.14 (dtd, J = 9.6, 6.7, 4.6 Hz, 1H), 2.75 (dd, J = 16.8, 9.6 Hz, 1H), 2.58–2.46 (m, 2H), 2.37 (dd, J = 16.8, 4.7 Hz, 1H), 1.92 (dtd, J = 14.3, 6.8, 5.5 Hz, 1H), 1.72–1.65 (m, 1H), 1.63–1.51 (m, 2H), 0.88 (t, J = 7.4 Hz, 3H).

^{13}C NMR (151 MHz, CDCl$_3$) δ 212.9, 172.9, 138.2, 128.6, 127.9, 73.2, 67.6, 51.9, 44.6, 44.5, 35.3, 31.6, 17.0, 13.9.

IR (film): 2955, 2872, 1735, 1711, 1454, 1436, 1360, 1204, 1173, 1101, 738, 699 cm^{-1}.

HRMS (ESI): *m/z* Calc. for $C_{17}H_{24}O_4Na$ $[M+Na]^+$: 315.1567, found: 315.1582.

HRMS (APCI): *m/z* Calc. for $C_{16}H_{21}O_3$ $[M-OCH_3]^+$: 261.1485, found: 261.1489;

m/z Calc. for $C_{10}H_{17}O_3$ $[M-OBn]^+$: 185.1172, found: 185.1176;

m/z Calc. for $C_{16}H_{21}O_4$ $[M-CH_3]^-$: 277.1445, found: 277.1445.

TLC (33% EtOAc in hexanes): $R_f = 0.6$.

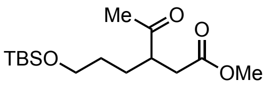

52f: 22.5 mg, 50%; colorless oil; purified by column chromatography (9% ethyl acetate in hexanes).

^{1}H NMR (600 MHz, CDCl$_3$) δ 3.65 (s, 3H), 3.59 (ddd, *J* = 6.1, 5.1, 1.6 Hz, 2H), 3.04–2.98 (m, 1H), 2.75 (dd, *J* = 16.8, 9.8 Hz, 1H), 2.35 (dd, *J* = 16.9, 4.5 Hz, 1H), 2.23 (s, 3H), 1.72–1.64 (m, 1H), 1.54–1.44 (m, 3H), 0.88 (s, 9H), 0.03 (s, 6H).

^{13}C NMR (151 MHz, CDCl$_3$) δ 211.0, 173.1, 62.7, 51.9, 47.8, 35.0, 30.1, 29.6, 27.7, 26.1, 18.5, -5.2.

IR (film): 2952, 2929, 2856, 1738, 1715, 1436, 1355, 1253, 1162, 1098, 836, 776 cm^{-1}.

HRMS (APCI): *m/z* Calc. for $C_{15}H_{31}O_4Si$ $[M+H]^+$: 303.1986, found: 303.1991.

TLC (20% EtOAc in hexanes): $R_f = 0.4$.

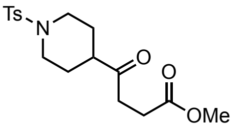

52g (extraction was done with ethyl acetate): 31.3 mg, 59%; white solid; purified by column chromatography (50% ethyl acetate in hexanes).

^{1}H NMR (400 MHz, CDCl$_3$) δ 7.65–7.61 (m, 2H), 7.34–7.29 (m, 2H), 3.75–3.67 (m, 2H), 3.64 (s, 3H), 2.71–2.65 (m, 2H), 2.60–2.54 (m, 2H), 2.43 (s, 3H), 2.40 (td, *J* = 11.6, 2.8 Hz, 2H), 2.31 (tt, *J* = 11.2, 3.9 Hz, 1H), 1.97–1.88 (m, 2H), 1.73 (dtd, *J* = 13.6, 11.2, 4.0 Hz, 2H),.

^{13}C NMR (101 MHz, CDCl$_3$) δ 209.7, 173.3, 143.8, 133.3, 129.8, 127.8, 52.0, 47.5, 45.7, 35.0, 27.7, 27.1, 21.7.

IR (film): 2951, 2847, 1735, 1709, 1597, 1437, 1334, 1160, 1094, 928, 818, 726, 650 cm^{-1}.

HRMS (APCI): *m/z* Calc. for $C_{17}H_{24}NO_5S$ $[M+H]^+$: 354.1370, found: 354.1376.

TLC (50% EtOAc in hexanes): R_f = 0.4.

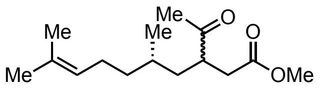

52h: 13.4 mg, 35%, *dr* = 1.1:1; colorless oil; purified by column chromatography (9% ethyl acetate in hexanes).

^{1}H NMR (600 MHz, CDCl$_3$) δ 5.06 (tdd, *J* = 7.1, 2.9, 1.4 Hz, 1H), 3.65 (s+s, 3H), 3.09–3.01 (m, 1H), 2.71 (dt, *J* = 17.0, 10.5 Hz, 1H), 2.35 (dt, *J* = 16.8, 3.8 Hz, 1H), 2.23 (s+s, 3H), 2.04–1.88 (m, 2H), 1.68 (brs, 3H), 1.60 (brs, 3H), 1.61–1.56 (m, 0.5H), 1.46–1.09 (m, 4.5H), 0.93 (d, *J* = 6.6 Hz, 1.4H), 0.91 (d, *J* = 6.4 Hz, 1.6H).

^{13}C NMR (151 MHz, CDCl$_3$) δ 211.6, 211.4, 173.2, 173.1, 131.8(*2), 124.5(*2), 51.9 (*2), 45.9(*2), 39.2, 38.6, 37.5, 36.8, 36.0, 35.1, 30.5, 30.3, 29.9, 29.5, 25.9(*2), 25.5, 25.4, 20.0, 19.4, 17.9, 17.8.

IR (film): 2954, 2914, 2852, 1737, 1715, 1436, 1352, 1162 cm^{-1}.

HRMS (APCI): *m/z* Calc. for $C_{15}H_{27}O_3$ $[M+H]^+$: 255.1955, found: 255.1953.

TLC (20% EtOAc in hexanes): R_f = 0.5.

1.3 Materials and methods for biological evaluations

MDA-MB-468, SKBR3 and MDA-MB-231 were obtained from American Type Culture Collection (ATCC) (Manassas, VA) and cultured in Dulbecco's Modified Eagle Medium (Corning, 10-017-cv) supplemented with 10% fetal bovine serum (FBS) and 1% of streptomycin (100 U/ml) and penicillin (100 U/ml) at a humidity incubator at 37°C with 5% CO2. For colony formation assays, 3000, 2000 and 800 cells of MDA-MB-468, SKBR3 and MDA-MB-231 respectively were seeded into the individual wells of a 6-well plate followed by drug treatment 24 hours after cell seeding. Cells were fixed and stained with crystal violet after 2-3 weeks' cell culture. Any colony of at least 50 cells was counted and the total number of the

colonies was normalized by the corresponding untreated control to get the cell survival percentage for each condition. IC_{50} was calculated with GraphPad Prism (version 9, GraphPad Software).

Part 2. X-ray structure and analysis data.

Compound **34– CCDC 2292481**

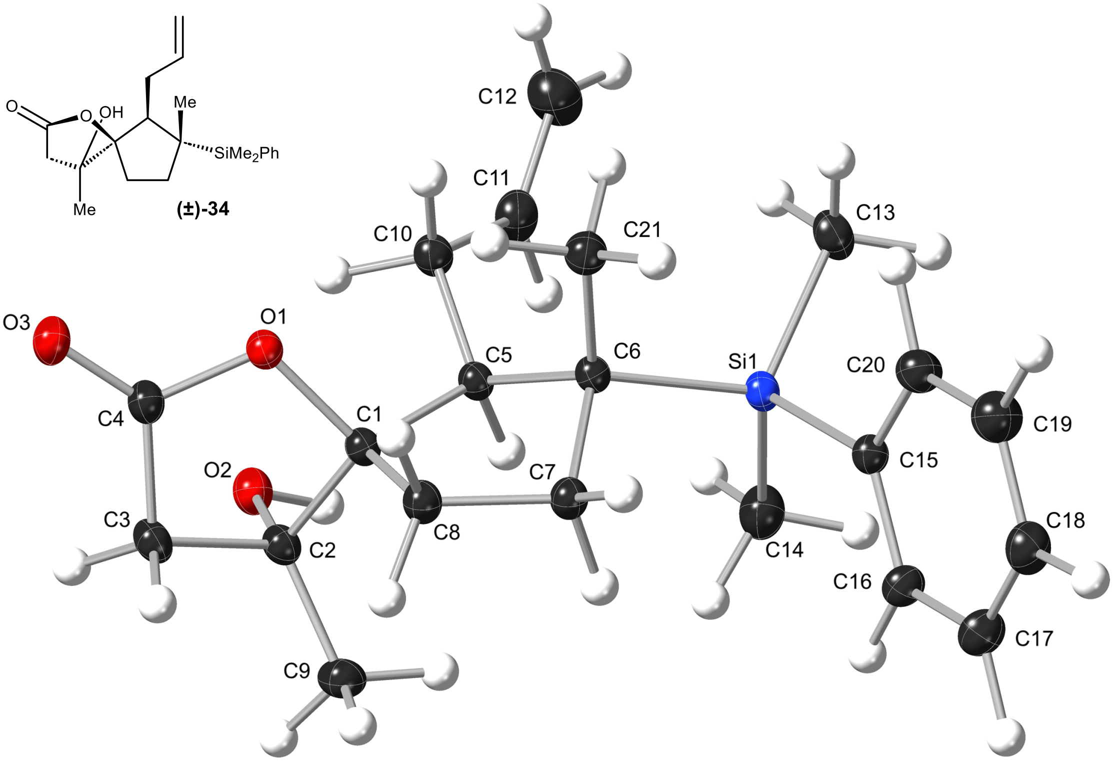

Figure S3. Crystal data and structure refinement for compound **(±)-34**.

Crystal data: $C_{21}H_{30}O_3Si$, M_r = 358.56, orthorhombic, $Pna2_1$ (No. 33), a = 27.6547(4) Å, b = 8.89775(13) Å, c = 8.27744(11) Å, $\alpha = \beta = \gamma = 90^{\circ}$, V = 2036.78(5) Å^3, T = 100.0(2) K, Z = 4, Z' = 1, μ(Cu K_a) = 1.54178, 37645 reflections measured, 4180 unique (R_{int} = 0.0489) which were used in all calculations. The final wR_2 was 0.0528 (all data) and R_1 was 0.0207 (I≥2 σ(I)).

Compound **49**– **CCDC 2292485**

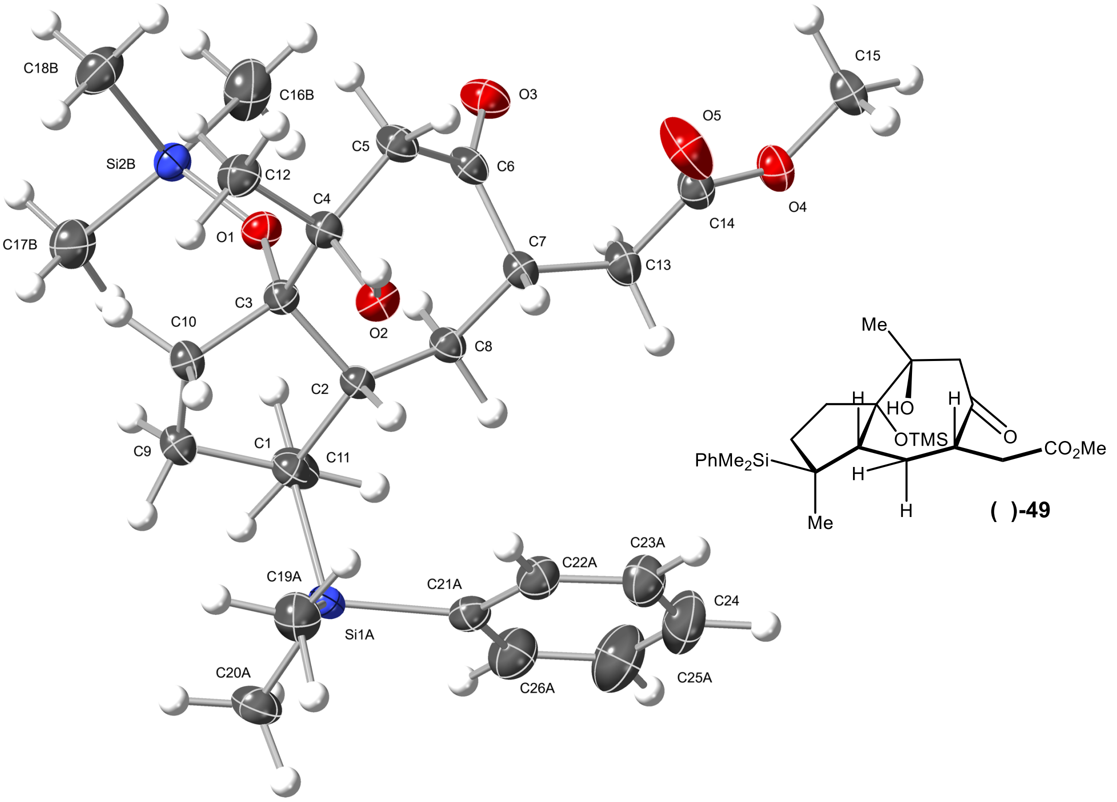

Figure S4. Crystal data and structure refinement for compound **(±)-49**.

Crystal data: $C_{26}H_{42}O_5Si_2$, M_r = 490.77, monoclinic, *C*2/*c* (No. 15), a = 20.26680(18) Å, b = 20.39212(17) Å, c = 13.43292(13) Å, β = 102.019(1)°, $\alpha = \gamma$ = 90°, V = 5429.89(9) Å^3, T = 173.03(10) K, Z = 8, Z' = 1, μ(Cu K_a) = 1.448, 84619 reflections measured, 5299 unique (R_{int} = 0.0295) which were used in all calculations. The final wR_2 was 0.1067 (all data) and R_1 was 0.0407 (I≥2 σ(I)).

Compound **48**– **CCDC 2292484**

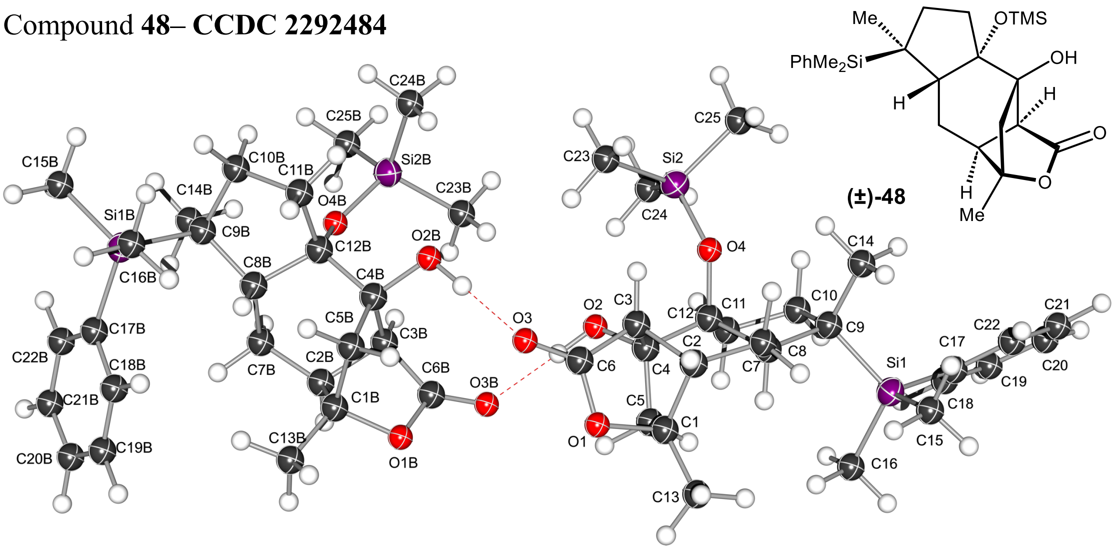

Figure S5. Crystal data and structure refinement for compound **(±)-48**.

Crystal data: $C_{25}H_{38}O_4Si_2$, M_r = 458.73, monoclinic, $P2_1/c$ (No. 14), a = 24.703(2) Å, b = 7.1758(4) Å, c = 30.487(2) Å, β = 104.692(8)°, $\alpha = \gamma$ = 90°, V = 5227.6(7) Å^3, T = 293.3(5) K, Z = 8, Z' = 1, μ(Cu K$_a$) = 1.443, 87928 reflections measured, 9601 unique (R_{int} = 0.1660) which were used in all calculations. The final wR_2 was 0.2243 (all data) and R_1 was 0.0748 (I≥2 σ(I)).

References

(1) Ager, D. J.; Fleming, I.; Patel, S. K. *J. Chem. Soc., Perkin Trans. 1* **1981**, 2520-2526.

(2) Chen, Y.; Turlik, A.; Newhouse, T. R. *J. Am. Chem. Soc.* **2016**, *138*, 1166-1169.

(3) Fraser, R. R.; Mansour, T. S. *J. Org. Chem.* **1984**, *49*, 3442.

(4) Liu, Y.; Ma, J.; Zhao, Q.; Liao, C.; Ding, L.; Chen, L.; Zhao, F.; Qiu, F. *J. Nat. Prod.* **2013**, *76*, 1150-1156.

(5) Péter, Á.; Crisenza, G. E. M.; Procter, D. J. *J. Am. Chem. Soc.* **2022**, *144*, 7457-7464 and supporting information.

(6) Ziegler, D. T.; Steffens, A. M.; Funk, T. W. *Tetrahedron Lett.* **2010**, *51*, 6726-6729.

(7) Barysevich, M. V.; Aniskevich, Y. M.; Hurski, A. *Synlett* **2021**, *32*, 1934-1938.

(8) Ni, J.; Xia, X.; Zheng, W.-F.; Wang, Z. *J. Am. Chem. Soc.* **2022**, *144*, 7889-7900.

(9) Ziegler, D. T.; Steffens, A. M.; Funk, T. W. *Tetrahedron Lett.* **2010**, *51*, 6726-6729.

(10) Masyuk, V. S.; Kozyrkov, Y. Y.; Mineeva, I. V. *Russ. J. Org. Chem.* **2021**, *57*, 1563-1574.

(11) Shen, M.-H.; Lu, X.-L.; Xu, H.-D. *RSC Adv.* **2015**, *5*, 98757-98761.

(12) Lee, J.; Kim, H.; Cha, J. K. *J. Am. Chem. Soc.* **1996**, *118*, 4198-4199.

(13) Kliman, L. T.; Mlynarski, S. N.; Morken, J. P. *J. Am. Chem. Soc.* **2009**, *131*, 13210.

Part 3. ^{1}H and ^{13}C NMR spectra

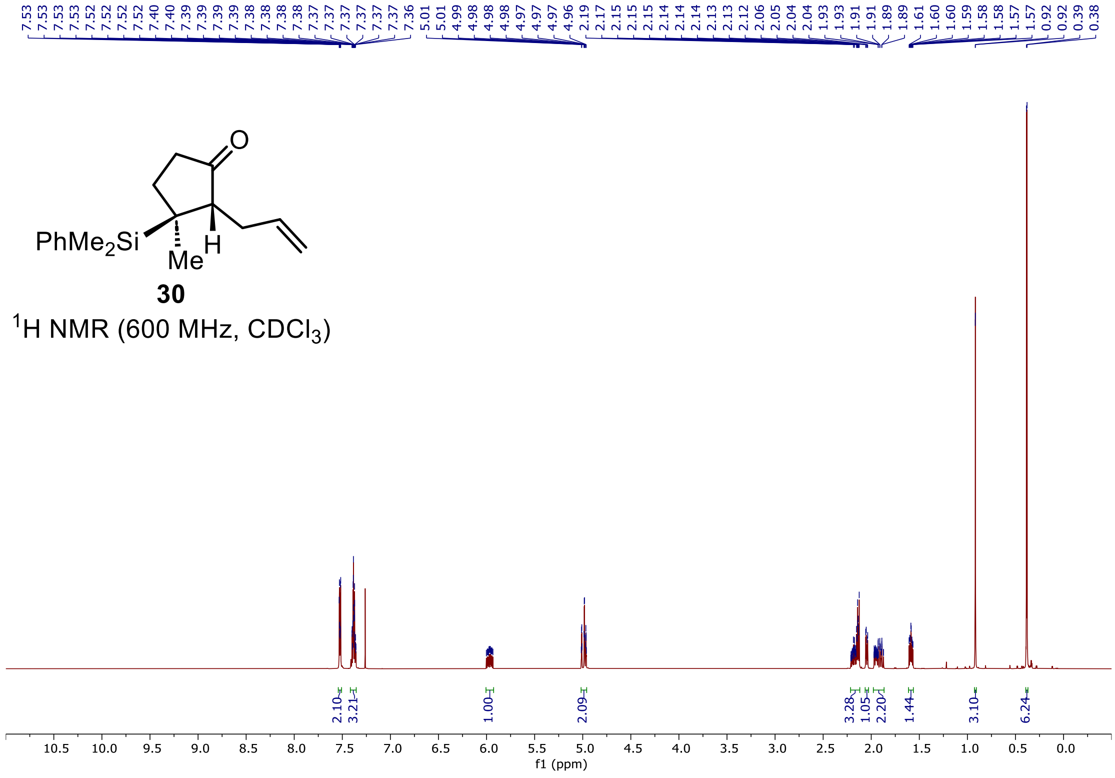

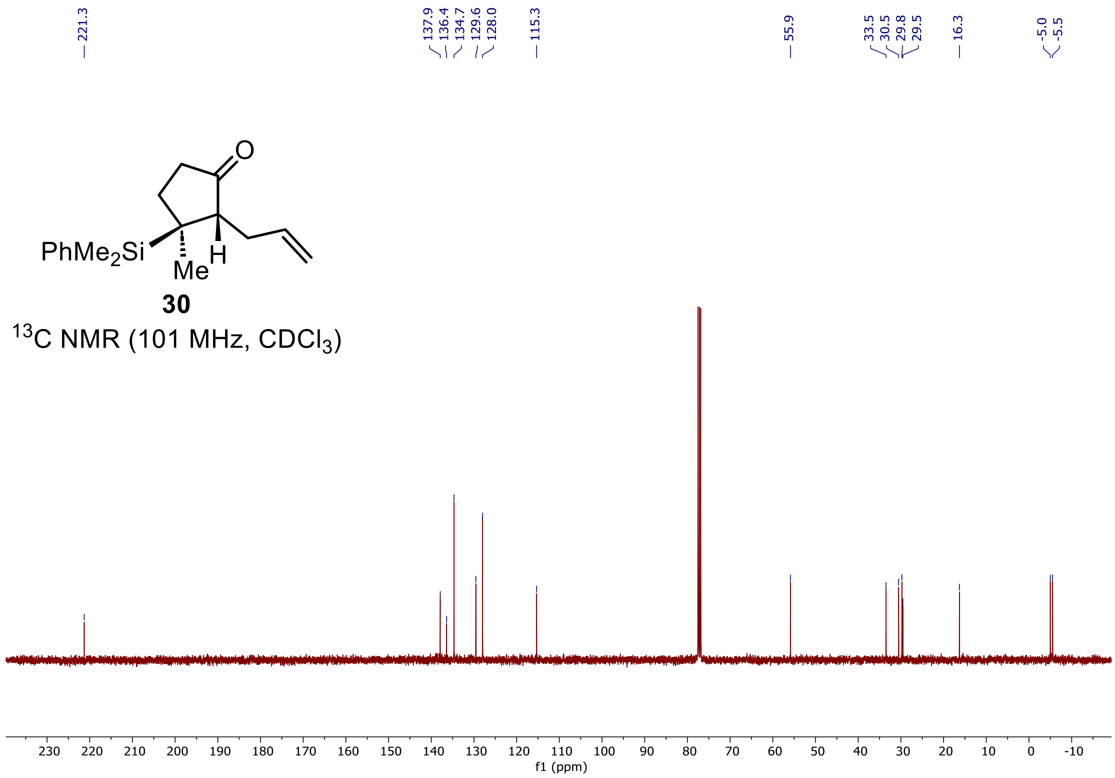

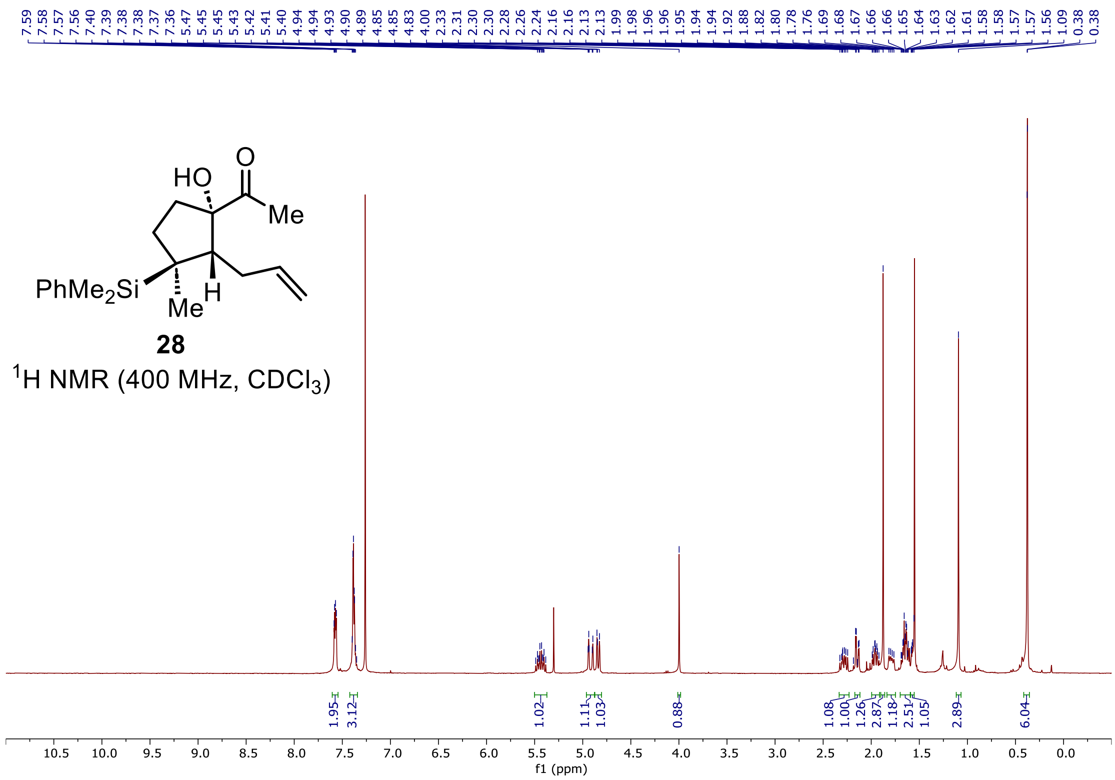
7.59 7.58 7.57 7.56 7.40 7.39 7.38 7.38 7.37 7.36 5.47 5.45 5.45 5.43 5.42 5.41 5.40 4.94 4.94 4.93 4.90 4.89 4.85 4.85 4.83 4.00 2.33 2.31 2.30 2.28 2.26 2.24 2.16 2.16 2.13 2.13 1.99 1.98 1.96 1.96 1.95 1.94 1.94 1.92 1.88 1.82 1.80 1.78 1.76 1.69 1.68 1.67 1.66 1.66 1.65 1.64 1.63 1.62 1.61 1.58 1.58 1.57 1.57 1.56 1.09 0.38 0.38
HO
O
Me
PhMe2Si
Me
H
28
1H NMR (400 MHz, CDCl3)
1.95 3.12 1.02 1.11 1.03 0.88 1.08 1.00 1.26 2.87 1.18 2.51 1.05 2.89 6.04
f1 (ppm)

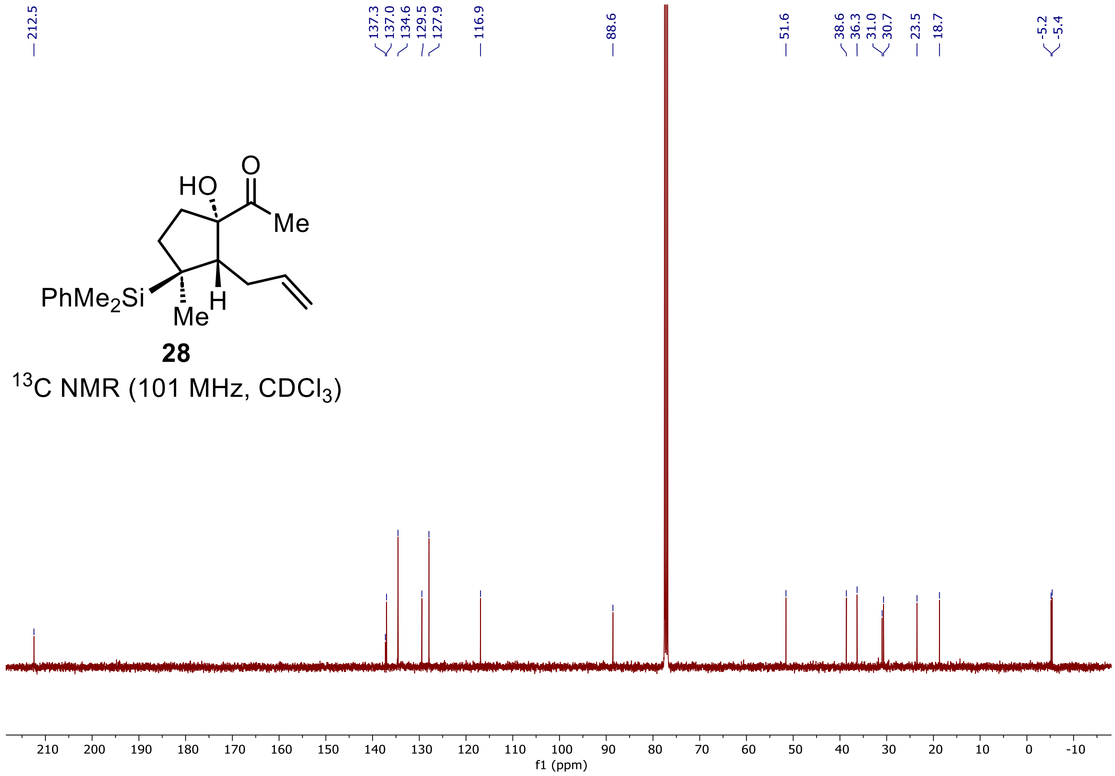
212.5
137.3 137.0 134.6 129.5 127.9
116.9
88.6
51.6
38.6 36.3 31.0 30.7 23.5 18.7
-5.2 -5.4
HO
O
Me
PhMe2Si
Me
H
28
13C NMR (101 MHz, CDCl3)
f1 (ppm)

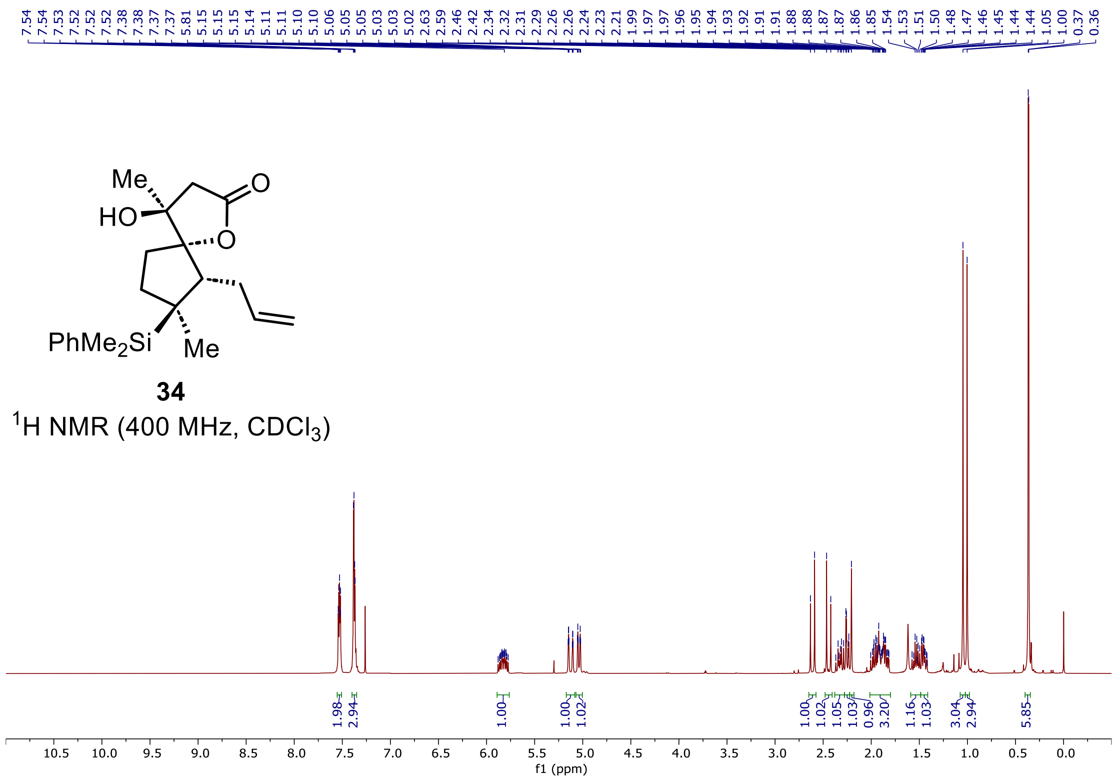
34
1H NMR (400 MHz, CDCl3)
f1 (ppm)

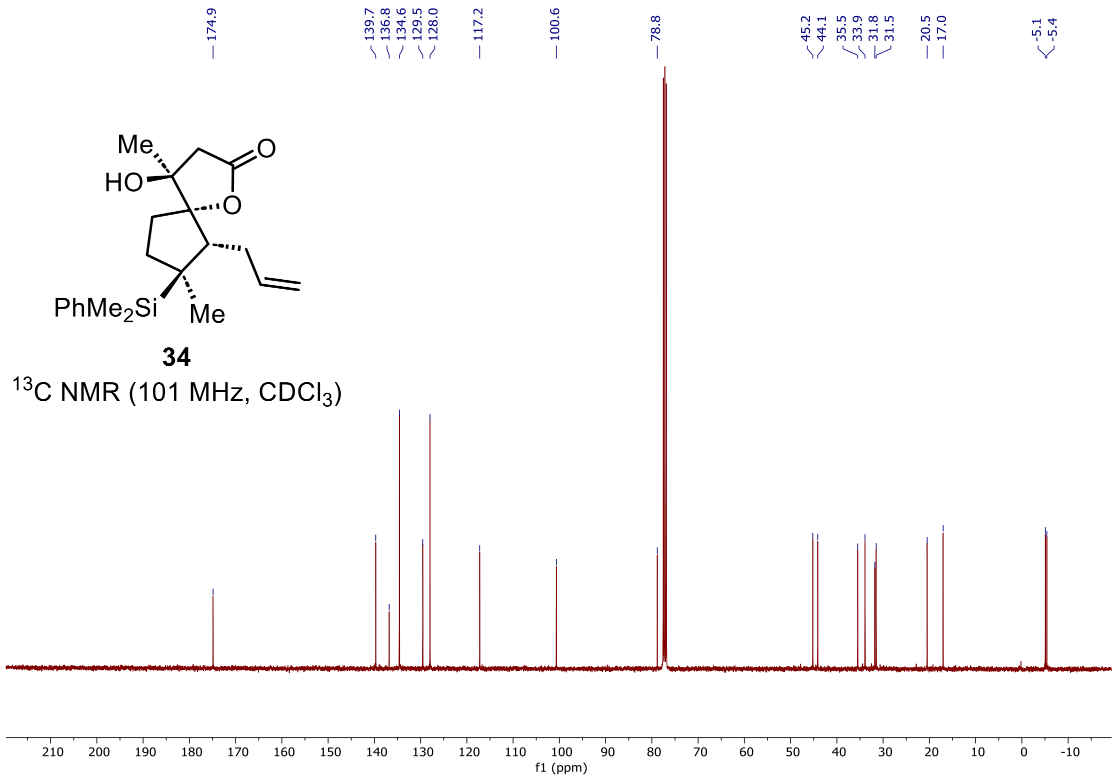
34
13C NMR (101 MHz, CDCl3)
174.9
139.7
136.8
134.6
129.5
128.0
117.2
100.6
78.8
45.2
44.1
35.5
33.9
31.8
31.5
20.5
17.0
-5.1
-5.4
f1 (ppm)

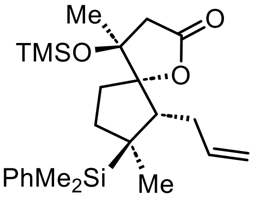

35

^{1}H NMR (400 MHz, $CDCl_3$)

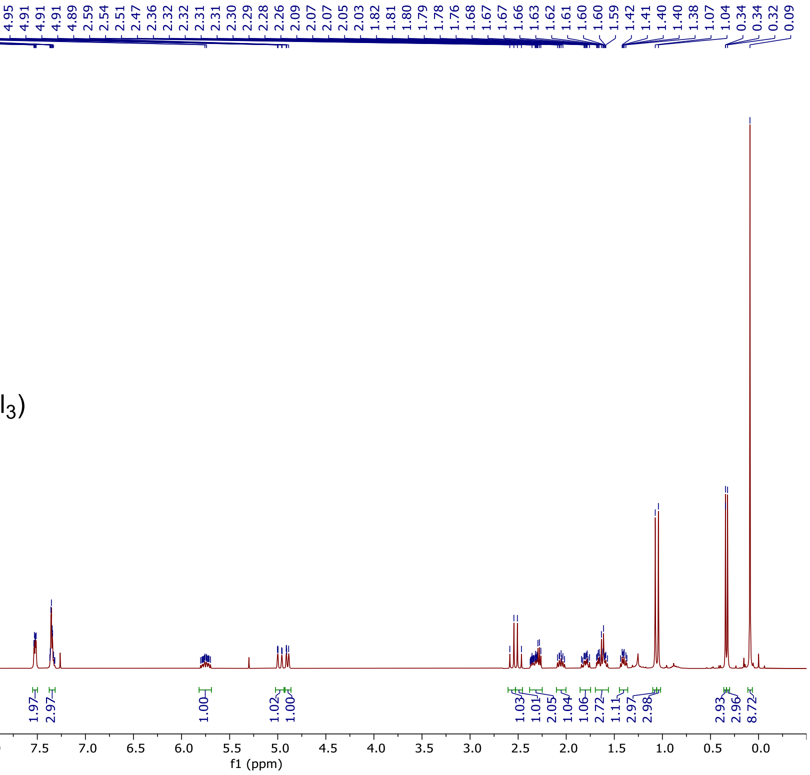

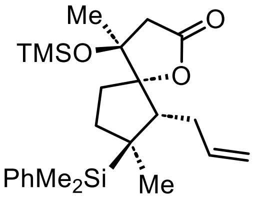

35

^{13}C NMR (101 MHz, $CDCl_3$)

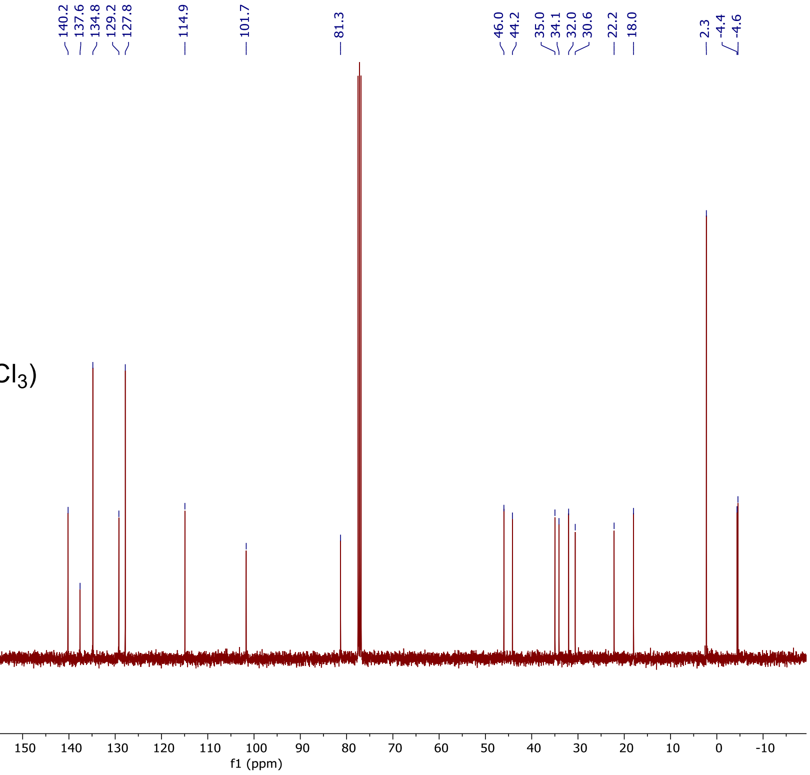

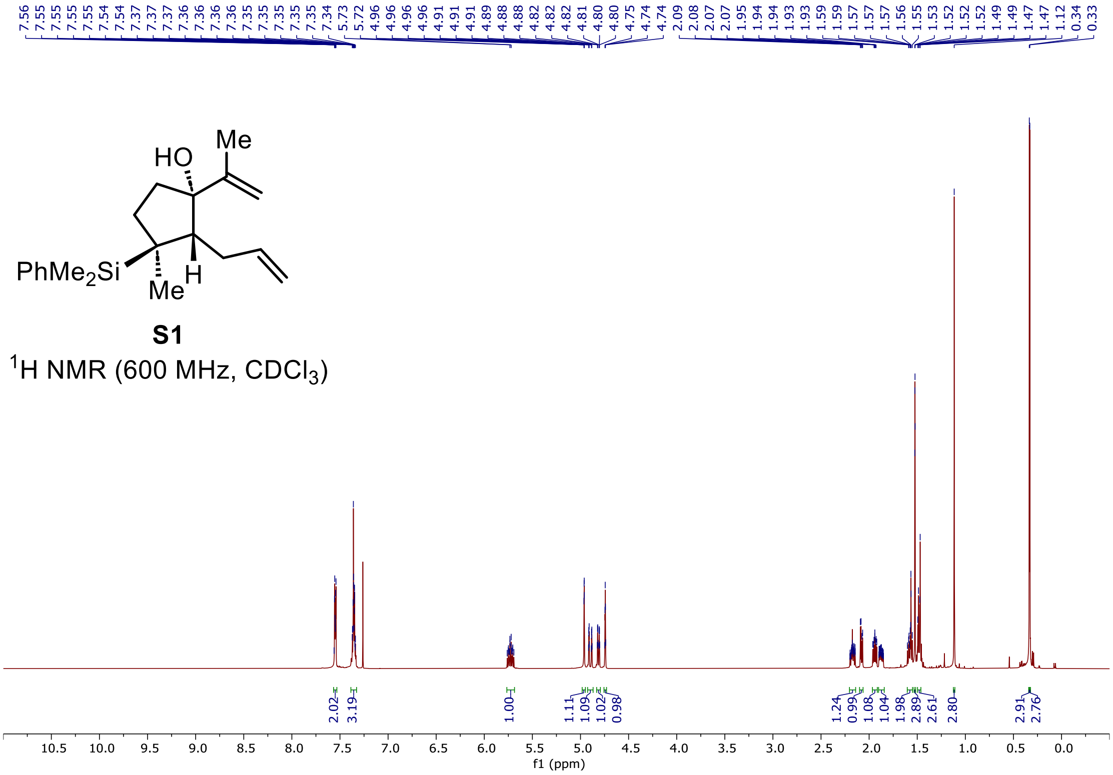

S1

^{1}H NMR (600 MHz, $CDCl_3$)

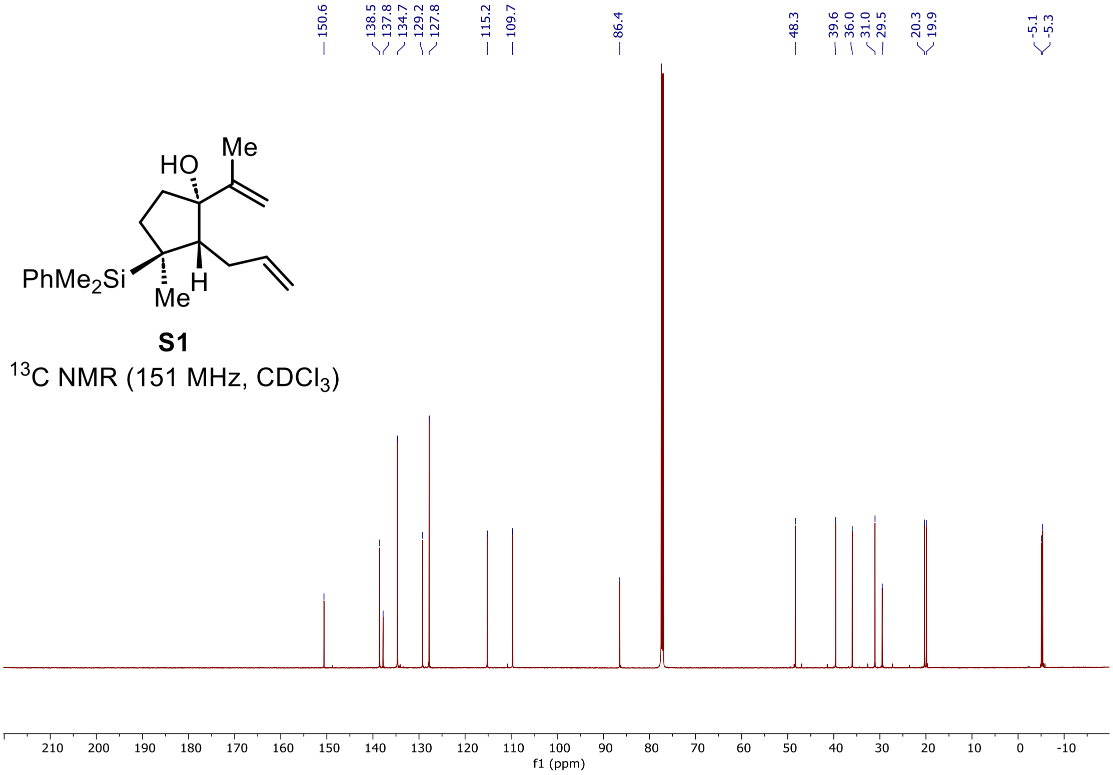

S1

^{13}C NMR (151 MHz, $CDCl_3$)

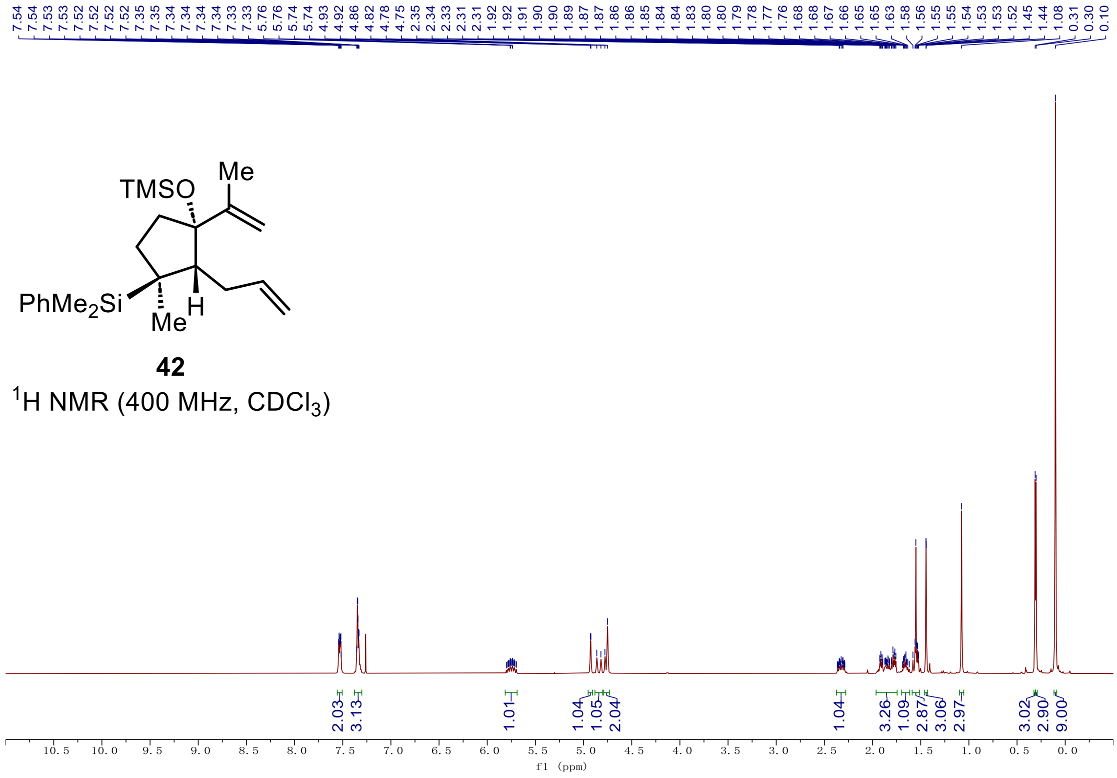
TMSO
Me
PhMe2Si
Me
H
42
1H NMR (400 MHz, CDCl3)

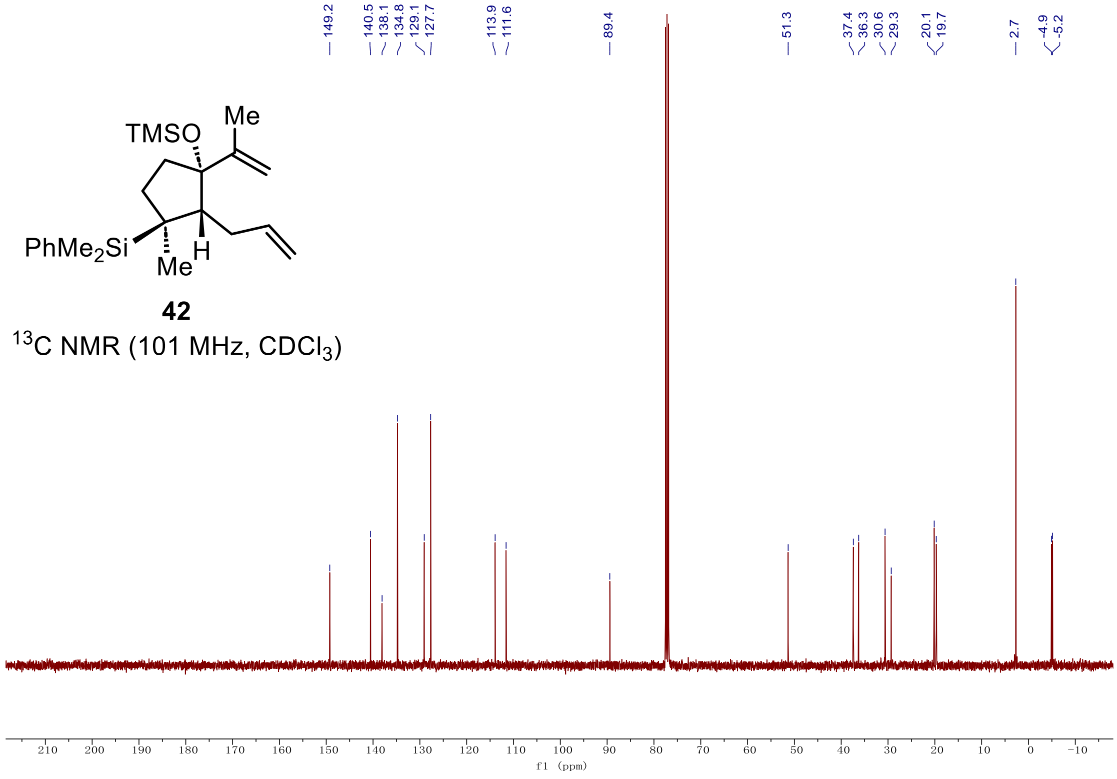
TMSO
Me
PhMe2Si
Me
H
42
13C NMR (101 MHz, CDCl3)

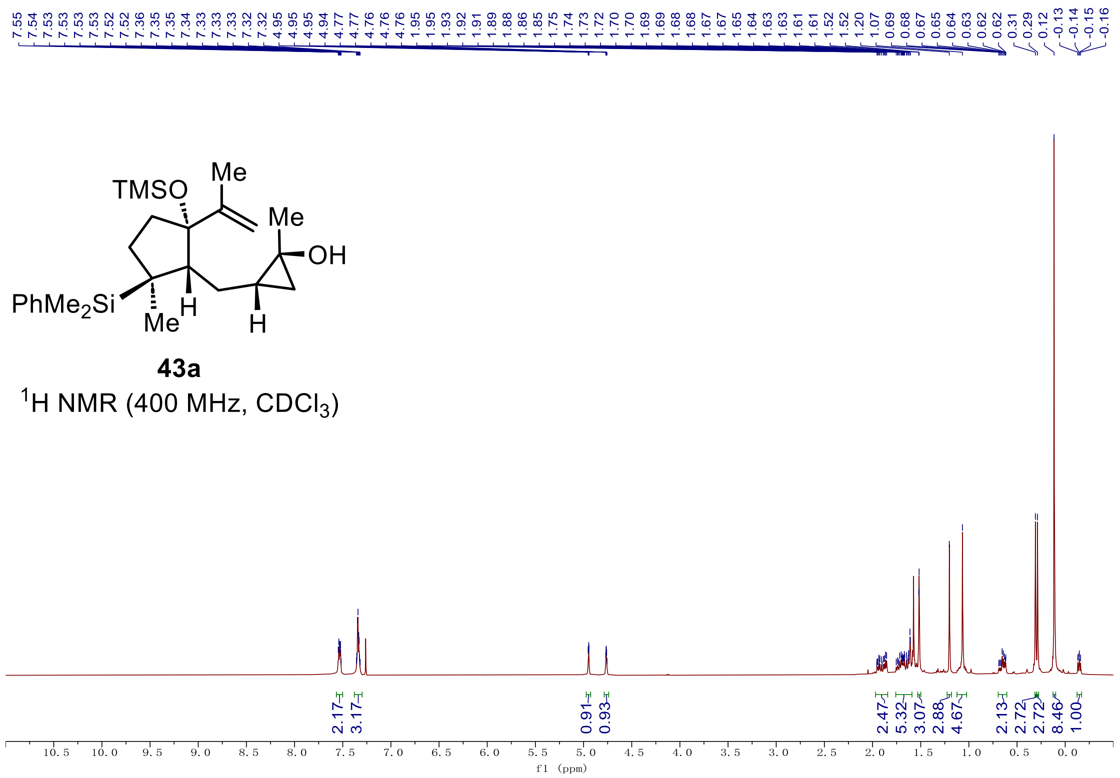
7.55 7.54 7.53 7.53 7.53 7.52 7.52 7.36 7.35 7.35 7.34 7.33 7.33 7.33 7.32 7.32 4.95 4.95 4.95 4.94 4.77 4.77 4.76 4.76 4.76 1.95 1.95 1.93 1.92 1.91 1.89 1.88 1.86 1.85 1.75 1.74 1.73 1.72 1.70 1.70 1.69 1.69 1.68 1.67 1.67 1.65 1.64 1.63 1.63 1.61 1.61 1.52 1.52 1.20 1.07 0.69 0.68 0.67 0.65 0.64 0.63 0.62 0.62 0.31 0.29 0.12 -0.13 -0.14 -0.15 -0.16
TMSO
Me
Me
OH
PhMe2Si
Me
H
H
43a
1H NMR (400 MHz, CDCl3)
2.17 3.17 0.91 0.93 2.47 5.32 3.07 2.88 4.67 2.13 2.72 2.72 8.46 1.00
f1 (ppm)

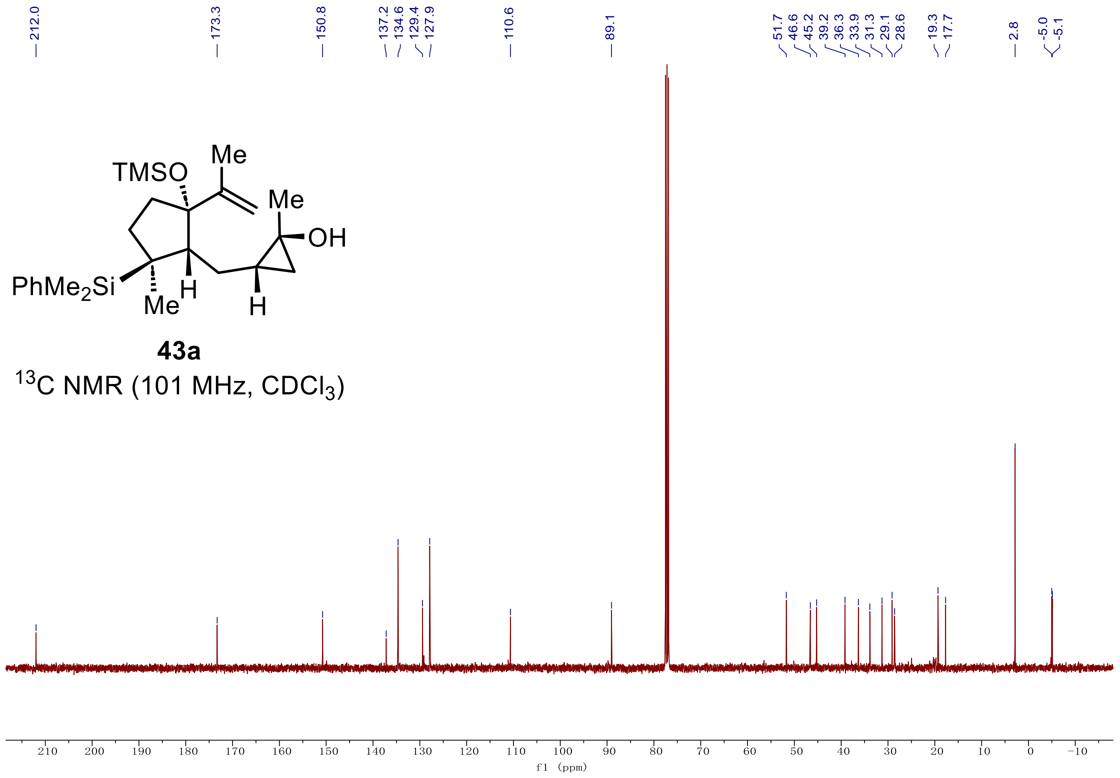
212.0 173.3 150.8 137.2 134.6 129.4 127.9 110.6 89.1 51.7 46.6 45.2 39.2 36.3 33.9 31.3 29.1 28.6 19.3 17.7 2.8 -5.0 -5.1
TMSO
Me
Me
OH
PhMe2Si
Me
H
H
43a
13C NMR (101 MHz, CDCl3)
f1 (ppm)

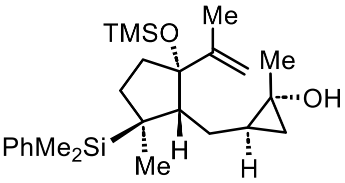

43b

^{1}H NMR (400 MHz, $CDCl_3$)

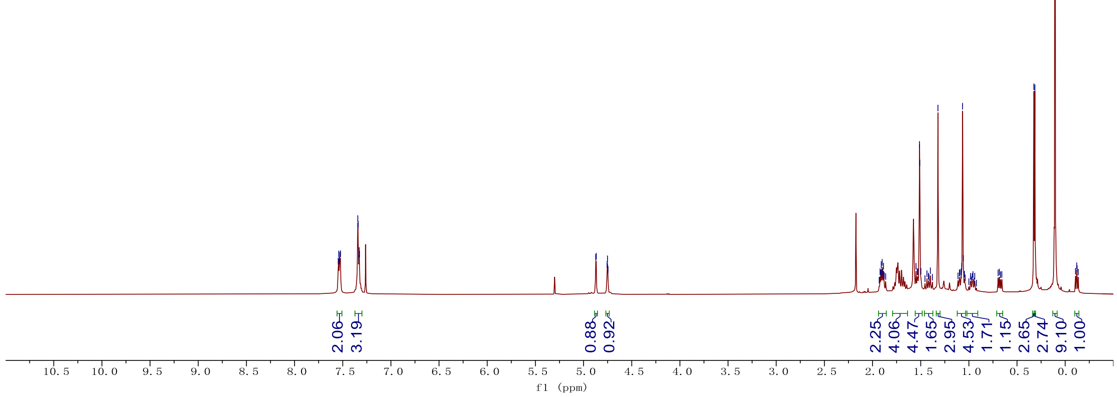

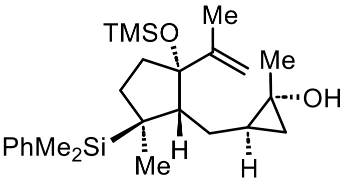

43b

^{13}C NMR (101 MHz, $CDCl_3$)

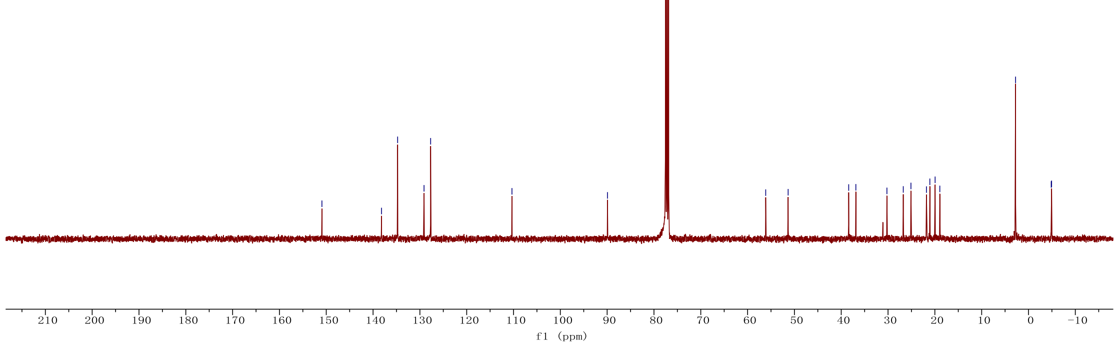

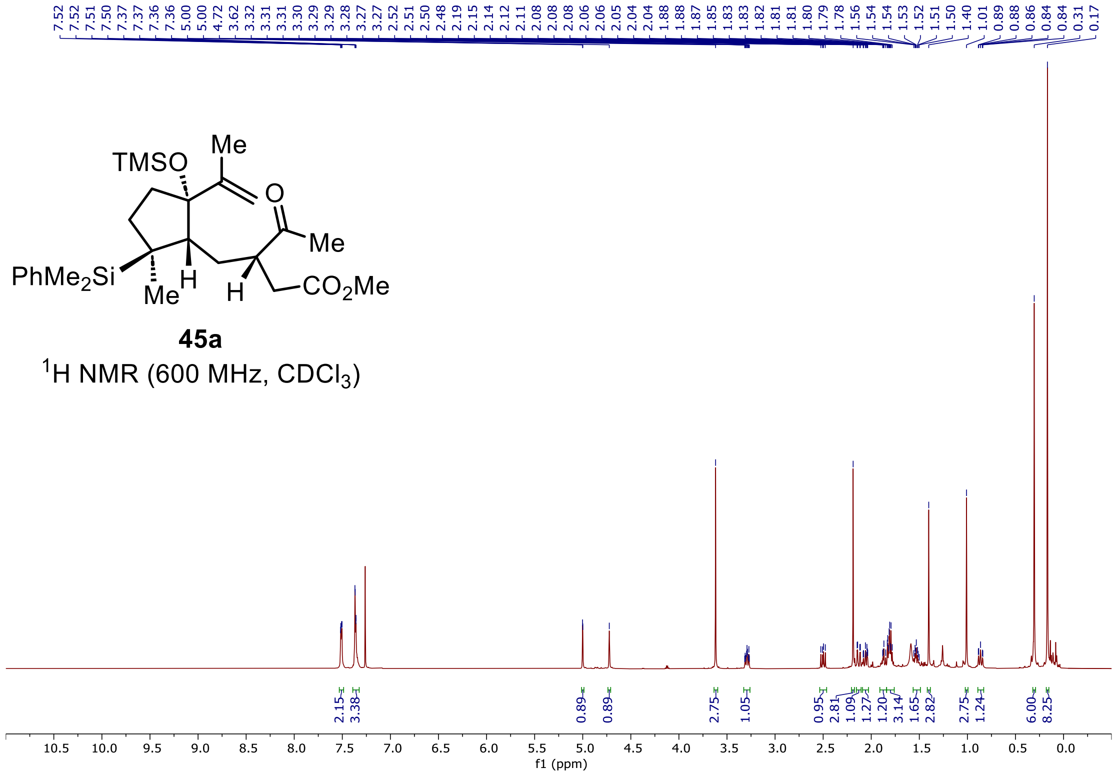

45a

^{1}H NMR (600 MHz, $CDCl_3$)

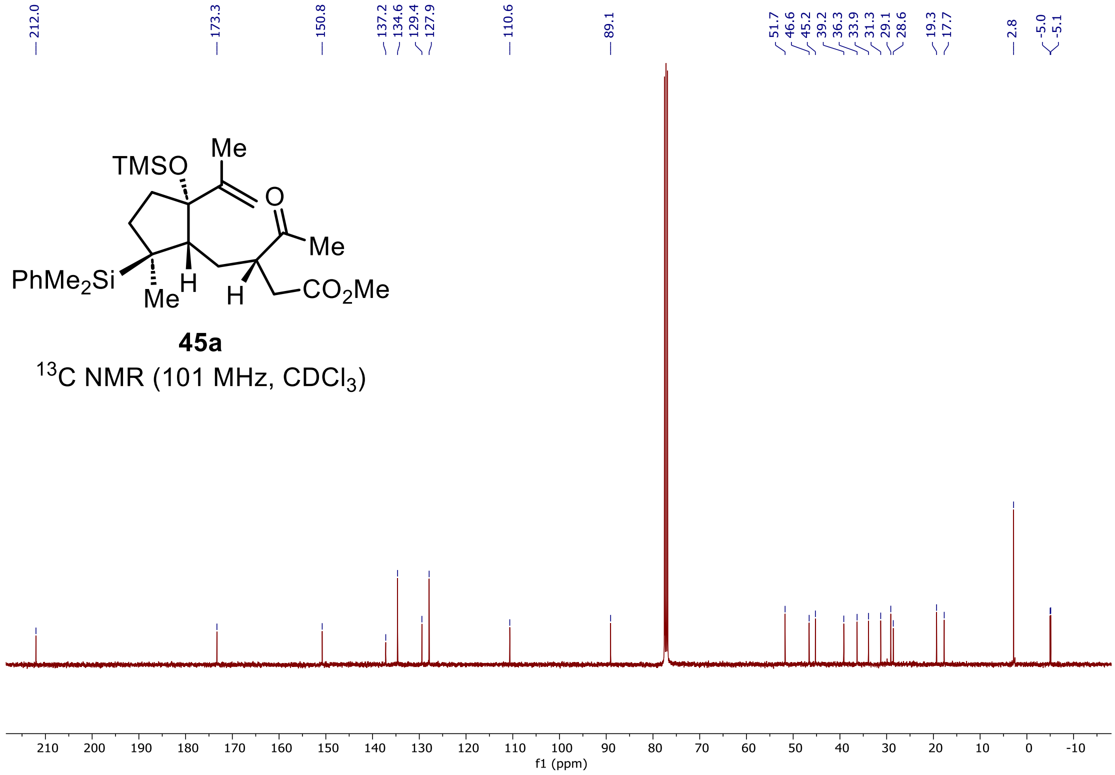

45a

^{13}C NMR (101 MHz, $CDCl_3$)

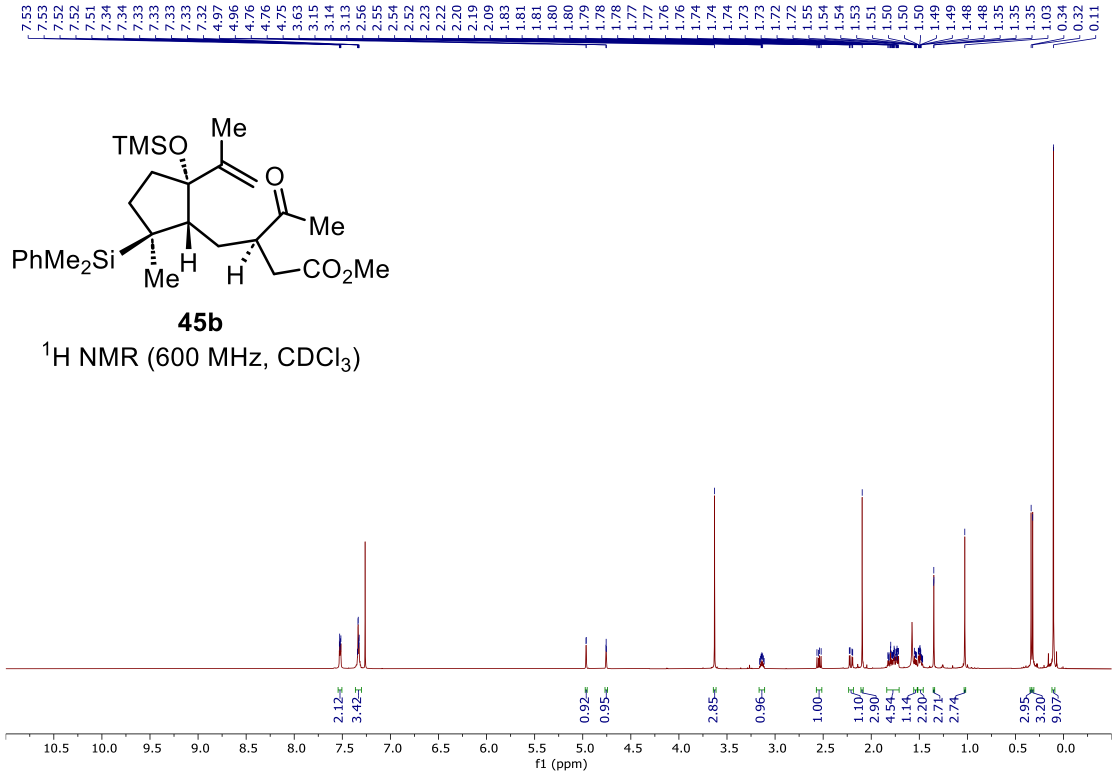

45b

^{1}H NMR (600 MHz, $CDCl_3$)

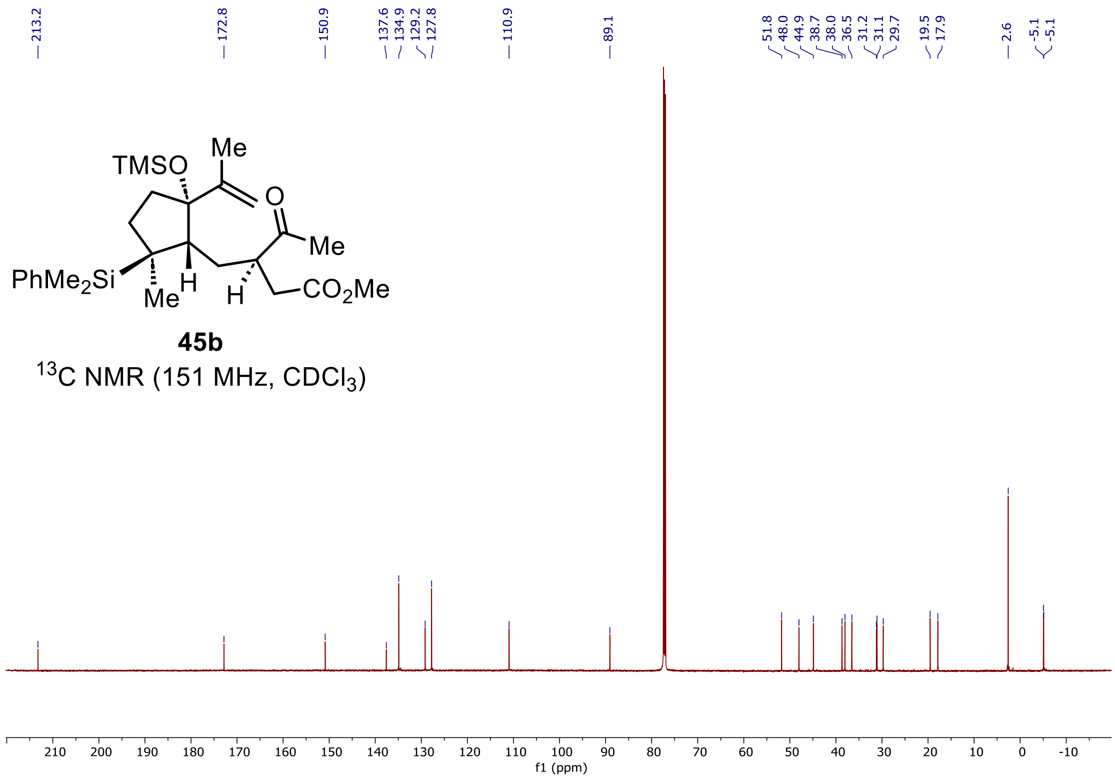

45b

^{13}C NMR (151 MHz, $CDCl_3$)

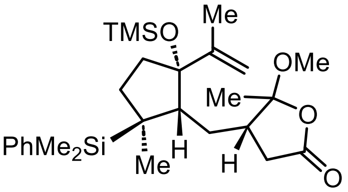

44a

^{1}H NMR (600 MHz, CDCl$_3$)

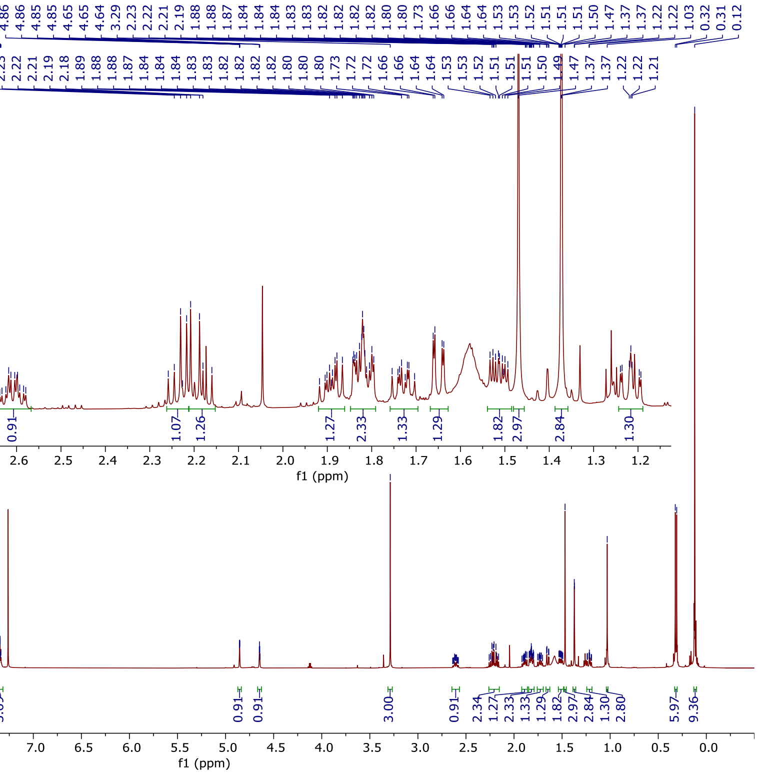

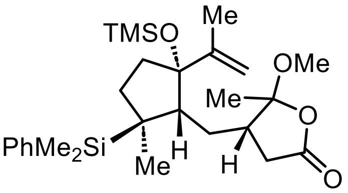

44a

^{13}C NMR (151 MHz, CDCl$_3$)

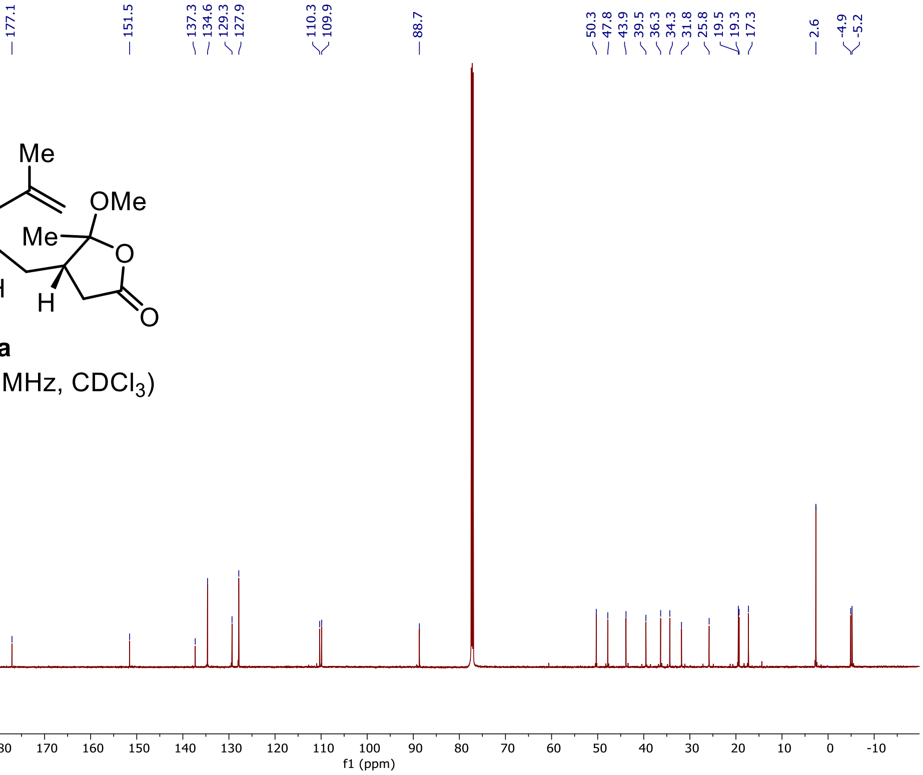

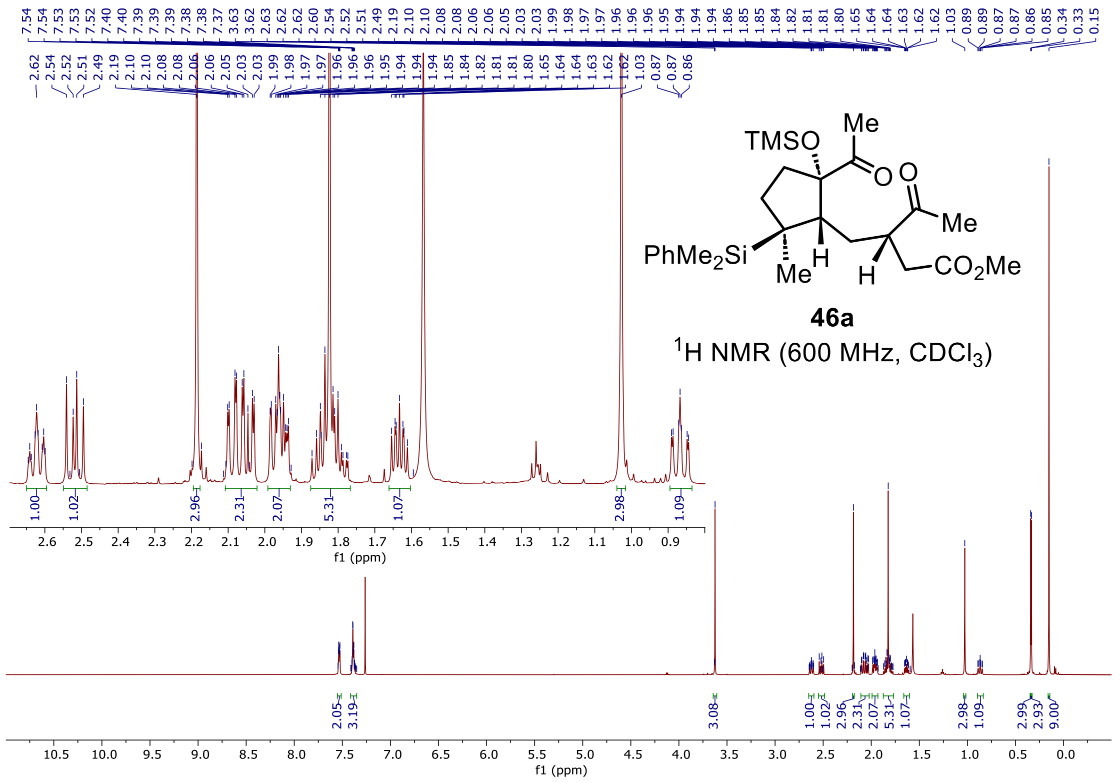

46a

^{1}H NMR (600 MHz, $CDCl_3$)

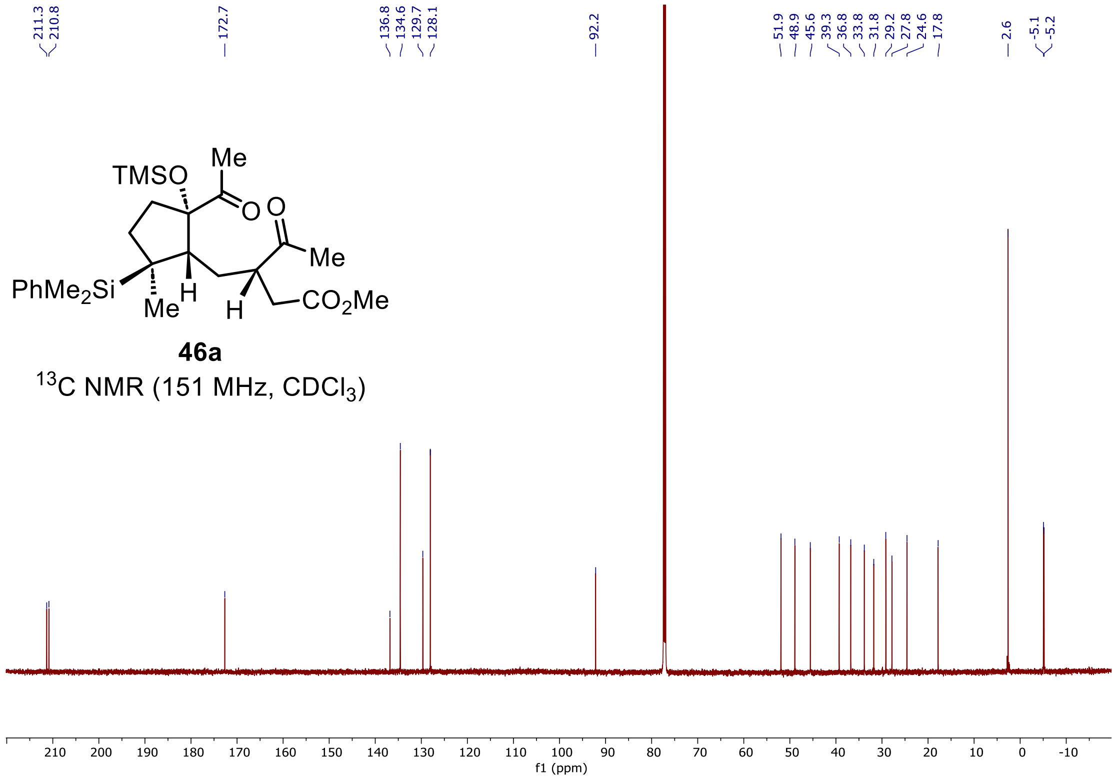

46a

^{13}C NMR (151 MHz, $CDCl_3$)

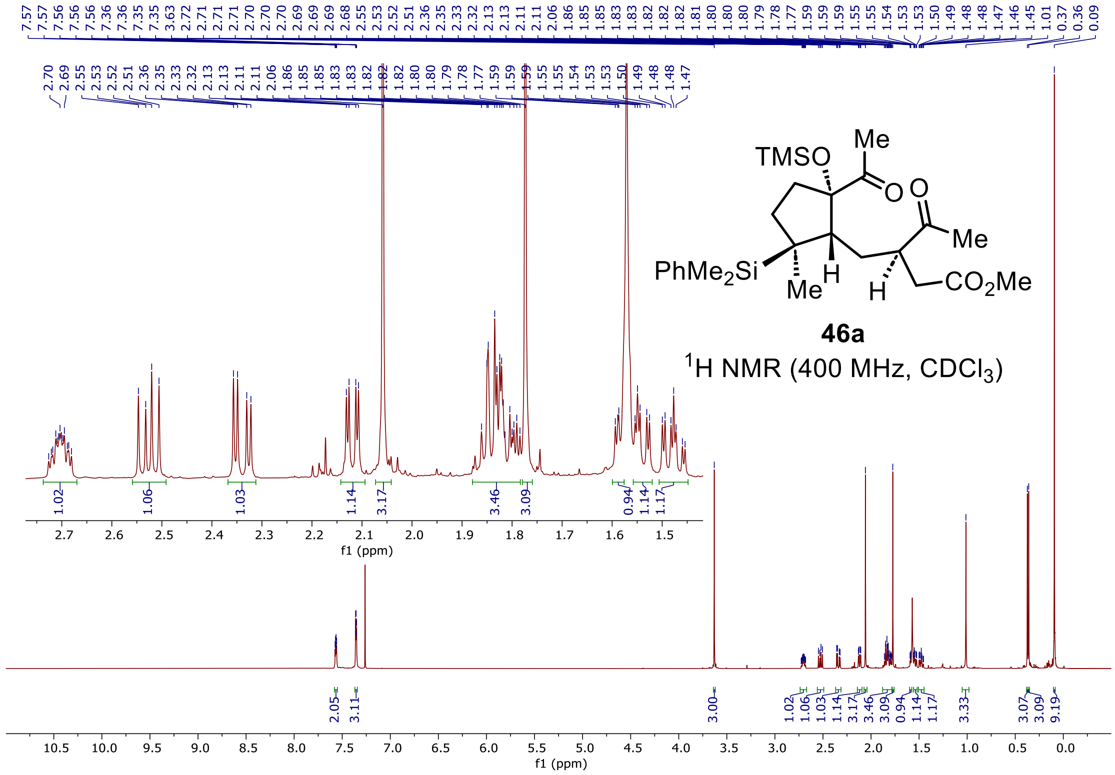

TMSO
Me
O
O
Me
PhMe2Si
Me
H
H
CO2Me
46a
1H NMR (400 MHz, CDCl3)
f1 (ppm)

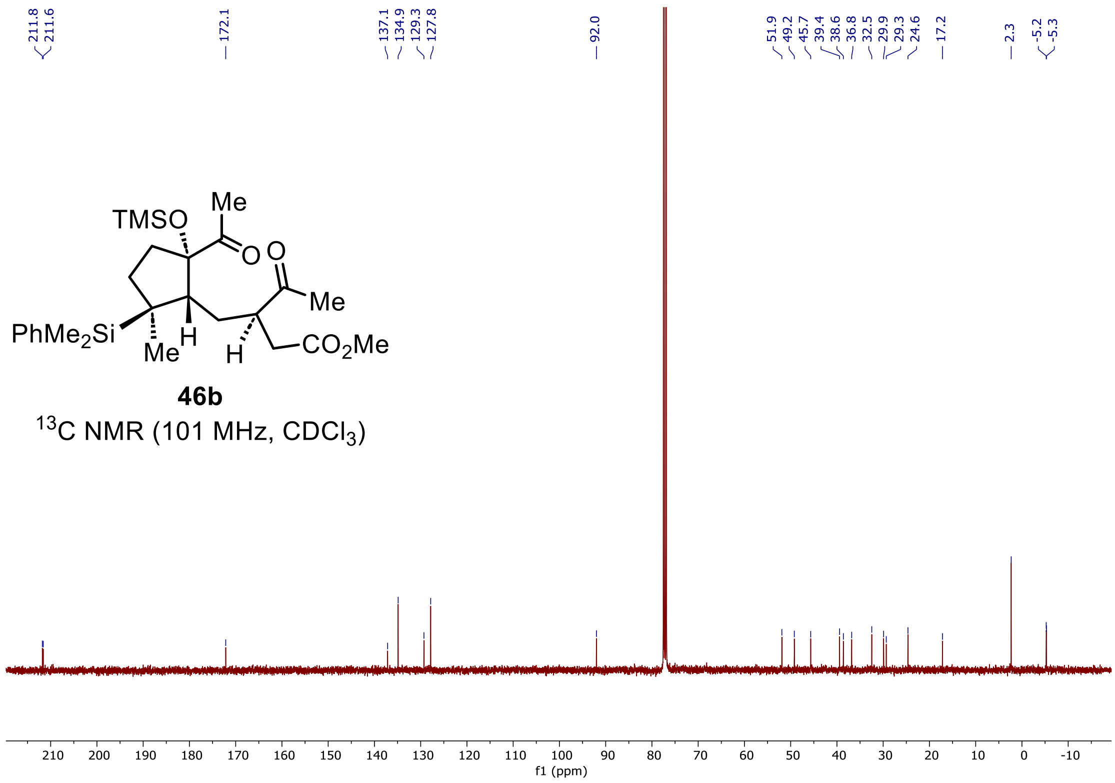

TMSO
Me
O
O
Me
PhMe2Si
Me
H
H
CO2Me
46b
13C NMR (101 MHz, CDCl3)
f1 (ppm)

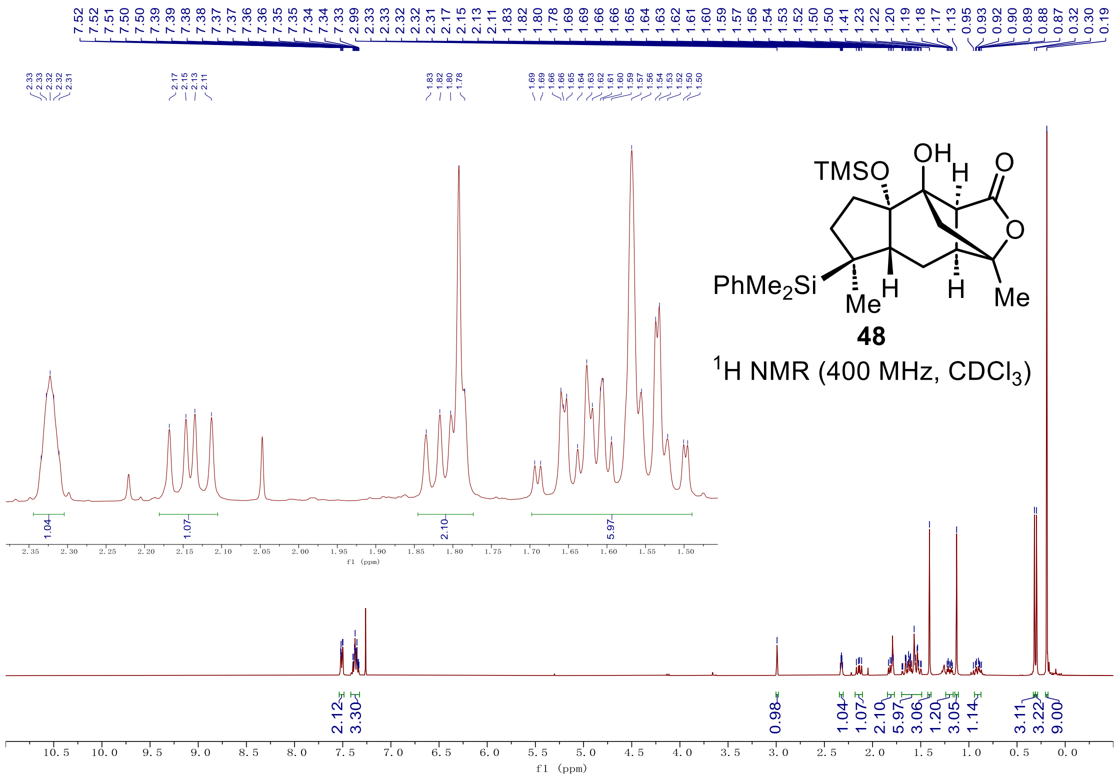
7.52 7.52 7.51 7.50 7.50 7.39 7.39 7.38 7.38 7.37 7.37 7.36 7.36 7.35 7.35 7.34 7.34 7.33 2.99 2.33 2.33 2.32 2.32 2.31 2.17 2.15 2.13 2.11 1.83 1.82 1.80 1.78 1.69 1.69 1.66 1.66 1.65 1.65 1.64 1.63 1.62 1.61 1.60 1.59 1.57 1.56 1.54 1.53 1.52 1.50 1.50 1.41 1.23 1.22 1.20 1.19 1.18 1.17 1.13 0.95 0.93 0.92 0.90 0.89 0.88 0.87 0.32 0.30 0.19
TMSO
OH
H
O
O
PhMe2Si
Me
H
H
Me
48
1H NMR (400 MHz, CDCl3)
1.04
1.07
2.10
5.97
2.12
3.30
0.98
1.04
1.07
2.10
5.97
3.06
1.20
3.05
1.14
3.11
3.22
9.00
f1 (ppm)

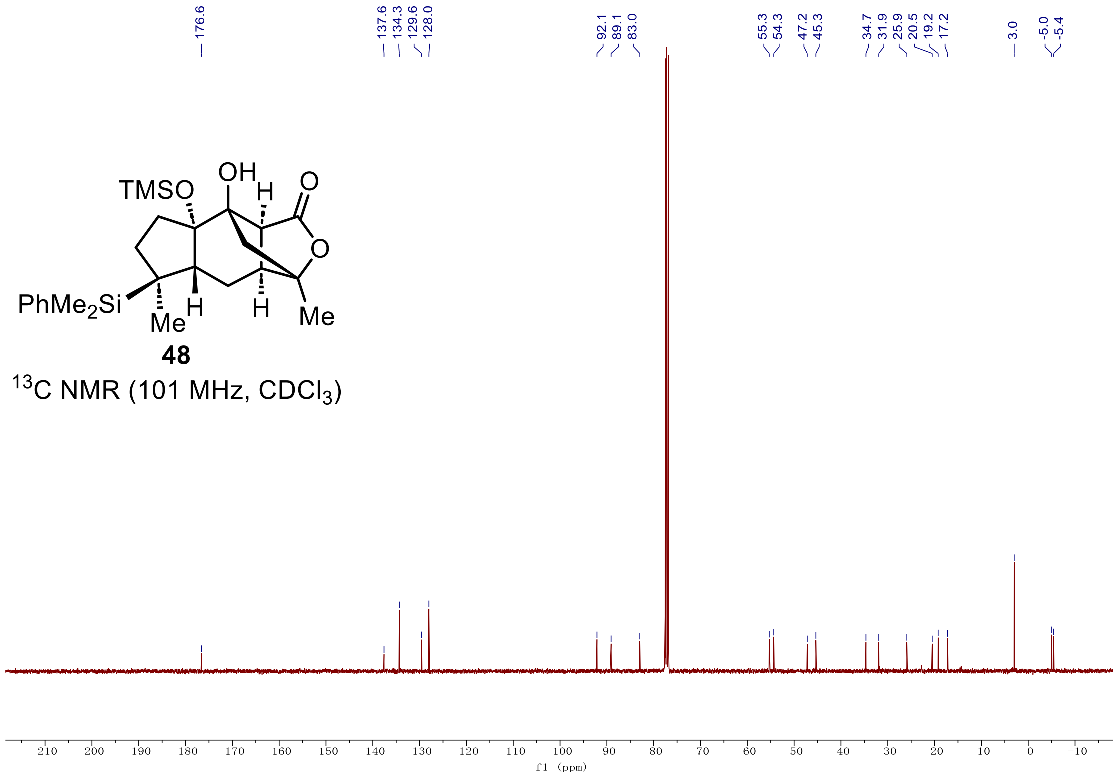
176.6
137.6 134.3 129.6 128.0
92.1 89.1 83.0
55.3 54.3 47.2 45.3
34.7 31.9 25.9 20.5 19.2 17.2
3.0
-5.0 -5.4
TMSO
OH
H
O
O
PhMe2Si
Me
H
H
Me
48
13C NMR (101 MHz, CDCl3)
f1 (ppm)

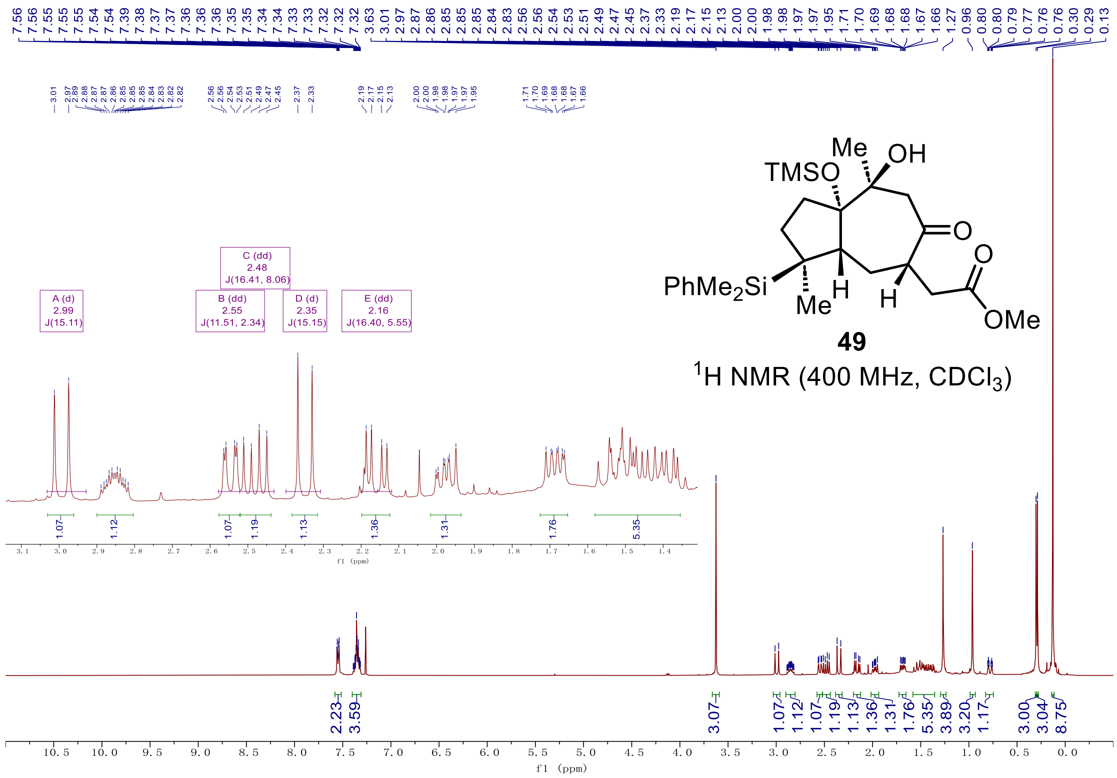

49

^{1}H NMR (400 MHz, $CDCl_3$)

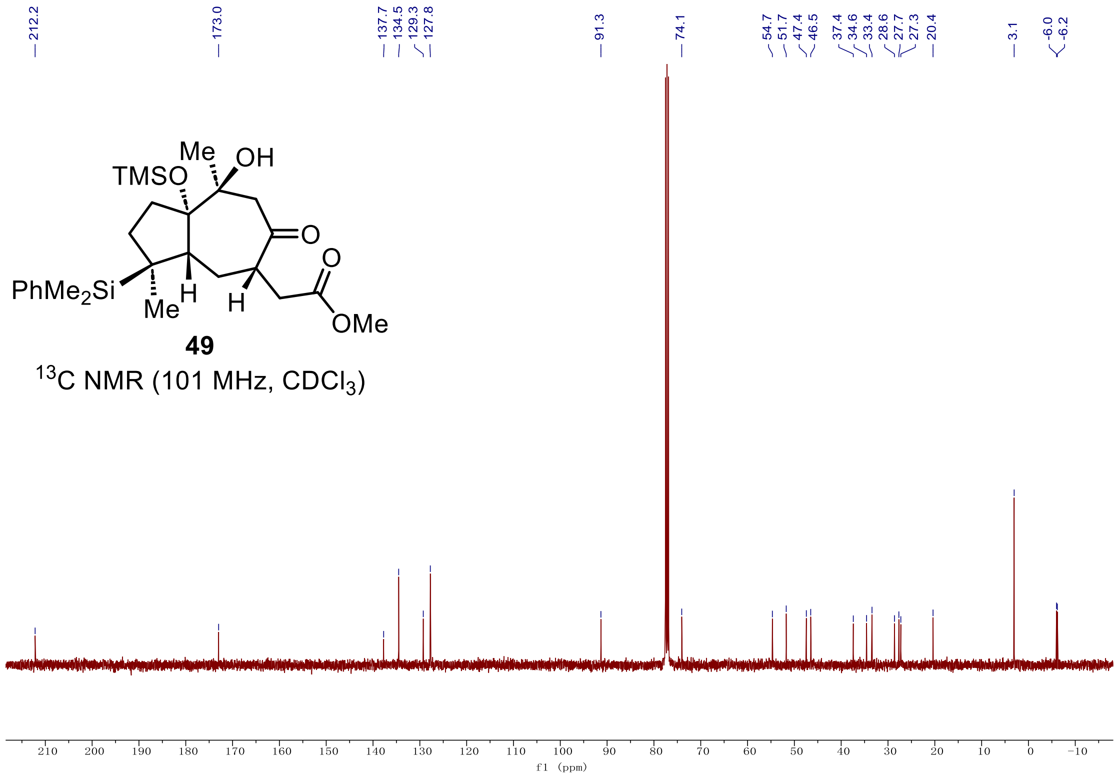

49

^{13}C NMR (101 MHz, $CDCl_3$)

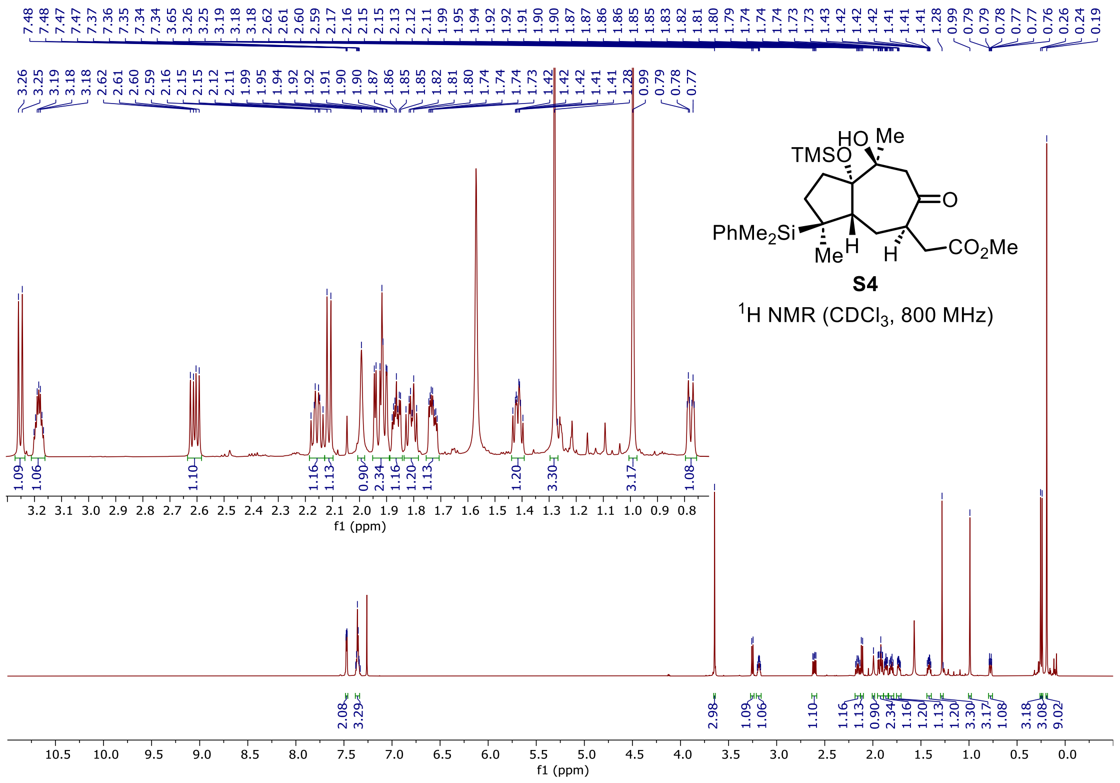
TMSO
HO
Me
PhMe2Si
Me
H
H
CO2Me
S4
1H NMR (CDCl3, 800 MHz)
f1 (ppm)

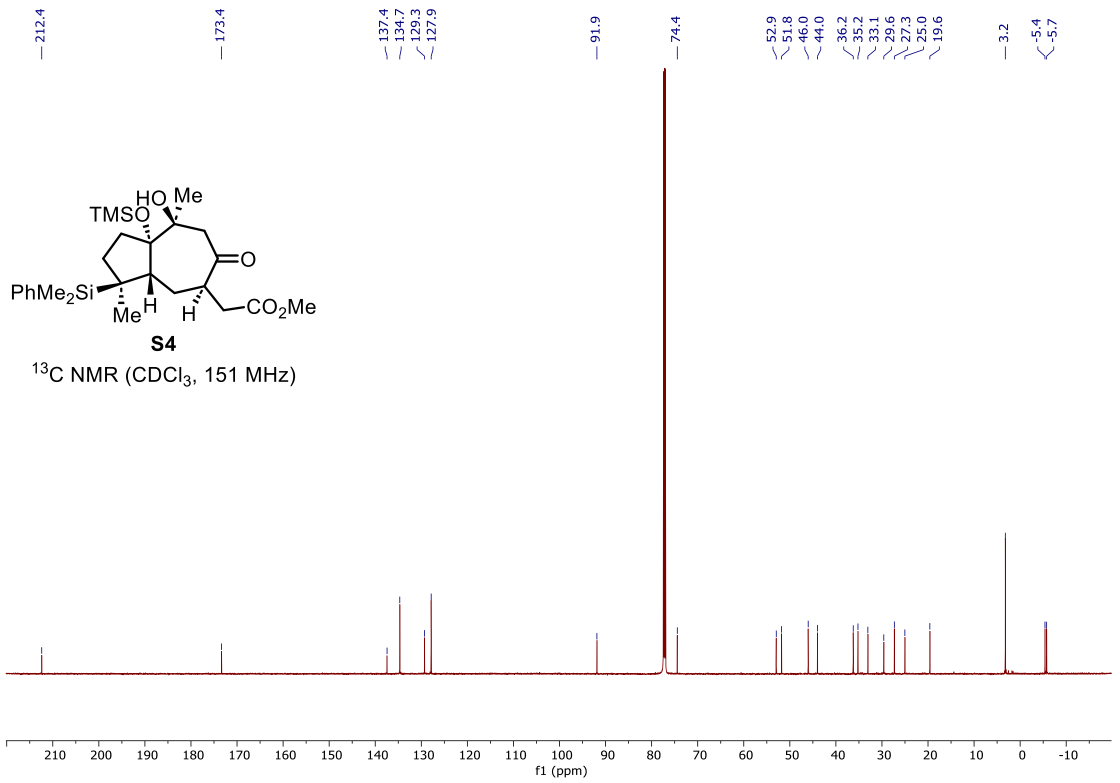
212.4
173.4
137.4
134.7
129.3
127.9
91.9
74.4
52.9
51.8
46.0
44.0
36.2
35.2
33.1
29.6
27.3
25.0
19.6
3.2
-5.4
-5.7
TMSO
HO
Me
PhMe2Si
Me
H
H
CO2Me
S4
13C NMR (CDCl3, 151 MHz)
f1 (ppm)

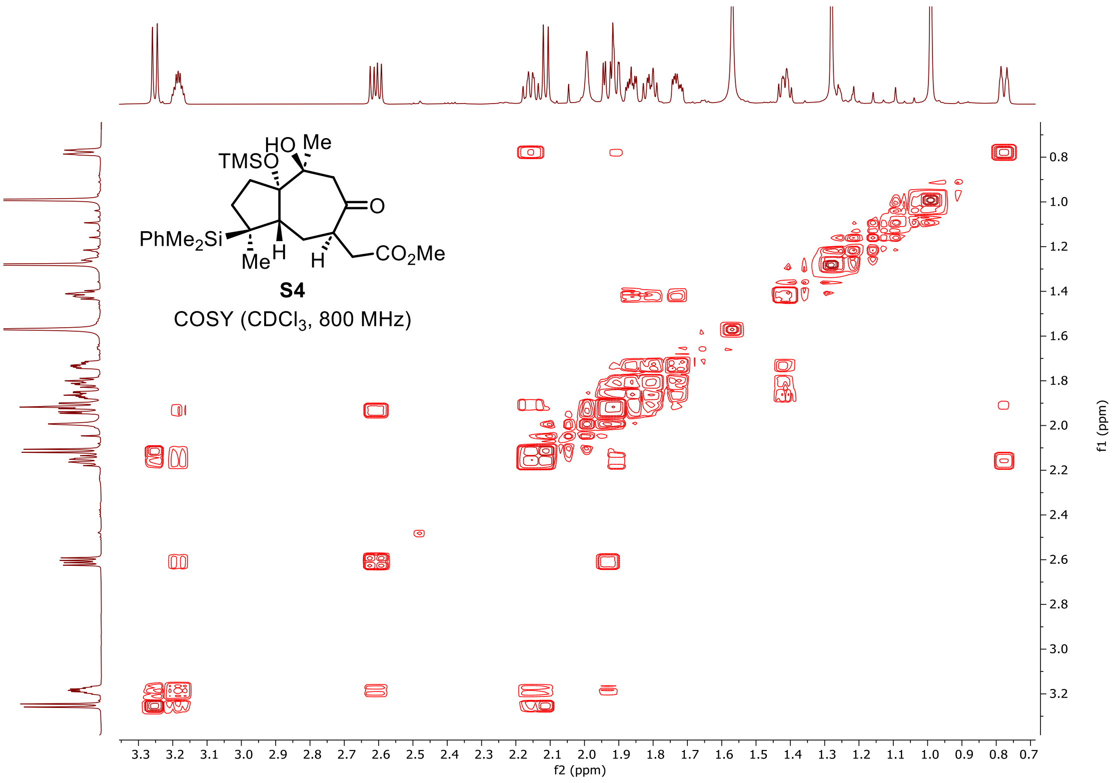

S4

COSY ($CDCl_3$, 800 MHz)

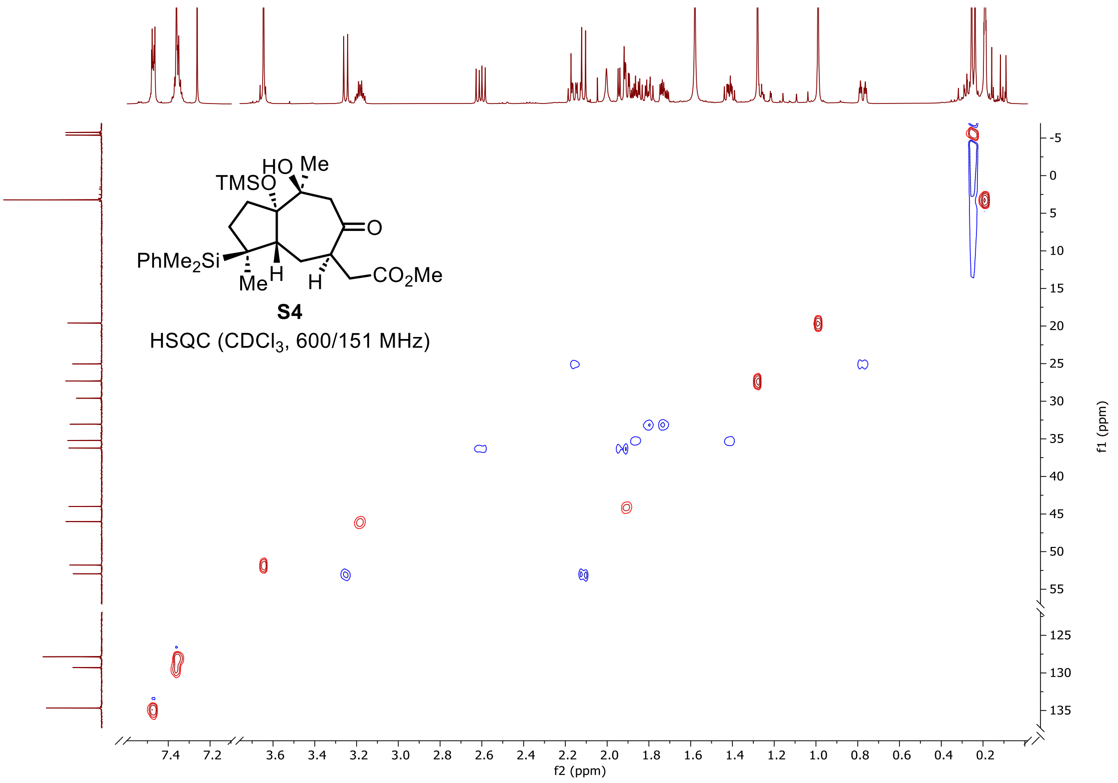

S4

HSQC ($CDCl_3$, 600/151 MHz)

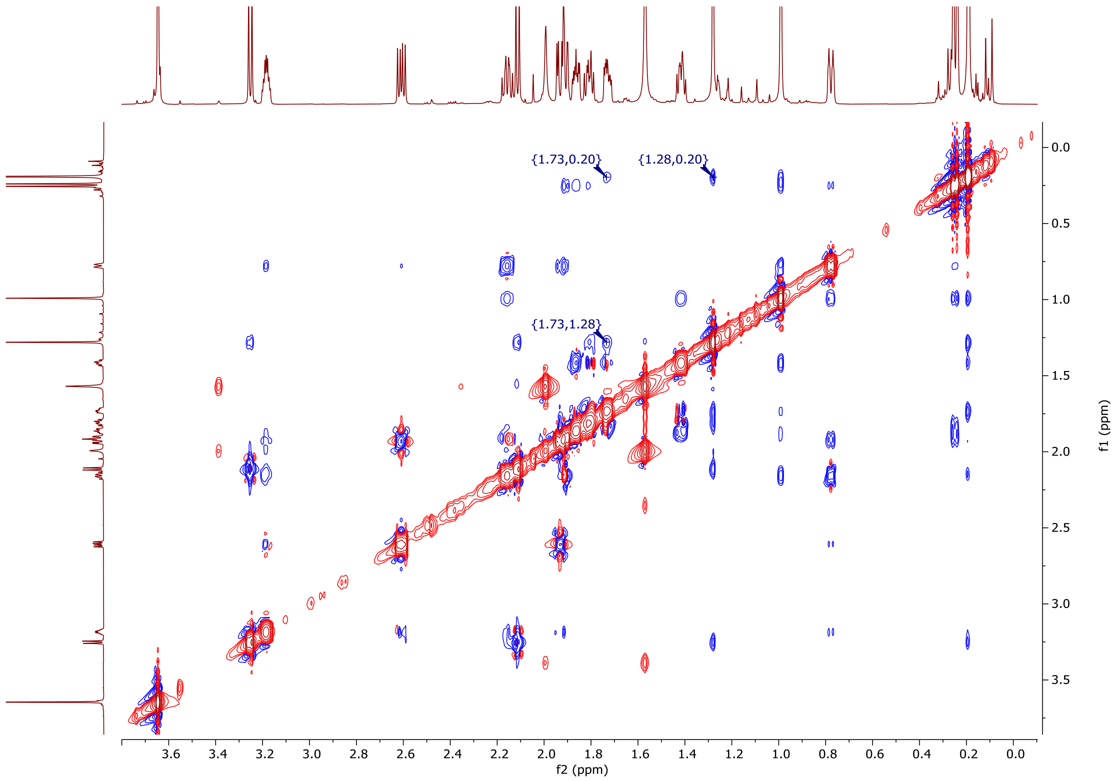

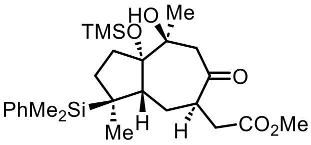

S4

NOESY ($CDCl_3$, 800 MHz)

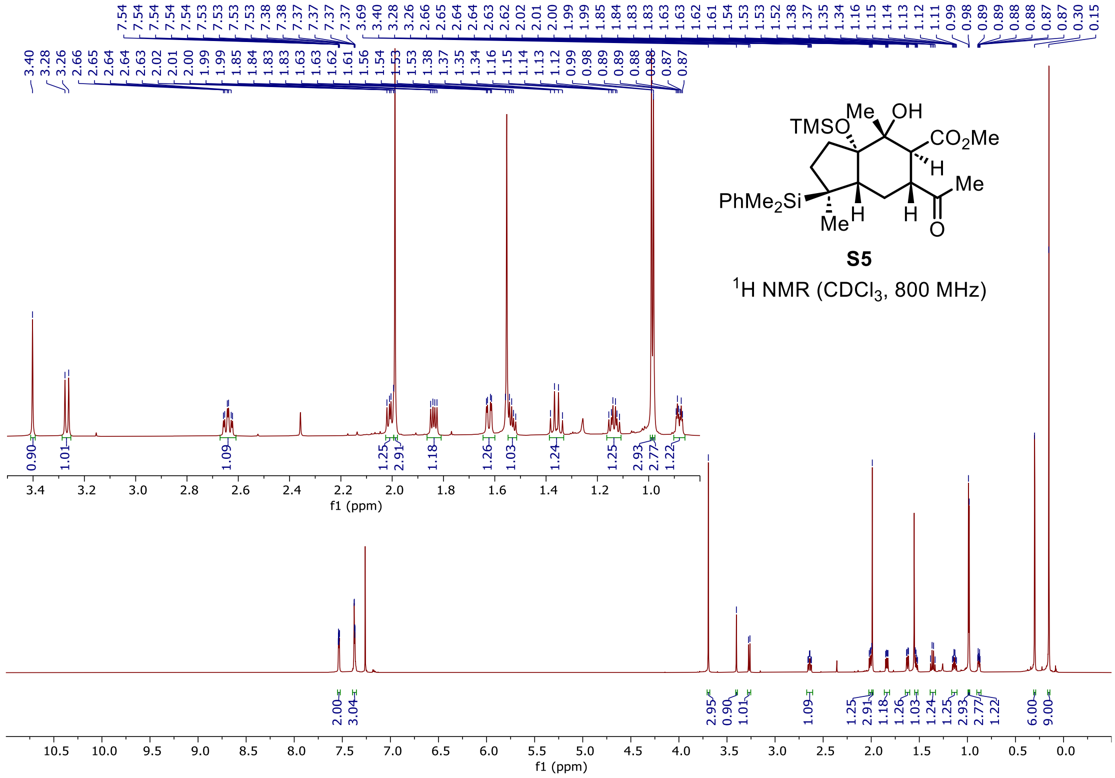
7.54 7.54 7.54 7.54 7.54 7.53 7.53 7.53 7.38 7.38 7.37 7.37 7.37 7.37 3.69 3.40 3.28 3.26 2.66 2.65 2.64 2.63 2.62 2.02 2.01 2.00 1.99 1.99 1.85 1.84 1.83 1.83 1.63 1.63 1.62 1.61 1.54 1.53 1.53 1.52 1.38 1.37 1.35 1.34 1.16 1.15 1.14 1.13 1.12 1.11 0.99 0.98 0.89 0.88 0.88 0.88 0.87 0.87 0.30 0.15
TMSO
Me
OH
CO2Me
H
Me
PhMe2Si
Me
H
H
O
S5
1H NMR (CDCl3, 800 MHz)
f1 (ppm)
f1 (ppm)

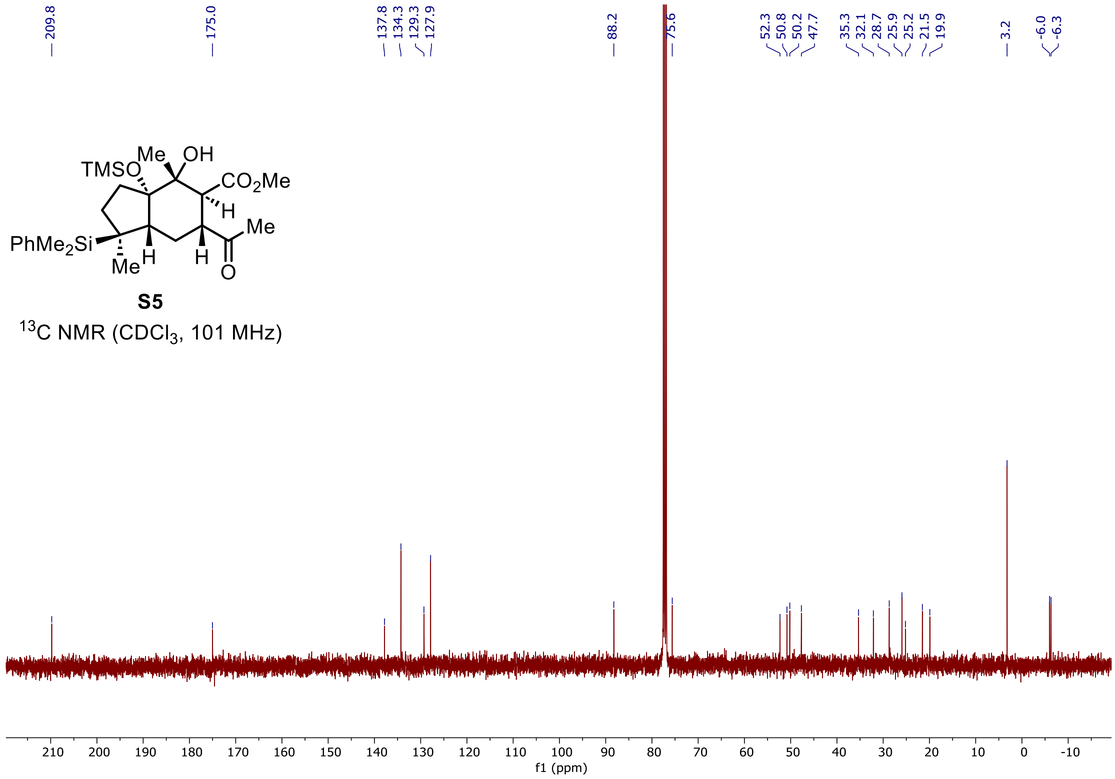
209.8
175.0
137.8
134.3
129.3
127.9
88.2
75.6
52.3
50.8
50.2
47.7
35.3
32.1
28.7
25.9
25.2
21.5
19.9
3.2
-6.0
-6.3
TMSO
Me
OH
CO2Me
H
Me
PhMe2Si
Me
H
H
O
S5
13C NMR (CDCl3, 101 MHz)
f1 (ppm)

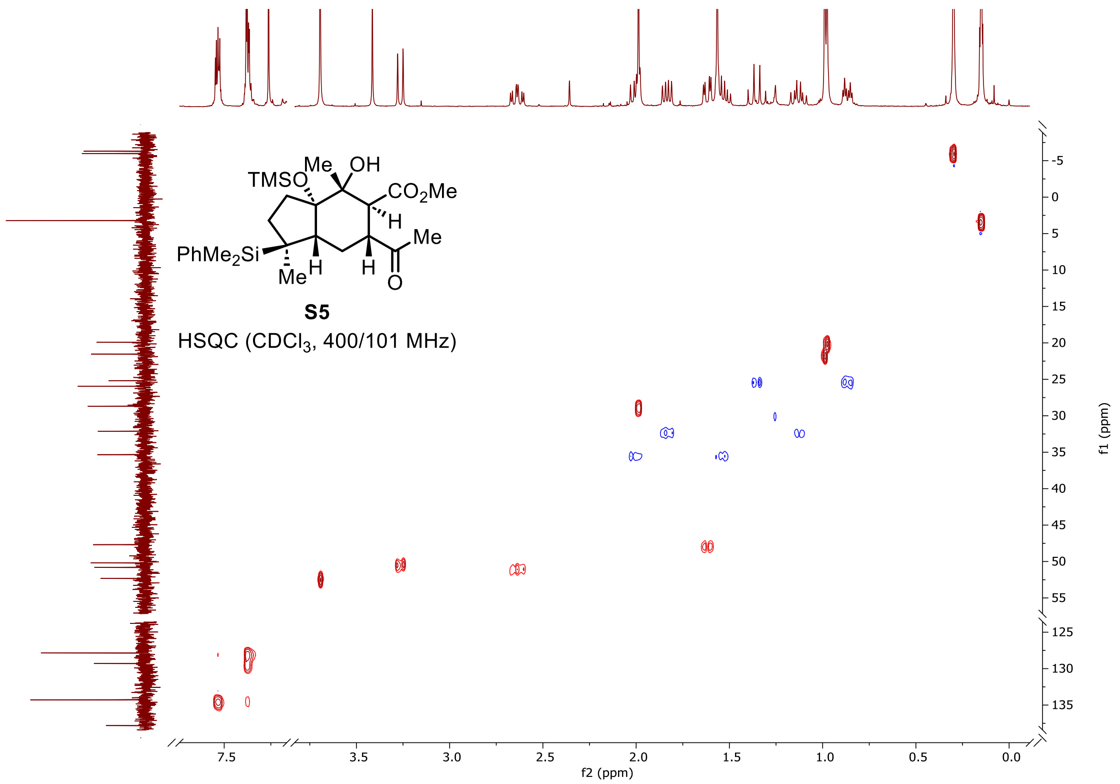

TMSO
Me
OH
CO2Me
H
Me
PhMe2Si
Me
H
H
O
S5
HSQC (CDCl3, 400/101 MHz)
f2 (ppm)
f1 (ppm)

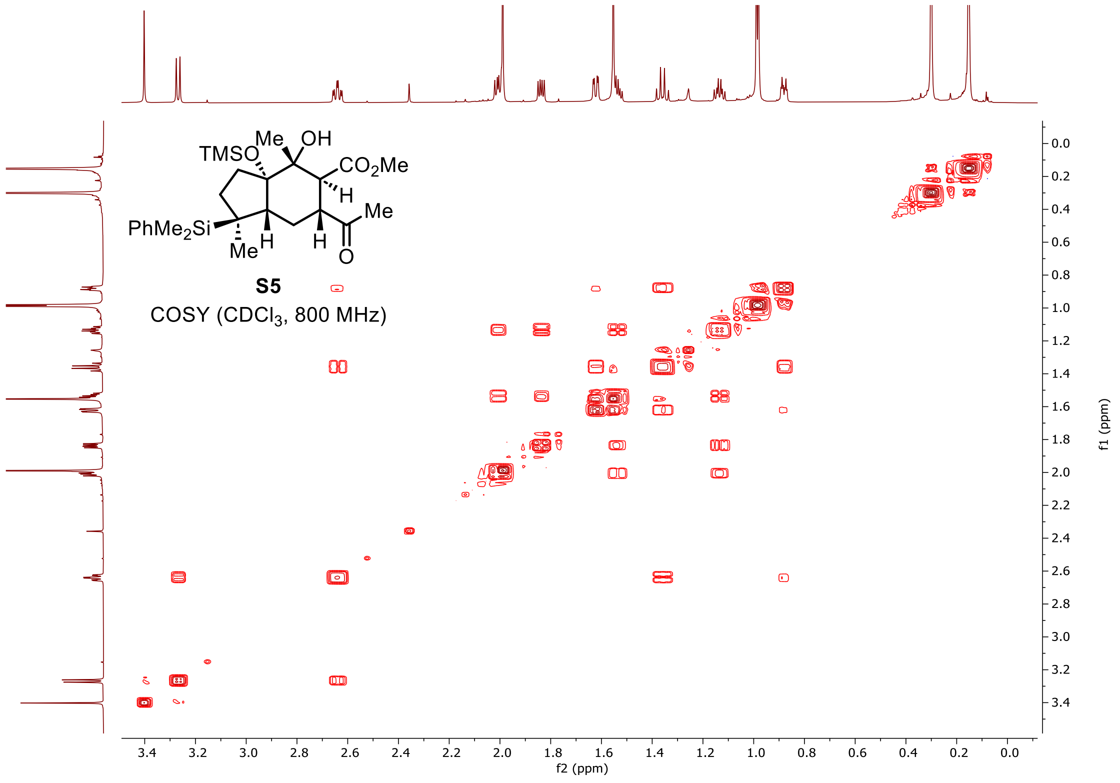

TMSO
Me
OH
CO2Me
H
Me
PhMe2Si
Me
H
H
O
S5
COSY (CDCl3, 800 MHz)
f2 (ppm)
f1 (ppm)

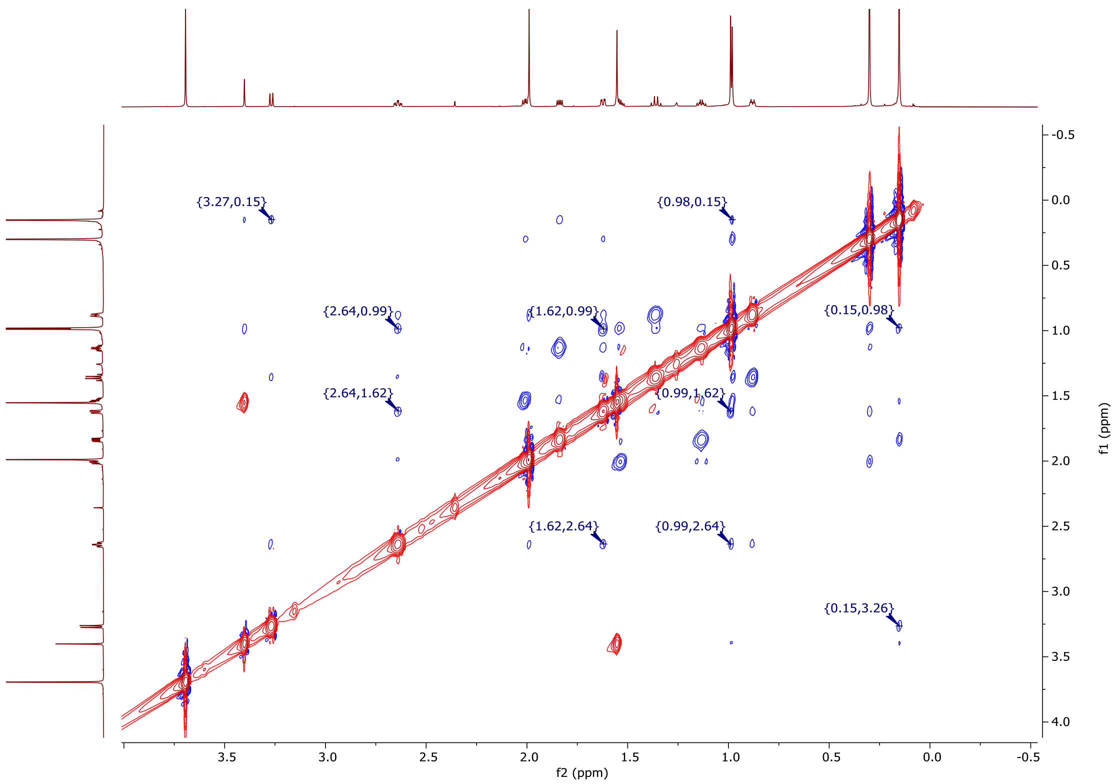

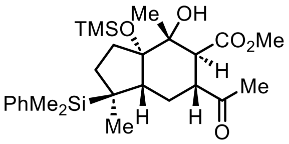

S5

NOESY ($CDCl_3$, 800 MHz)

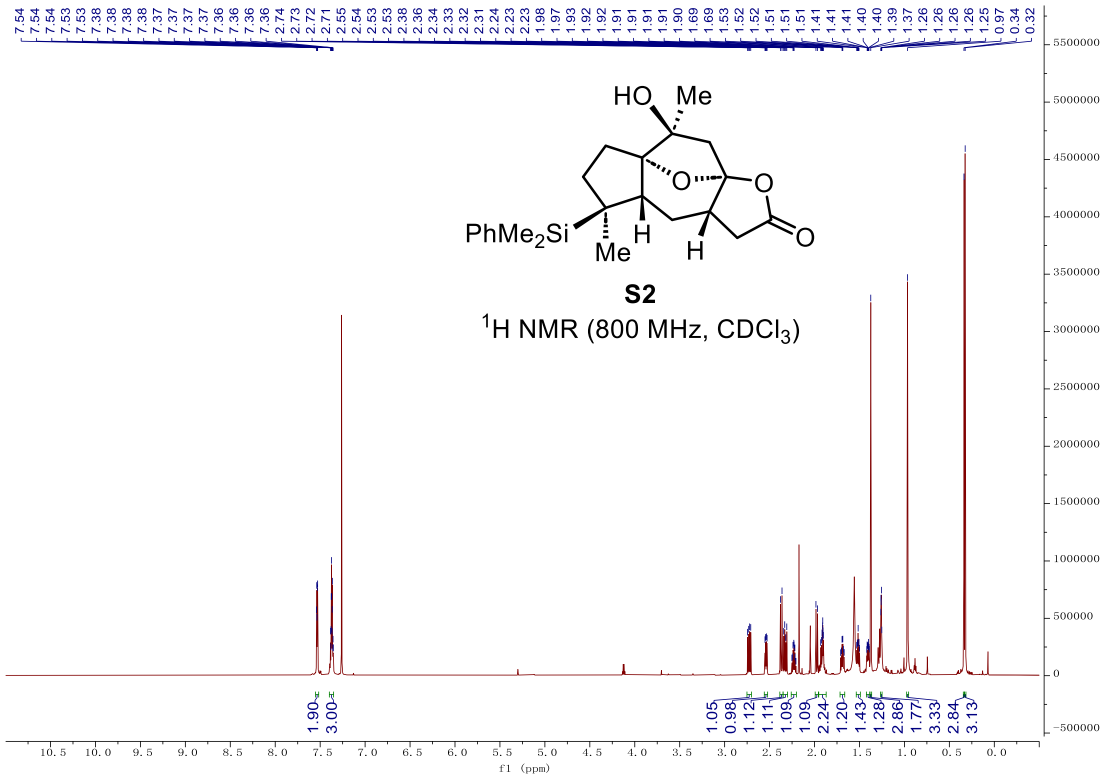
HO
Me
PhMe2Si
Me
H
H
O
O
O
S2
1H NMR (800 MHz, CDCl3)
f1 (ppm)

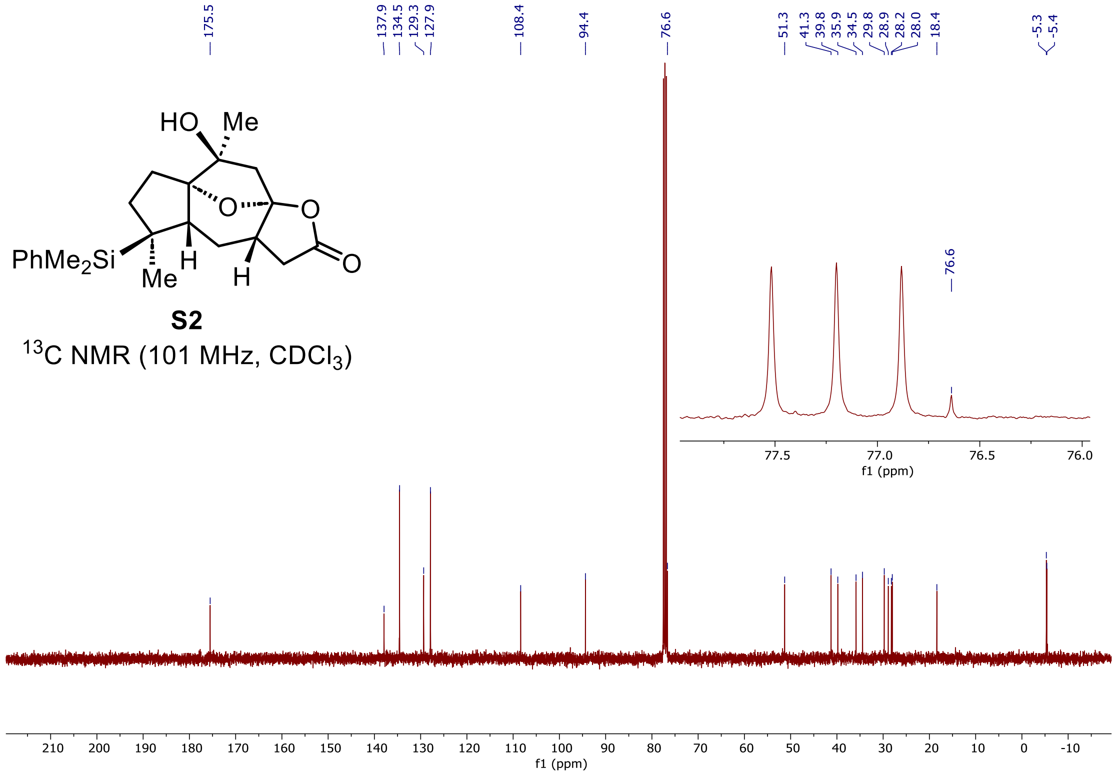
175.5
137.9
134.5
129.3
127.9
108.4
94.4
76.6
51.3
41.3
39.8
35.9
34.5
29.8
28.9
28.2
28.0
18.4
-5.3
-5.4
HO
Me
PhMe2Si
Me
H
H
O
O
O
S2
13C NMR (101 MHz, CDCl3)
f1 (ppm)

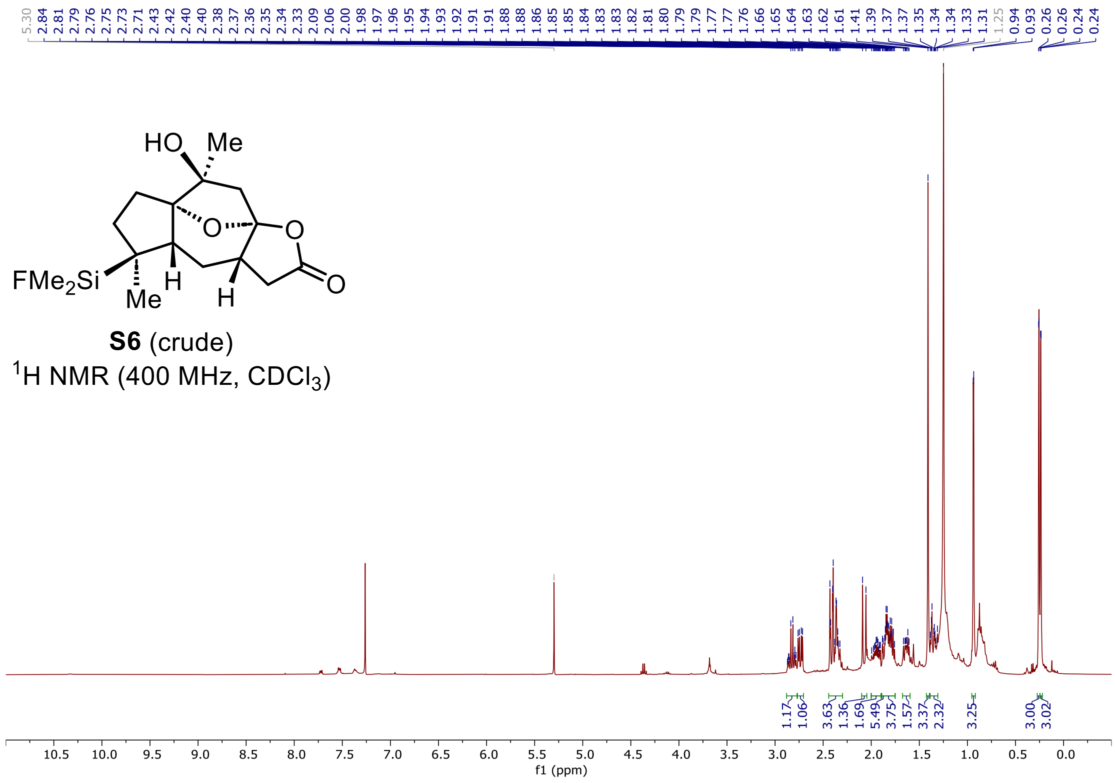
5.30
2.84 2.81 2.79 2.76 2.75 2.73 2.71 2.43 2.42 2.40 2.40 2.38 2.37 2.36 2.35 2.34 2.33 2.09 2.06 2.00 1.98 1.97 1.96 1.95 1.94 1.93 1.92 1.91 1.91 1.88 1.88 1.86 1.85 1.85 1.84 1.83 1.83 1.82 1.81 1.80 1.79 1.79 1.77 1.77 1.76 1.66 1.65 1.64 1.63 1.62 1.61 1.41 1.39 1.37 1.37 1.35 1.34 1.34 1.33 1.31
1.25
0.94 0.93 0.26 0.26 0.24 0.24
HO
Me
O
O
O
FMe2Si
Me
H
H
S6 (crude)
1H NMR (400 MHz, CDCl3)
1.17 1.06 3.63 1.36 1.69 5.49 3.75 1.57 3.37 2.32 3.25 3.00 3.02
10.5 10.0 9.5 9.0 8.5 8.0 7.5 7.0 6.5 6.0 5.5 5.0 4.5 4.0 3.5 3.0 2.5 2.0 1.5 1.0 0.5 0.0
f1 (ppm)

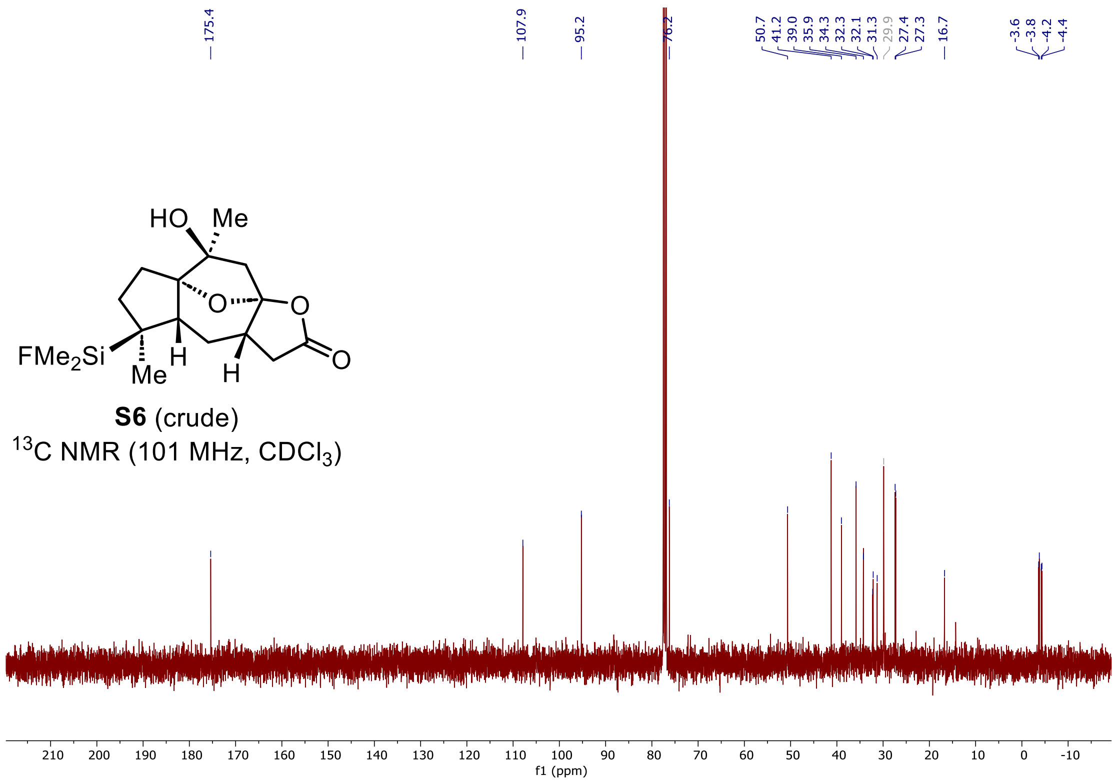
175.4
107.9
95.2
76.2
50.7 41.2 39.0 35.9 34.3 32.3 32.1 31.3
29.9
27.4 27.3 16.7
-3.6 -3.8 -4.2 -4.4
HO
Me
O
O
O
FMe2Si
Me
H
H
S6 (crude)
13C NMR (101 MHz, CDCl3)
210 200 190 180 170 160 150 140 130 120 110 100 90 80 70 60 50 40 30 20 10 0 -10
f1 (ppm)

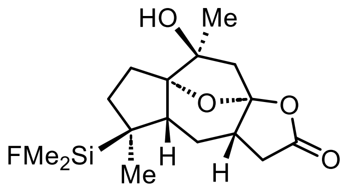

S6 (crude)
^{19}F NMR (376 MHz, $CDCl_3$)

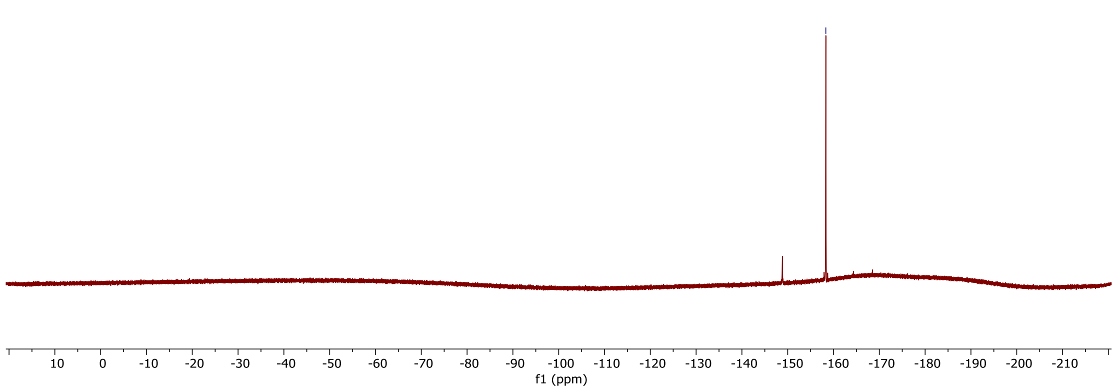

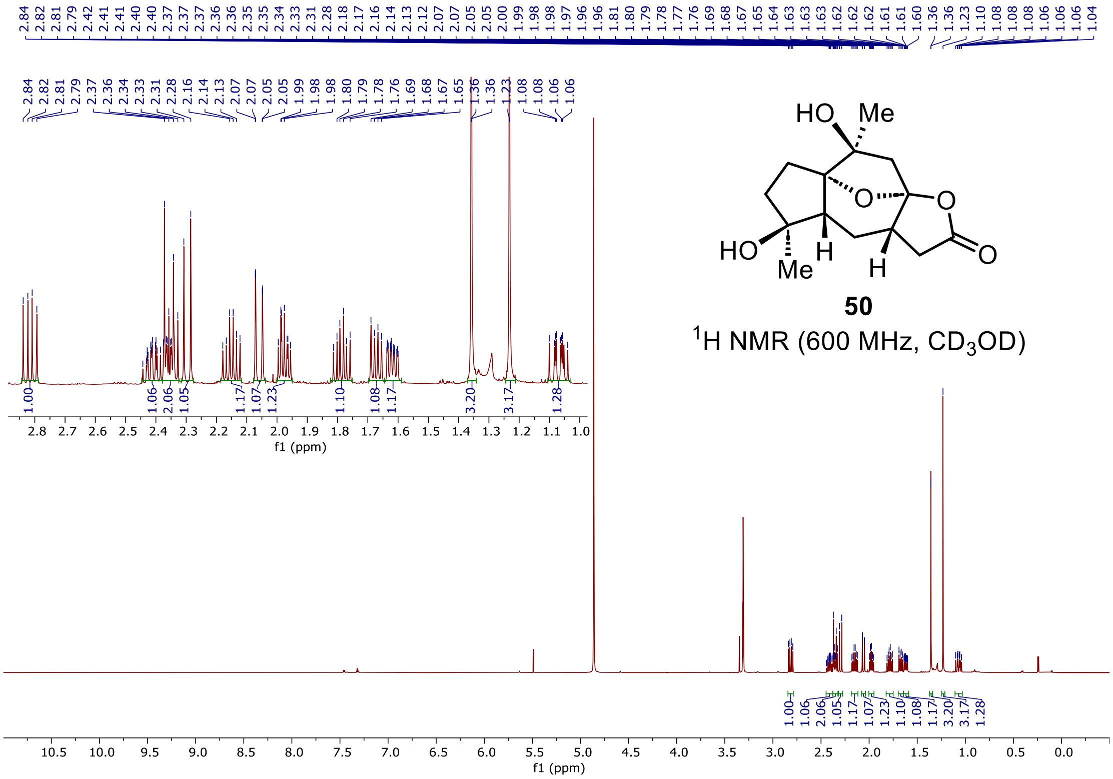

HO Me
O
O
O
HO H
Me H
50
^{1}H NMR (600 MHz, CD$_3$OD)
2.84 2.82 2.81 2.79 2.37 2.36 2.34 2.33 2.31 2.28 2.16 2.14 2.13 2.07 2.07 2.05 2.05 1.99 1.98 1.98 1.80 1.79 1.78 1.76 1.69 1.68 1.67 1.65 1.36 1.36 1.23 1.08 1.08 1.06 1.06
1.00 1.06 2.06 1.05 1.17 1.07 1.23 1.10 1.08 1.17 3.20 3.17 1.28
f1 (ppm)

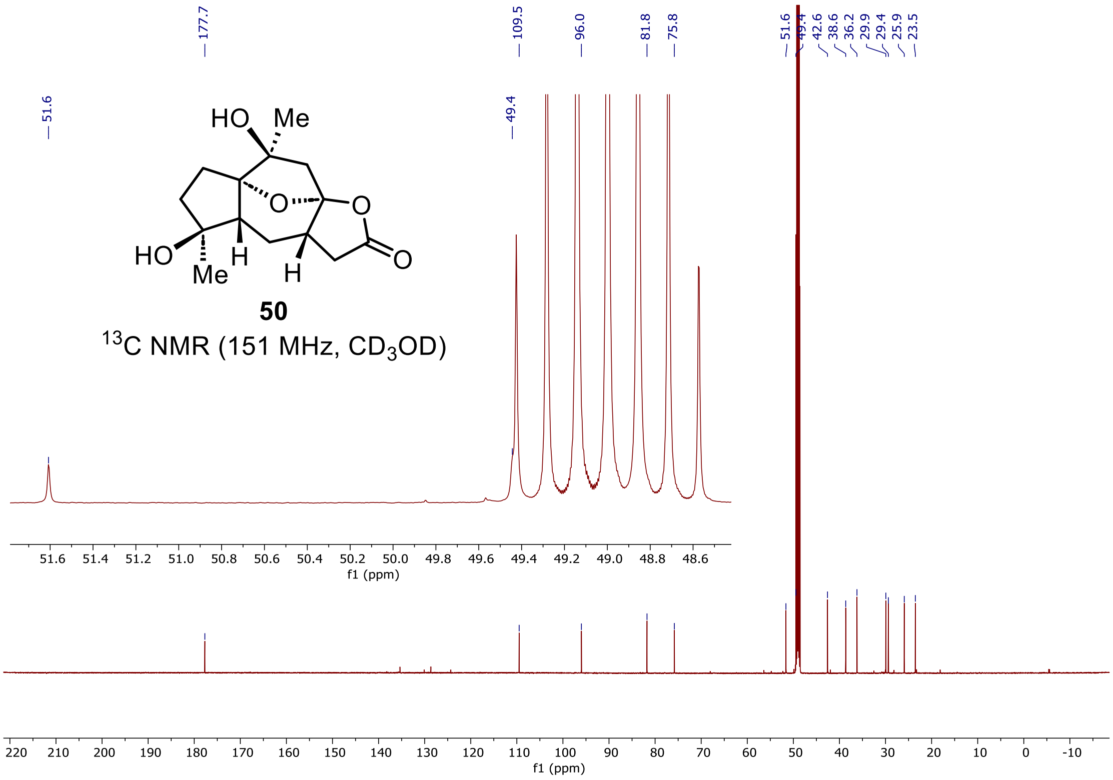

177.7
109.5
96.0
81.8
75.8
51.6
49.4
42.6
38.6
36.2
29.9
29.4
25.9
23.5
HO Me
O
O
O
HO H
Me H
50
^{13}C NMR (151 MHz, CD$_3$OD)
f1 (ppm)

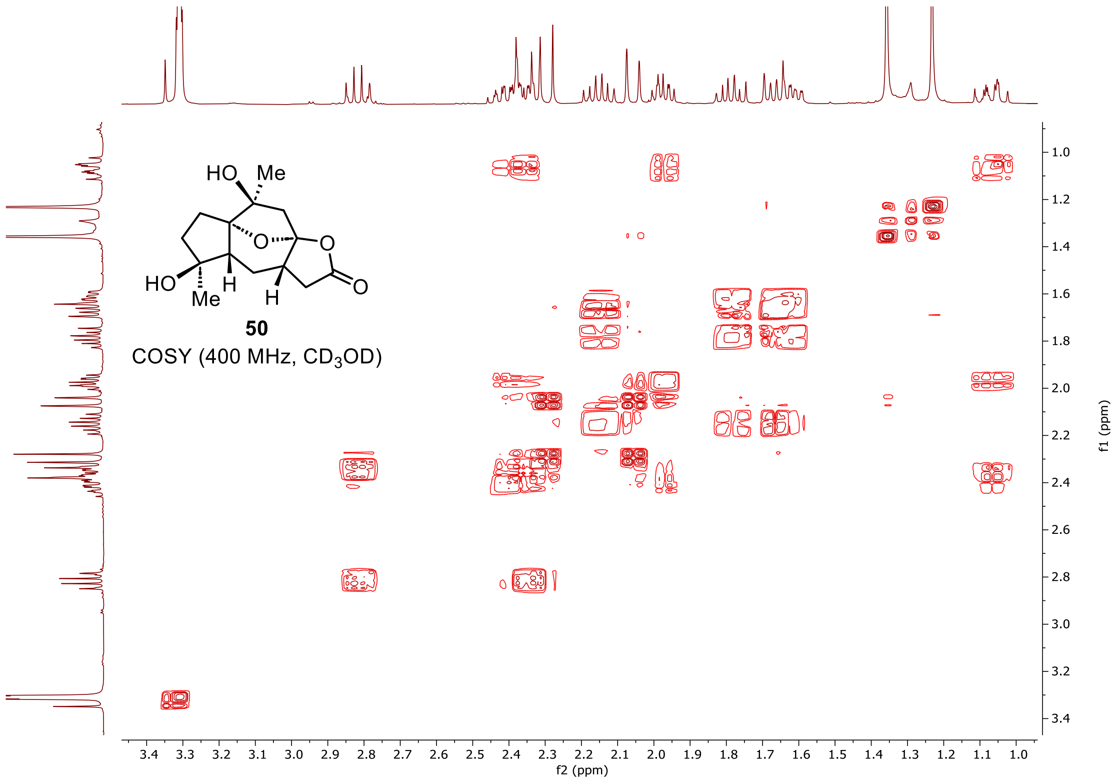
HO
Me
O
O
O
HO
Me
H
H
50
COSY (400 MHz, CD_3OD)
f2 (ppm)
f1 (ppm)

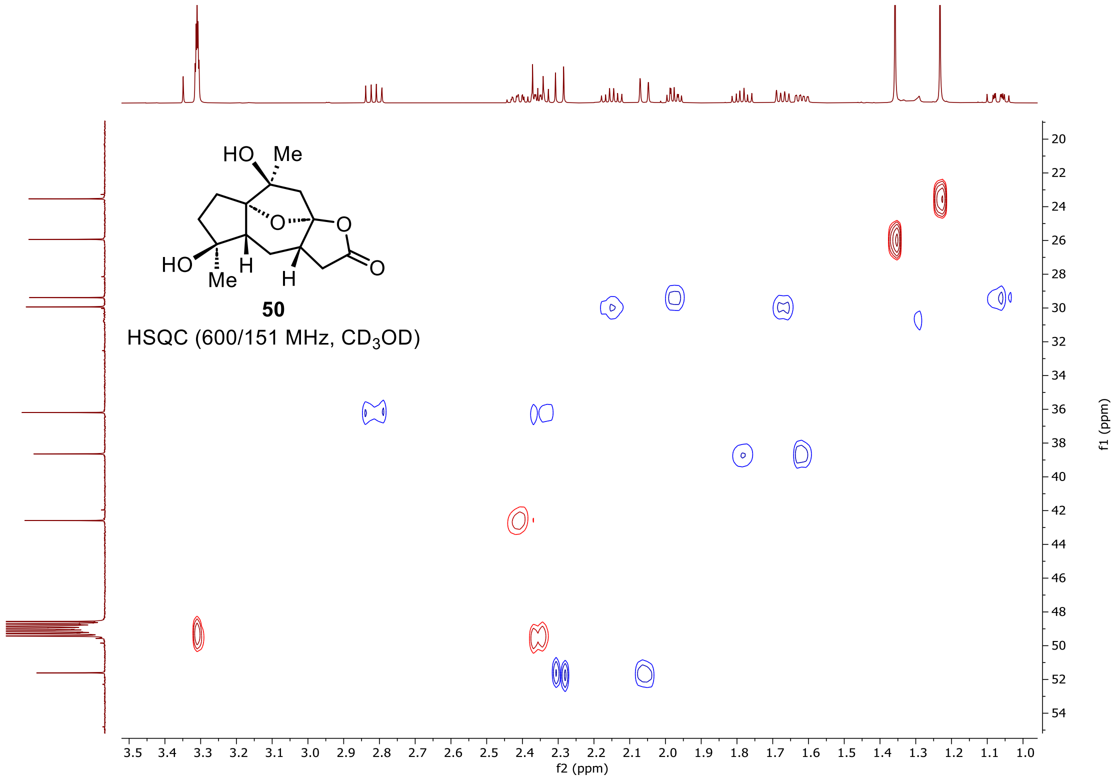
HO
Me
O
O
O
HO
Me
H
H
50
HSQC (600/151 MHz, CD_3OD)
f2 (ppm)
f1 (ppm)

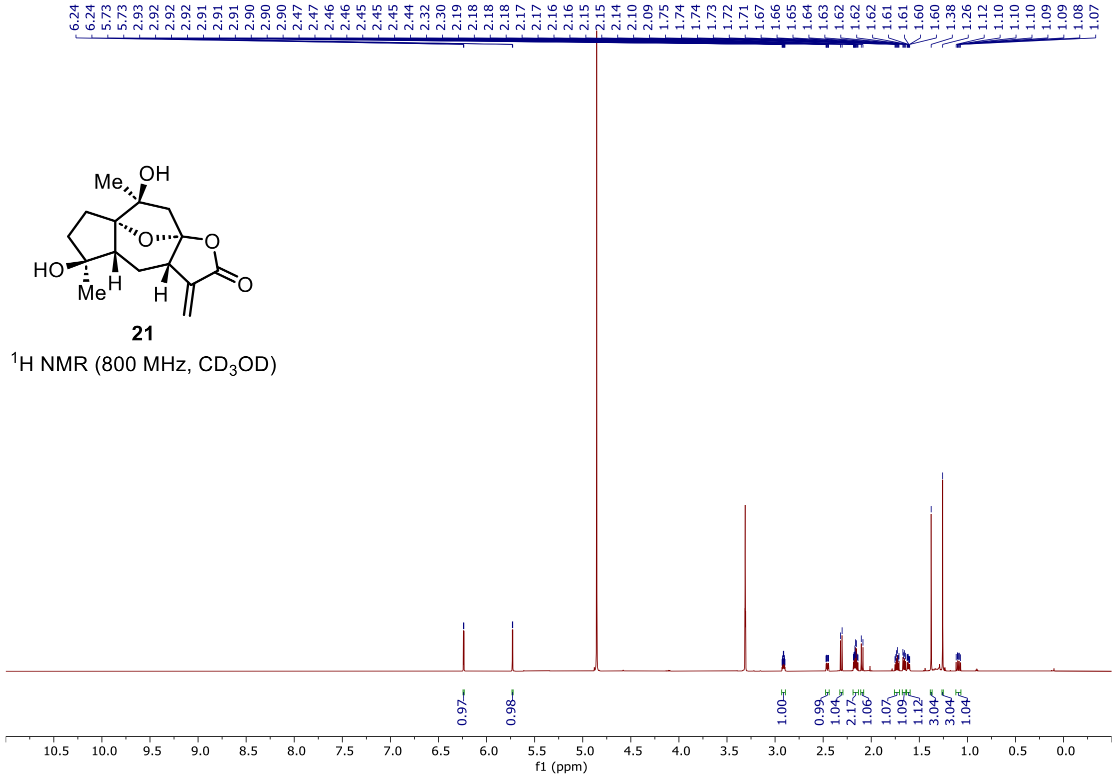
6.24 6.24 5.73 5.73 2.93 2.92 2.92 2.92 2.91 2.91 2.91 2.90 2.90 2.90 2.47 2.47 2.46 2.46 2.45 2.45 2.45 2.44 2.32 2.30 2.19 2.18 2.18 2.18 2.17 2.17 2.16 2.16 2.15 2.15 2.14 2.10 2.09 1.75 1.74 1.74 1.73 1.72 1.71 1.67 1.66 1.65 1.64 1.63 1.62 1.62 1.62 1.61 1.61 1.60 1.60 1.38 1.26 1.12 1.10 1.10 1.10 1.09 1.09 1.08 1.07
Me
OH
O
O
O
HO
Me
H
H
21
^{1}H NMR (800 MHz, CD_3OD)
0.97
0.98
1.00
0.99
1.04
2.17
1.06
1.07
1.09
1.12
3.04
3.04
1.04
10.5 10.0 9.5 9.0 8.5 8.0 7.5 7.0 6.5 6.0 5.5 5.0 4.5 4.0 3.5 3.0 2.5 2.0 1.5 1.0 0.5 0.0
f1 (ppm)

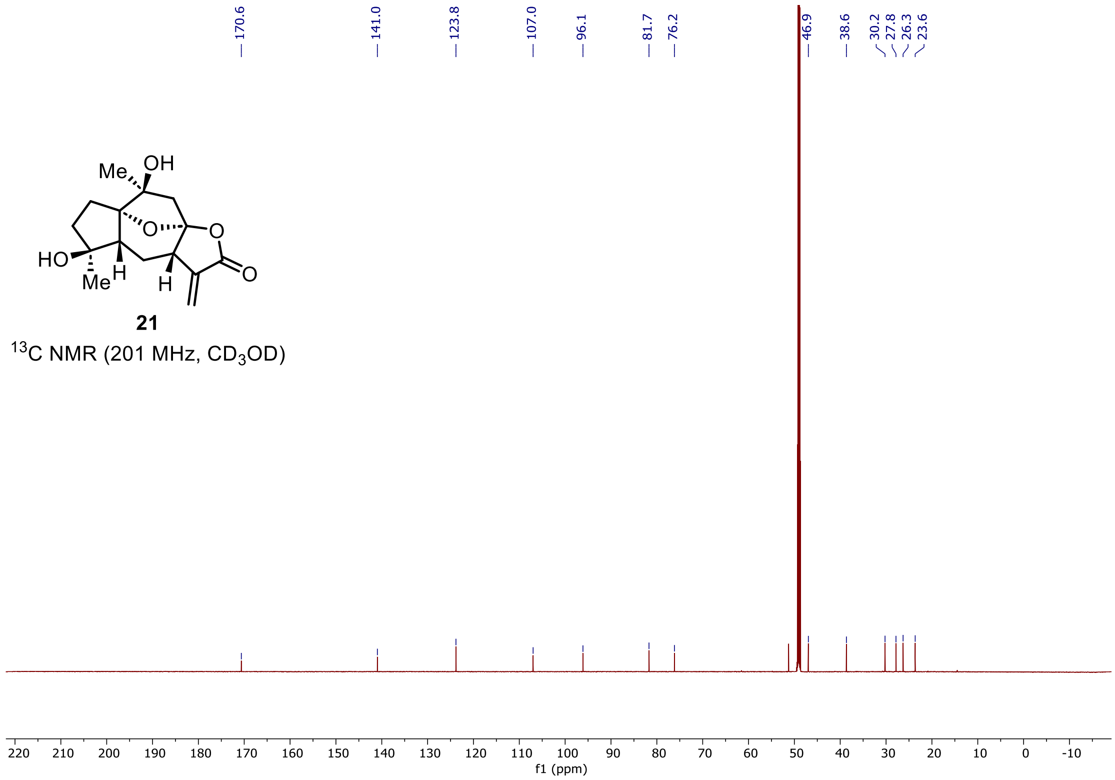
170.6
141.0
123.8
107.0
96.1
81.7
76.2
46.9
38.6
30.2
27.8
26.3
23.6
Me
OH
O
O
O
HO
Me
H
H
21
^{13}C NMR (201 MHz, CD_3OD)
220 210 200 190 180 170 160 150 140 130 120 110 100 90 80 70 60 50 40 30 20 10 0 -10
f1 (ppm)

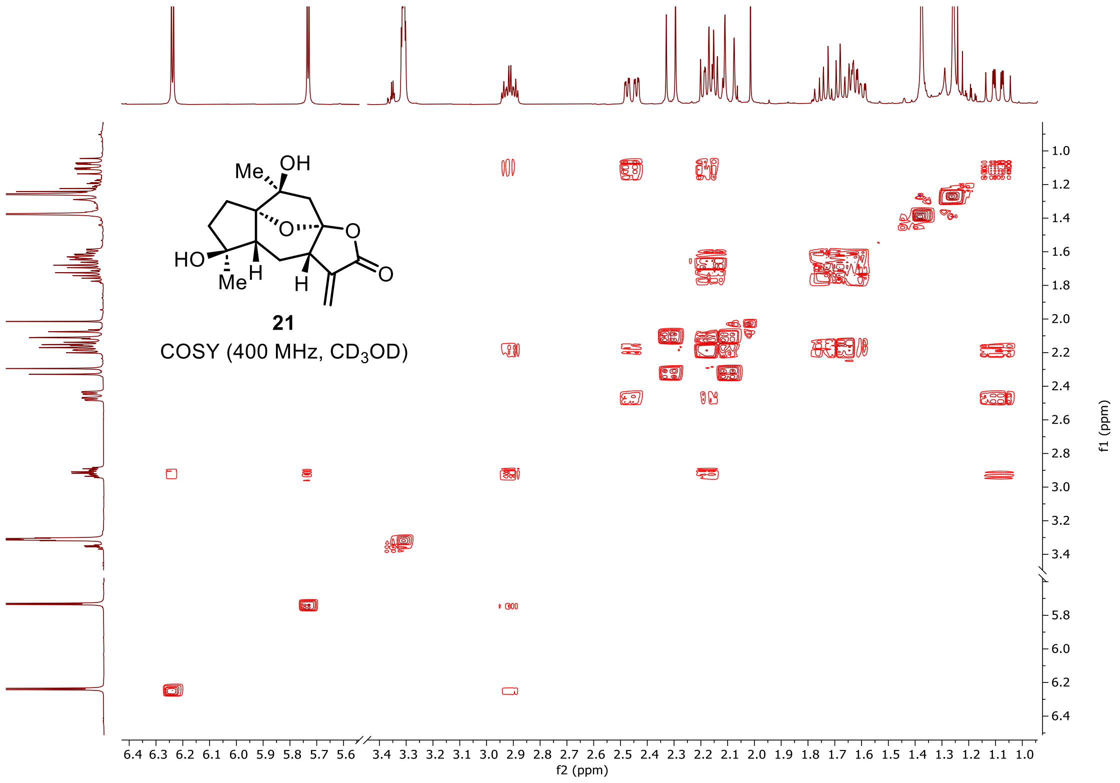
Me
OH
O
O
O
HO
Me
H
H
21
COSY (400 MHz, CD3OD)
f2 (ppm)
f1 (ppm)

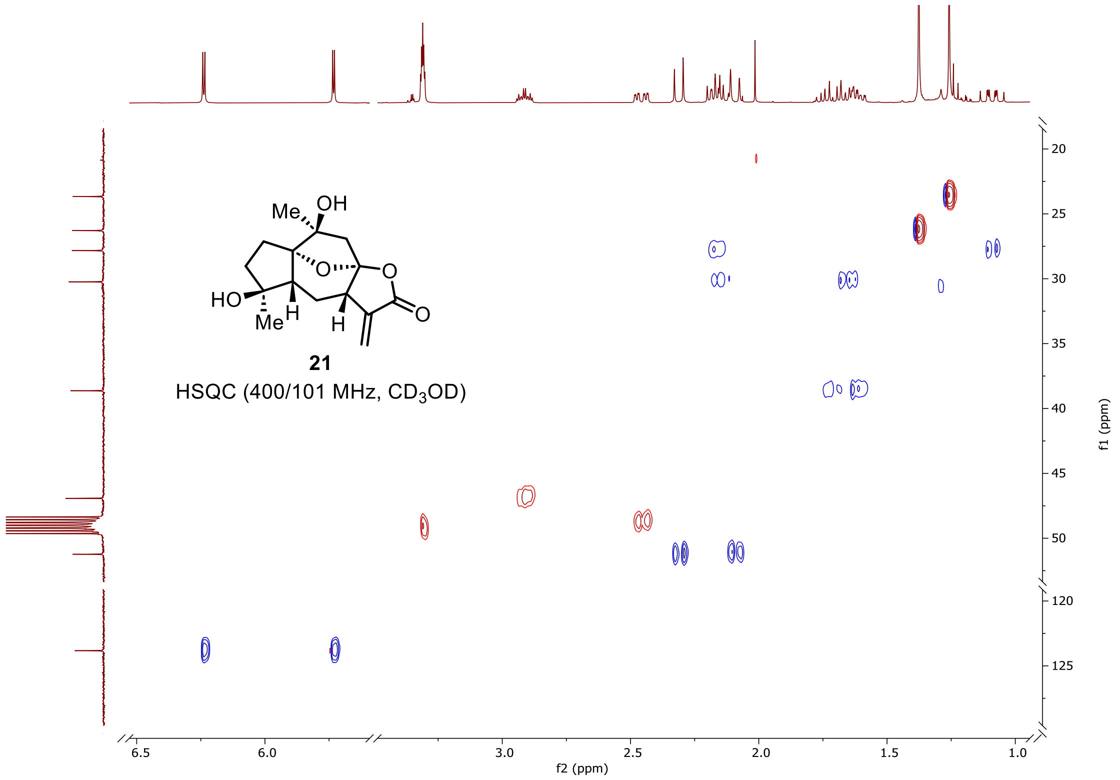
Me
OH
O
O
O
HO
Me
H
H
21
HSQC (400/101 MHz, CD3OD)
f2 (ppm)
f1 (ppm)

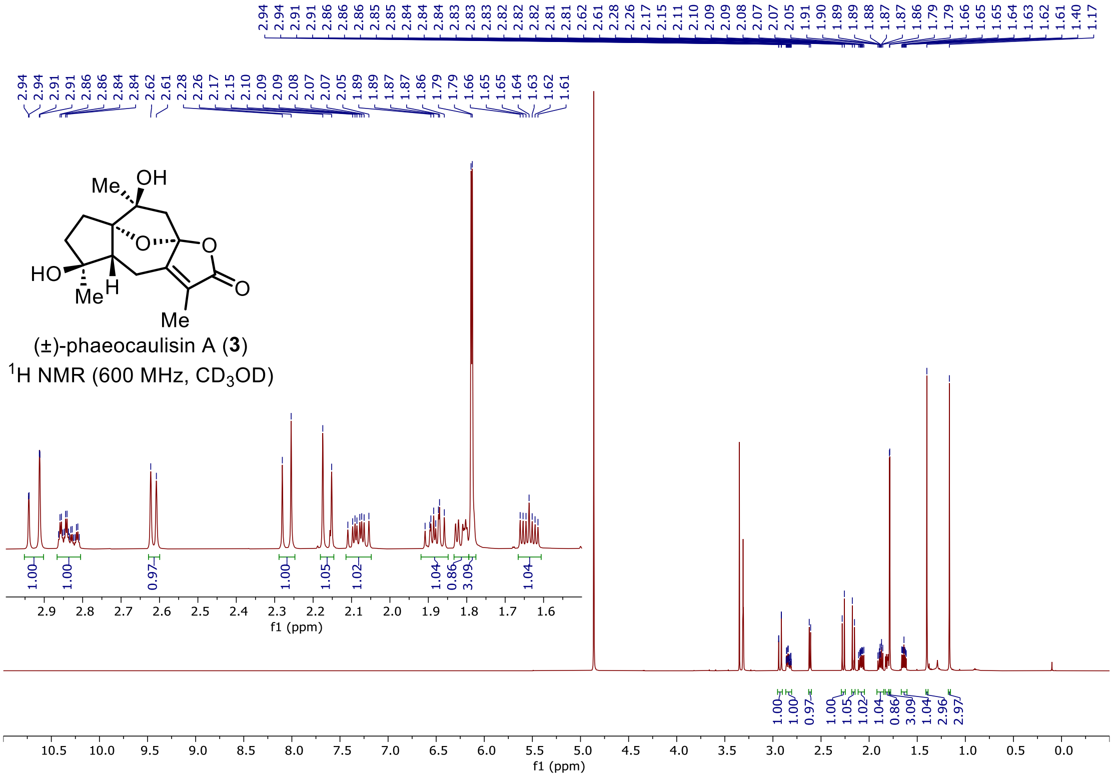
(±)-phaeocaulisin A (3)
^{1}H NMR (600 MHz, CD_3OD)
f1 (ppm)

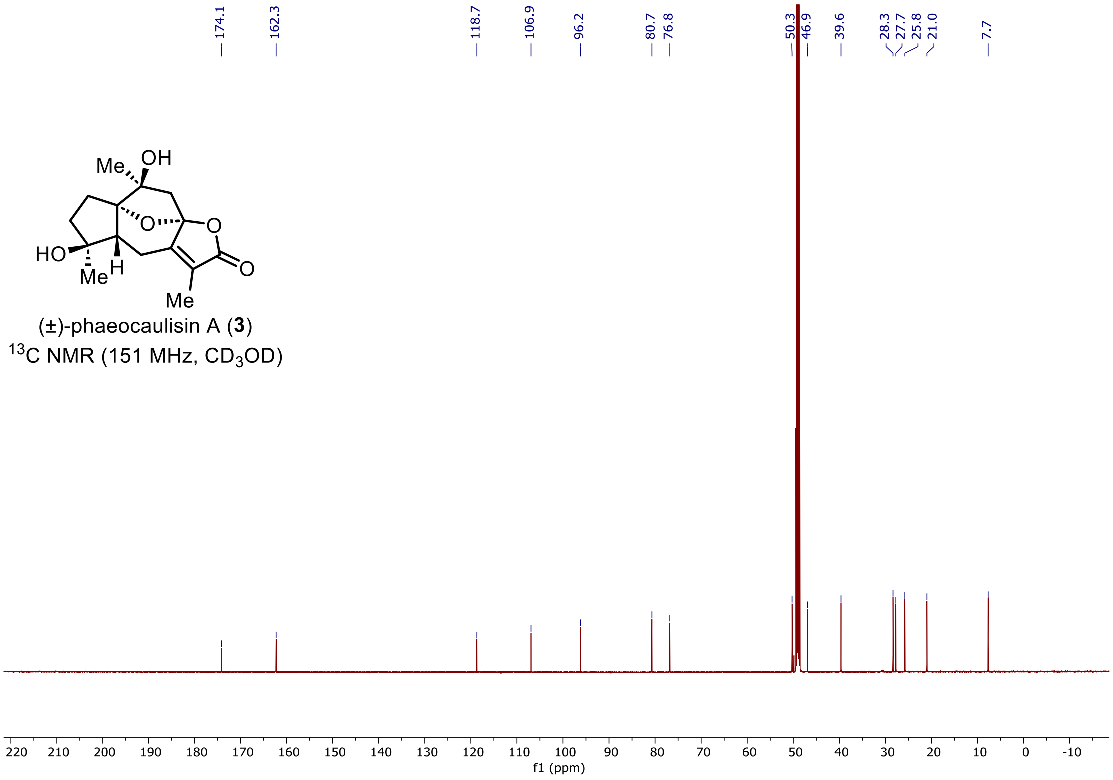
(±)-phaeocaulisin A (3)
^{13}C NMR (151 MHz, CD_3OD)
174.1
162.3
118.7
106.9
96.2
80.7
76.8
50.3
46.9
39.6
28.3
27.7
25.8
21.0
7.7
f1 (ppm)

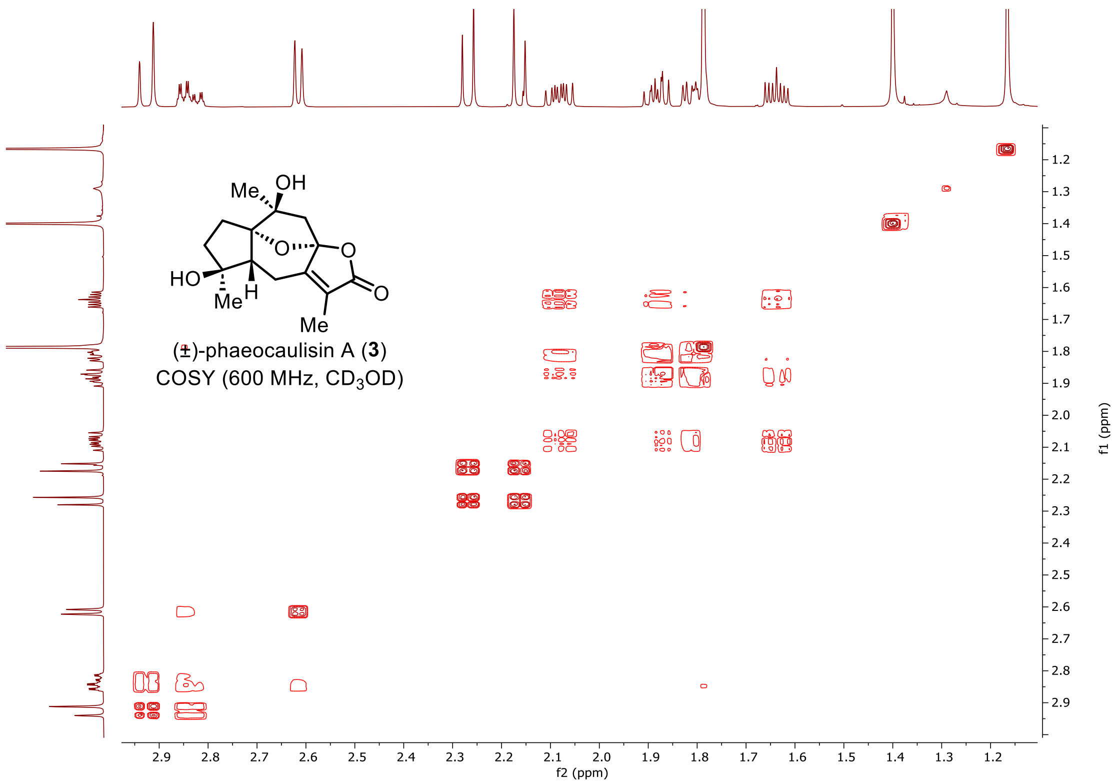
Me
OH
O
O
O
HO
Me
H
Me
(±)-phaeocaulisin A (3)
COSY (600 MHz, CD3OD)
f2 (ppm)
f1 (ppm)

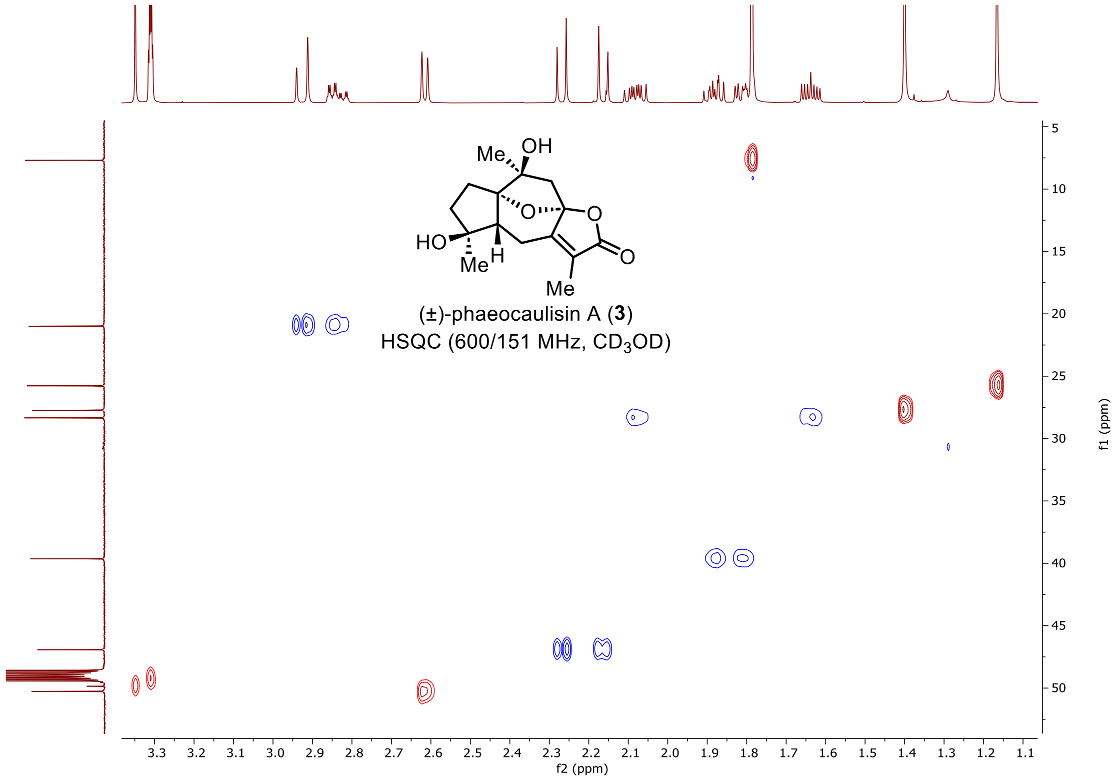
Me
OH
O
O
O
HO
Me
H
Me
(±)-phaeocaulisin A (3)
HSQC (600/151 MHz, CD3OD)
f2 (ppm)
f1 (ppm)

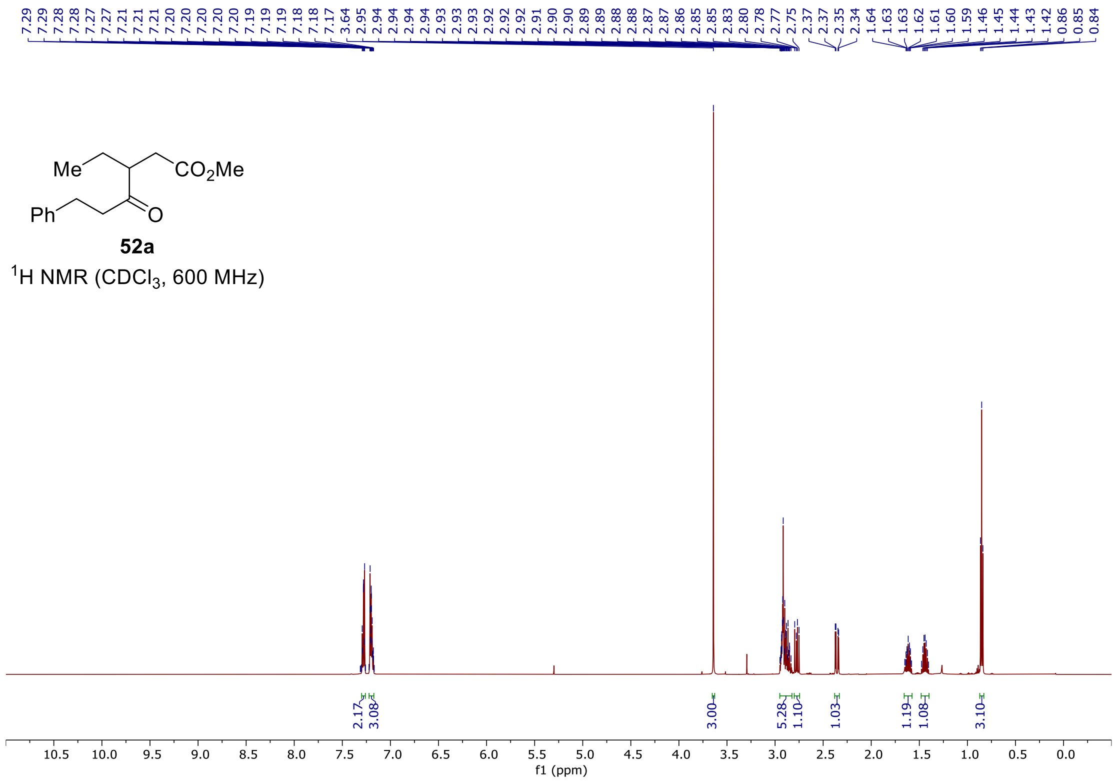
Me
CO2Me
Ph
O
52a
1H NMR (CDCl3, 600 MHz)
f1 (ppm)

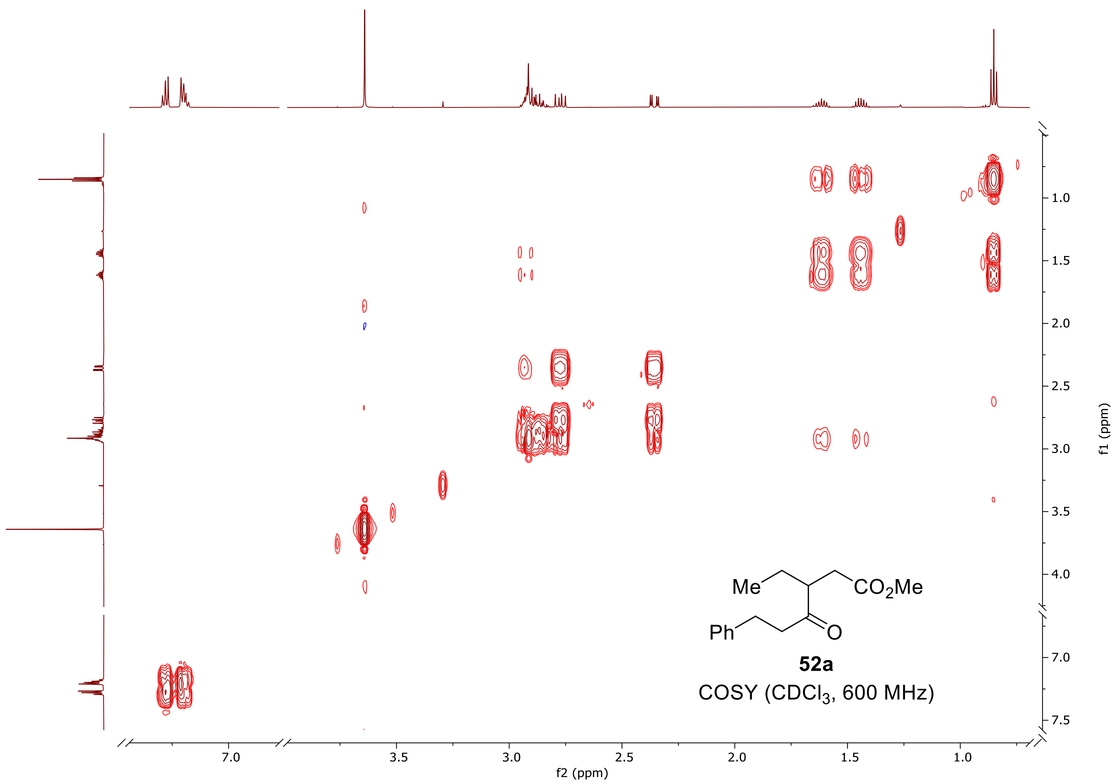
Me
CO2Me
Ph
O
52a
COSY (CDCl3, 600 MHz)
f2 (ppm)
f1 (ppm)

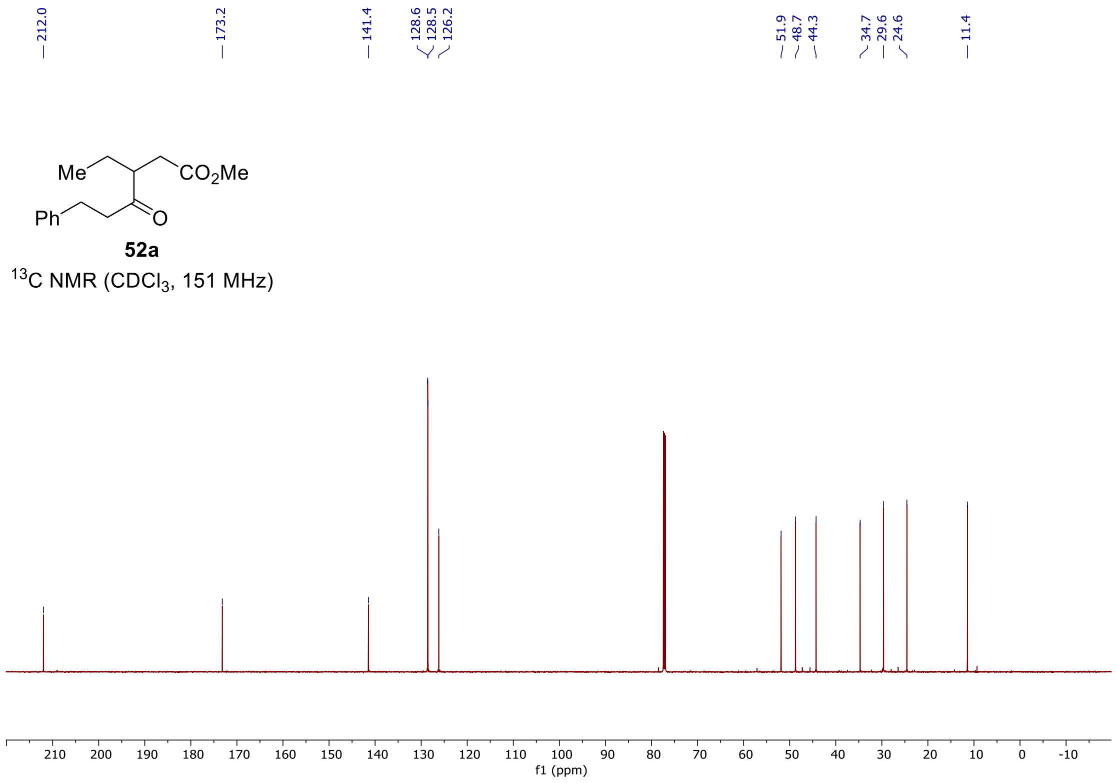
212.0
173.2
141.4
128.6
128.5
126.2
51.9
48.7
44.3
34.7
29.6
24.6
11.4
Me
CO2Me
Ph
O
52a
13C NMR (CDCl3, 151 MHz)
f1 (ppm)

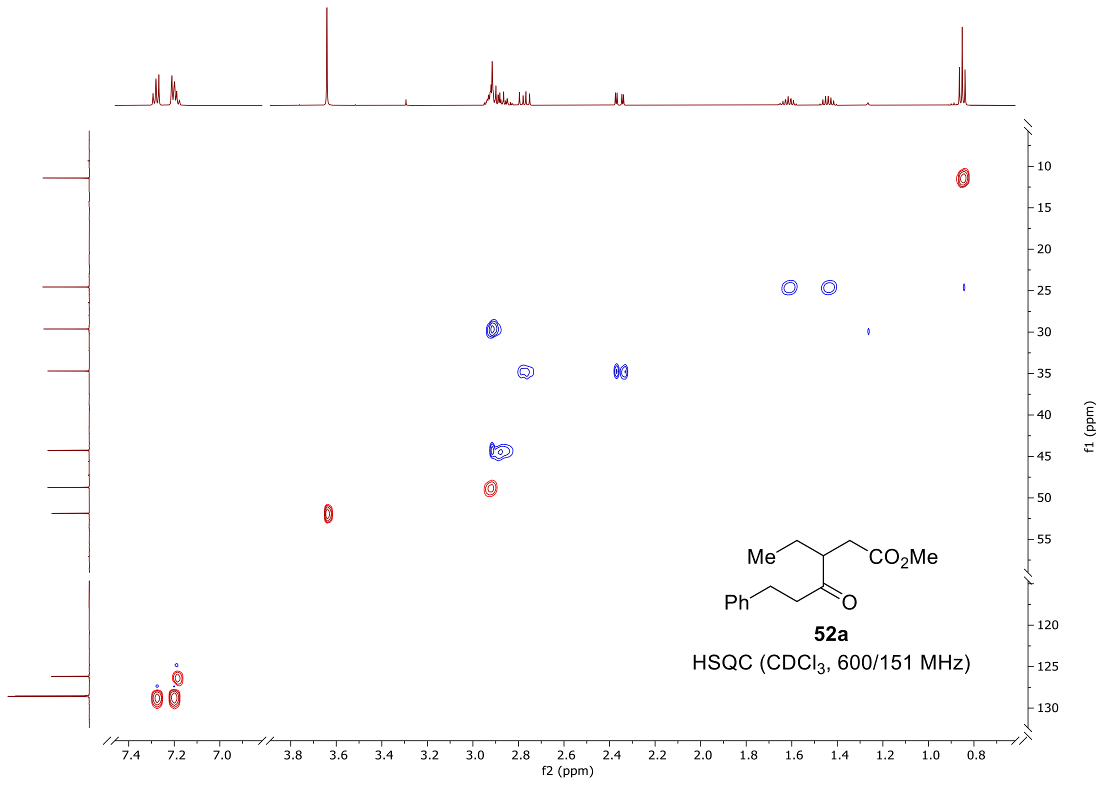
Me
CO2Me
Ph
O
52a
HSQC (CDCl3, 600/151 MHz)
f2 (ppm)
f1 (ppm)

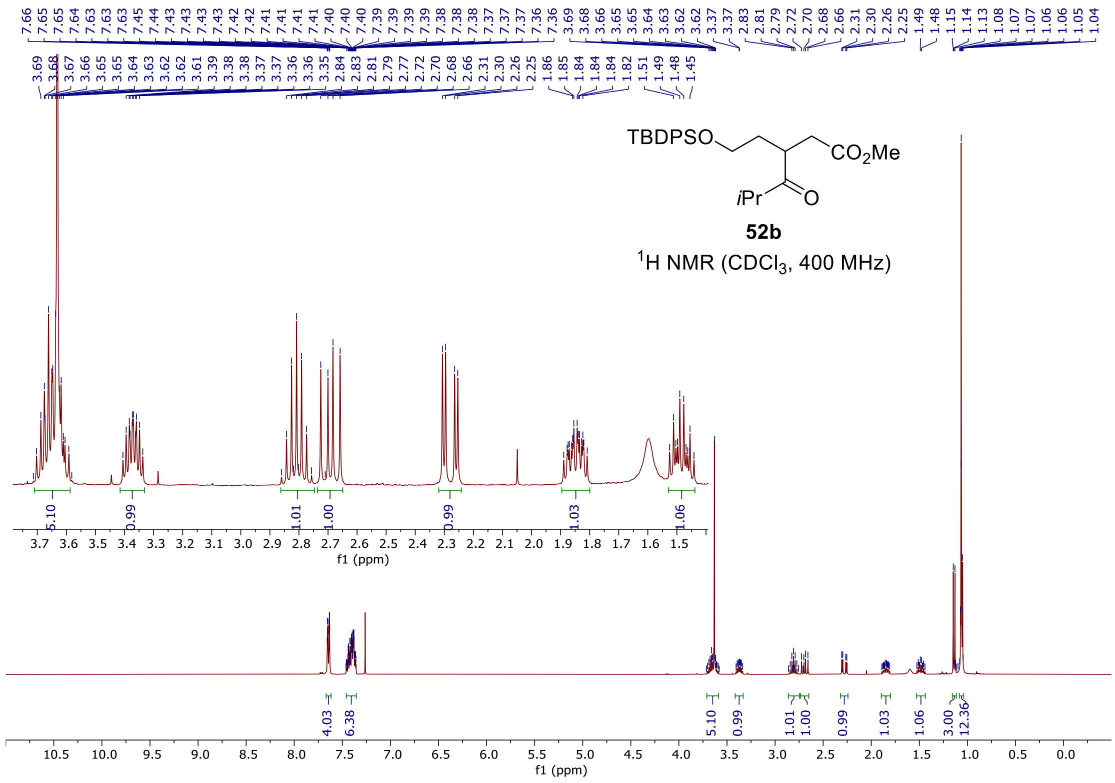
TBDPSO
CO2Me
iPr
O
52b
1H NMR (CDCl3, 400 MHz)
f1 (ppm)

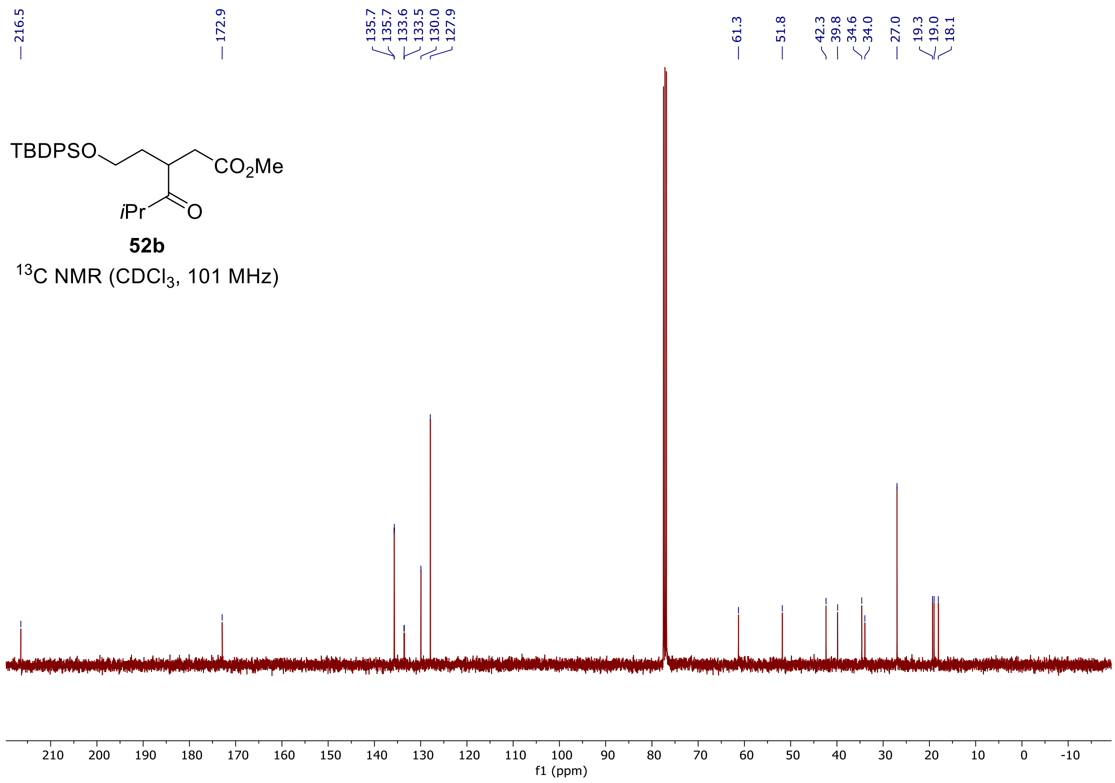
TBDPSO
CO2Me
iPr
O
52b
13C NMR (CDCl3, 101 MHz)
f1 (ppm)

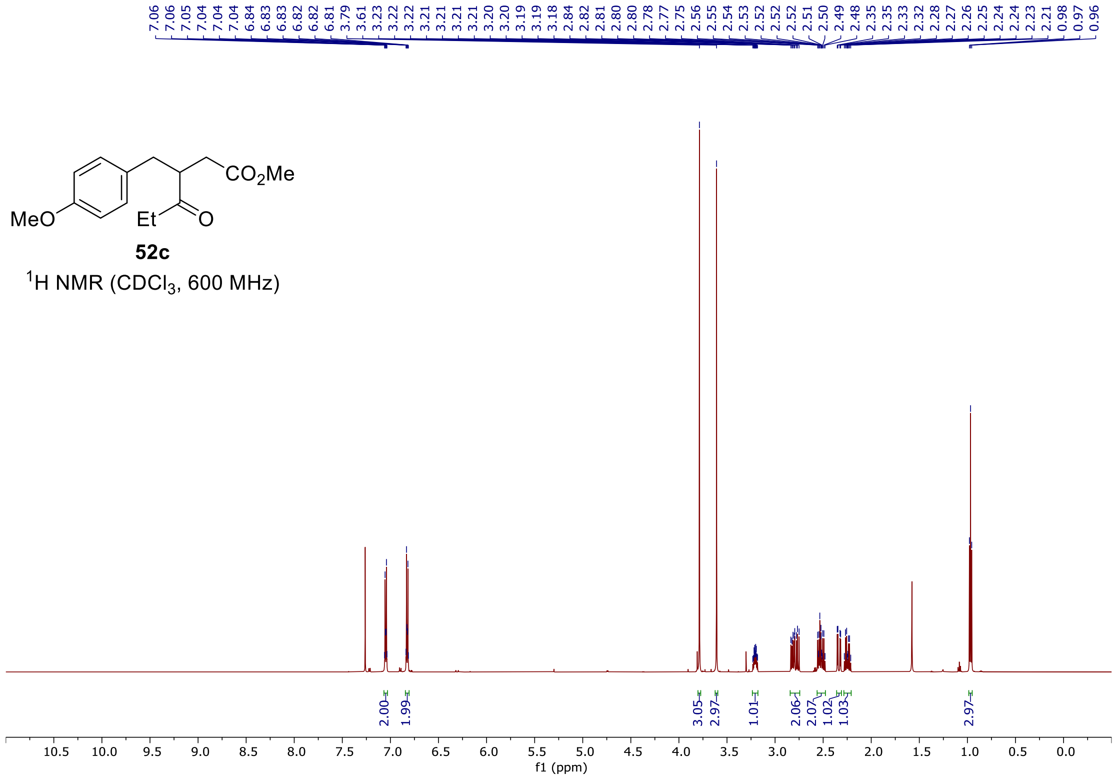
CO2Me
MeO
Et
O
52c
1H NMR (CDCl3, 600 MHz)
f1 (ppm)

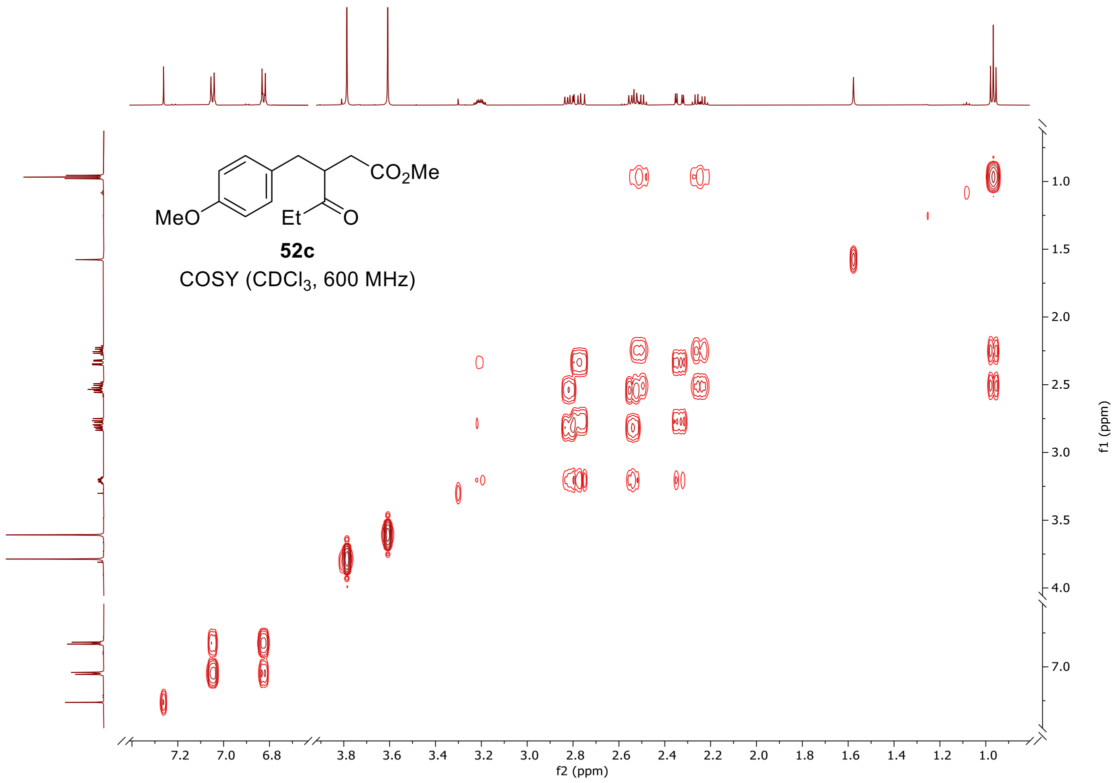
CO2Me
MeO
Et
O
52c
COSY (CDCl3, 600 MHz)
f2 (ppm)
f1 (ppm)

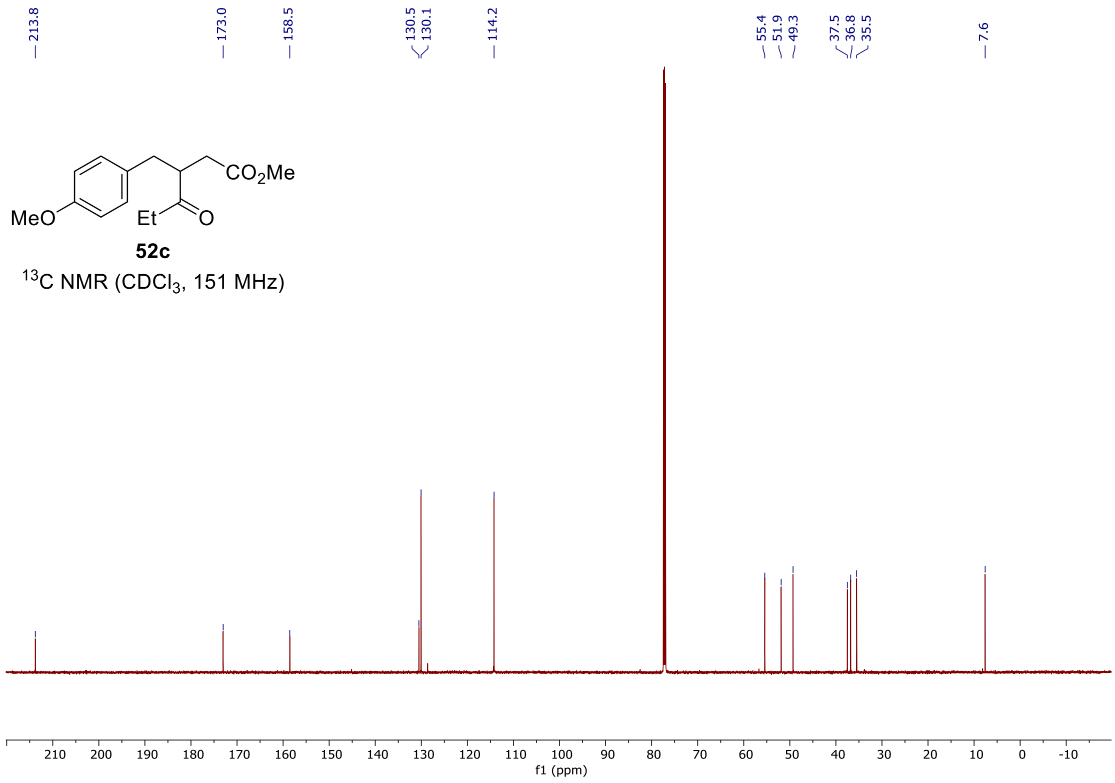
213.8
173.0
158.5
130.5
130.1
114.2
55.4
51.9
49.3
37.5
36.8
35.5
7.6
CO2Me
MeO
Et
O
52c
13C NMR (CDCl3, 151 MHz)
f1 (ppm)

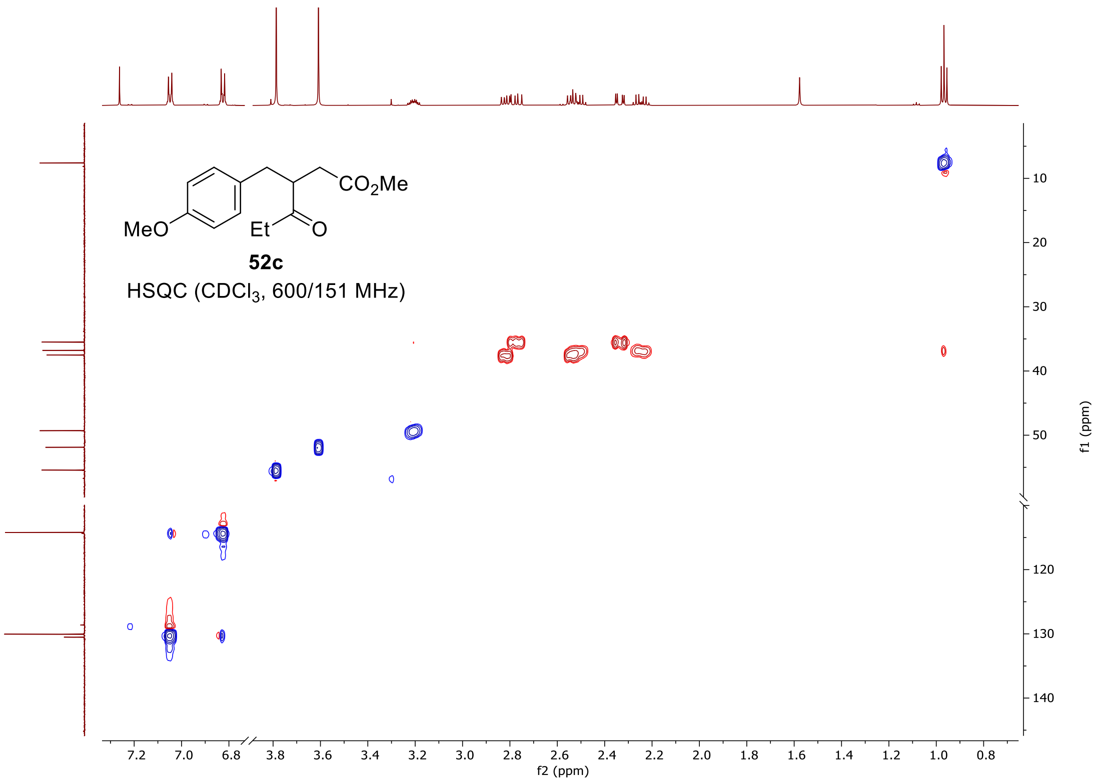
CO2Me
MeO
Et
O
52c
HSQC (CDCl3, 600/151 MHz)
f2 (ppm)
f1 (ppm)

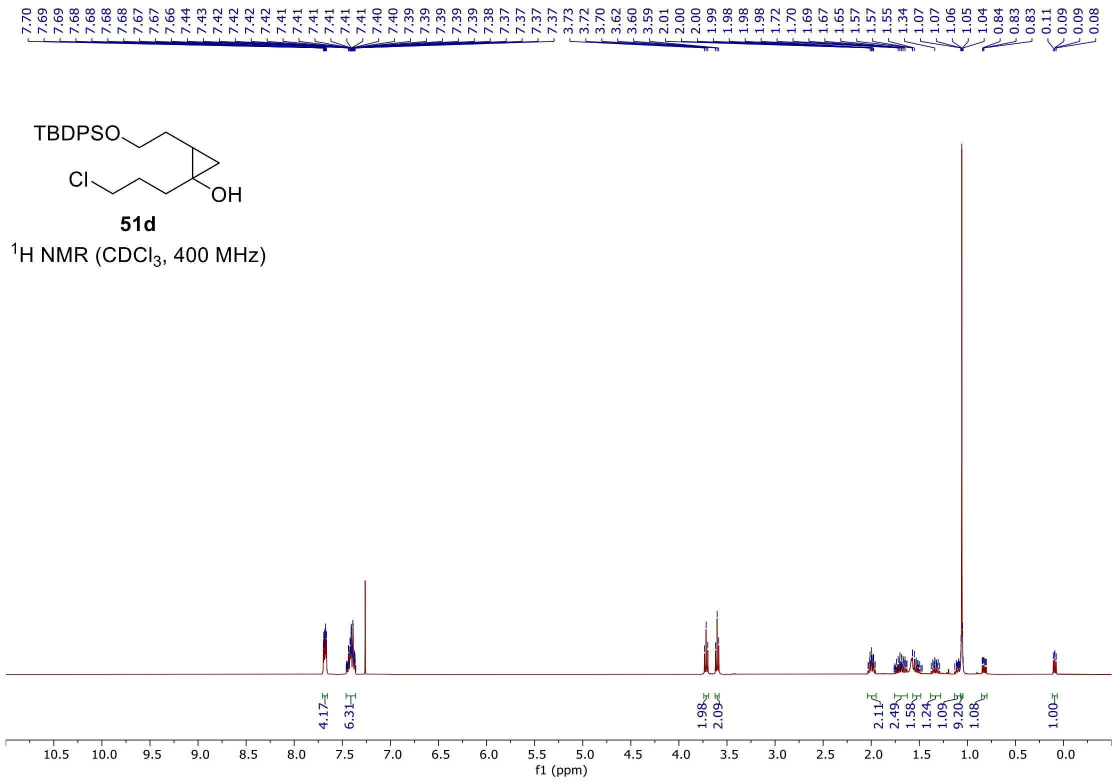
7.70 7.69 7.68 7.68 7.68 7.67 7.67 7.66 7.44 7.43 7.42 7.42 7.42 7.42 7.41 7.41 7.41 7.41 7.41 7.40 7.39 7.39 7.39 7.39 7.38 7.37 7.37 7.37 3.73 3.72 3.70 3.62 3.60 3.59 2.01 2.00 1.99 1.98 1.98 1.98 1.72 1.70 1.69 1.67 1.65 1.57 1.55 1.34 1.07 1.07 1.06 1.05 1.04 0.84 0.83 0.83 0.11 0.09 0.08
TBDPSO
Cl
OH
51d
¹H NMR (CDCl3, 400 MHz)
4.17 6.31 1.98 2.09 2.11 2.49 1.58 1.24 1.09 9.20 1.08 1.00
f1 (ppm)

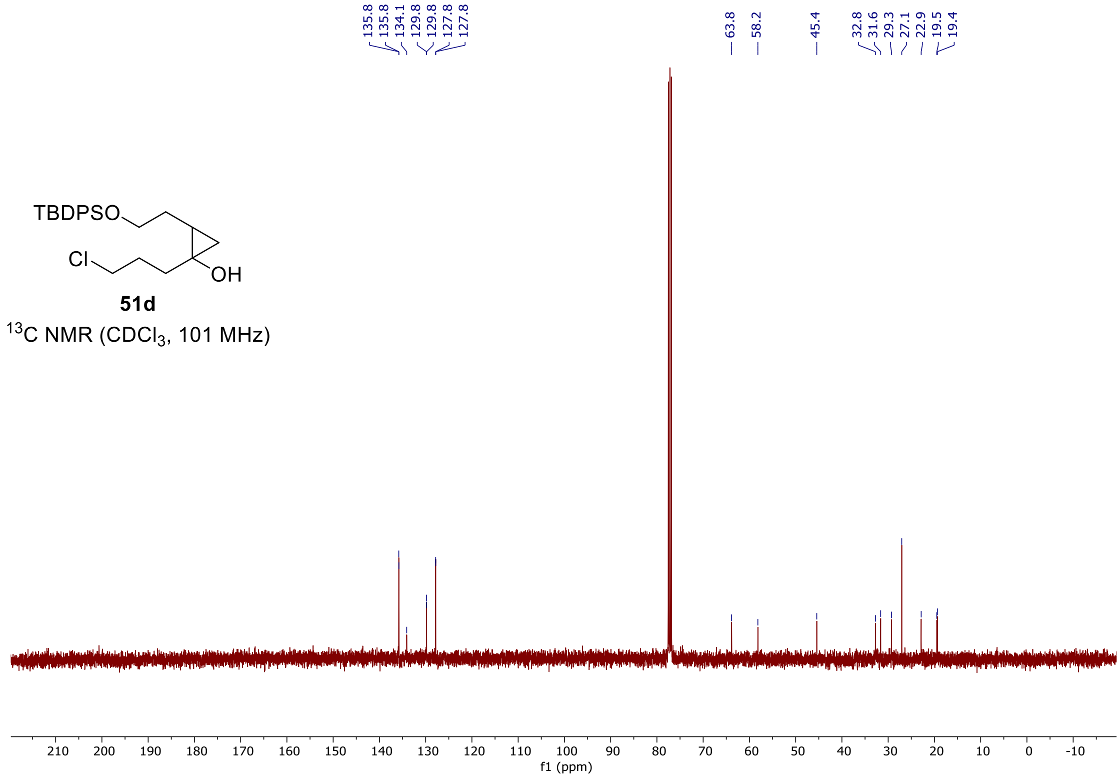
135.8 135.8 134.1 129.8 129.8 127.8 127.8
63.8 58.2 45.4 32.8 31.6 29.3 27.1 22.9 19.5 19.4
TBDPSO
Cl
OH
51d
¹³C NMR (CDCl3, 101 MHz)
f1 (ppm)

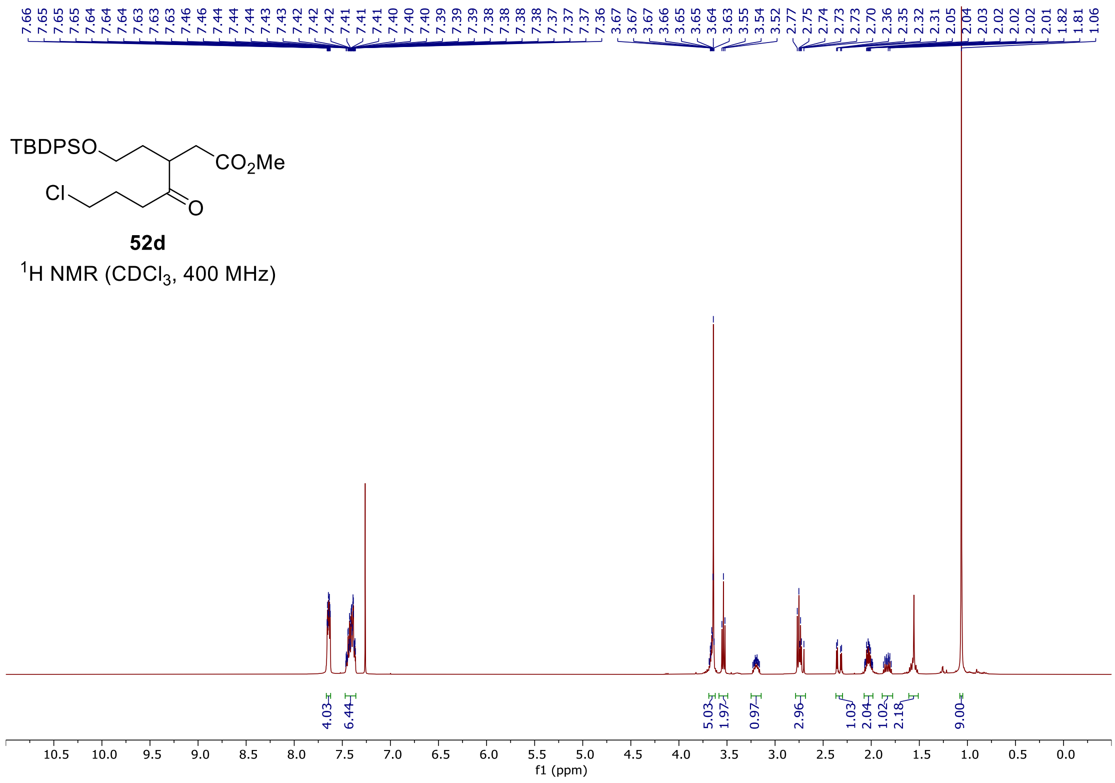
TBDPSO
CO2Me
Cl
O
52d
1H NMR (CDCl3, 400 MHz)
f1 (ppm)

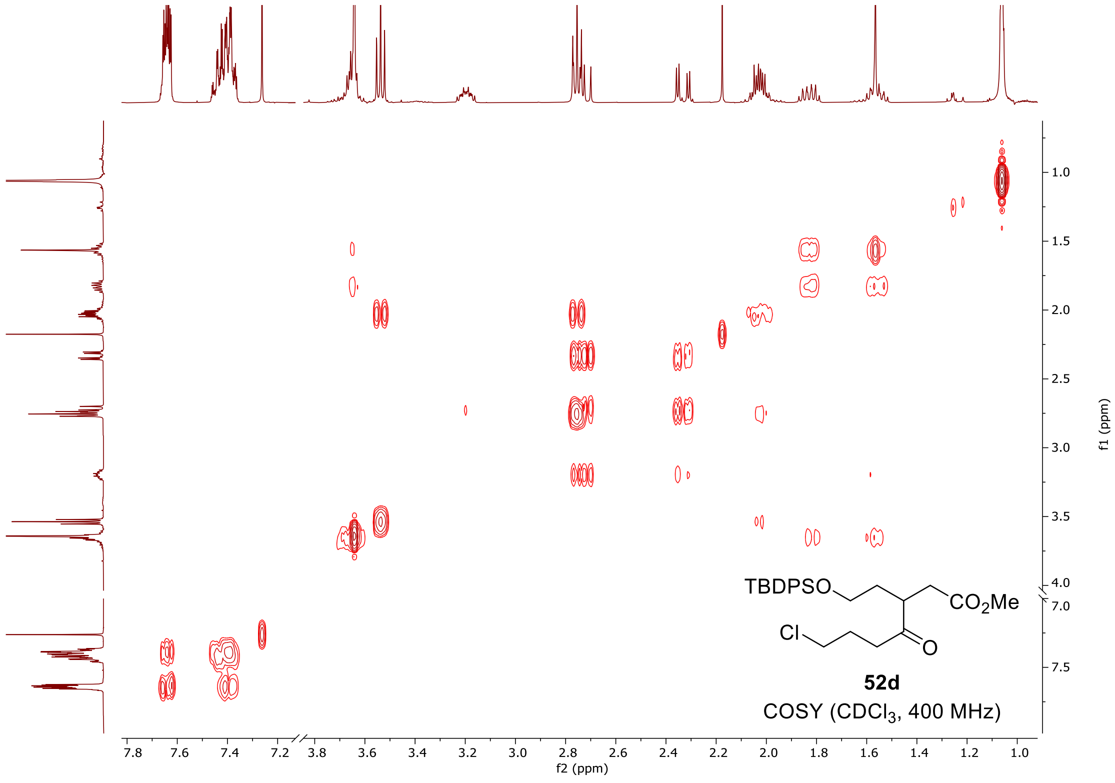
TBDPSO
CO2Me
Cl
O
52d
COSY (CDCl3, 400 MHz)
f2 (ppm)
f1 (ppm)

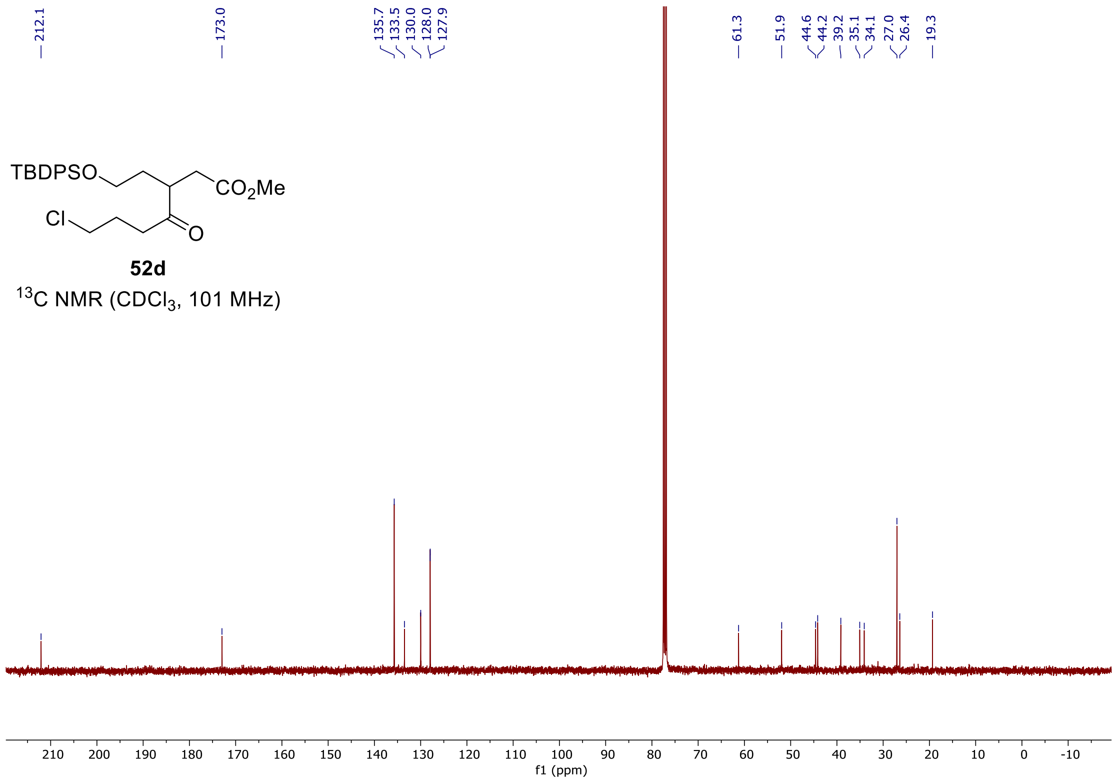
212.1
173.0
135.7
133.5
130.0
128.0
127.9
61.3
51.9
44.6
44.2
39.2
35.1
34.1
27.0
26.4
19.3
TBDPSO
CO2Me
Cl
O
52d
13C NMR (CDCl3, 101 MHz)
f1 (ppm)

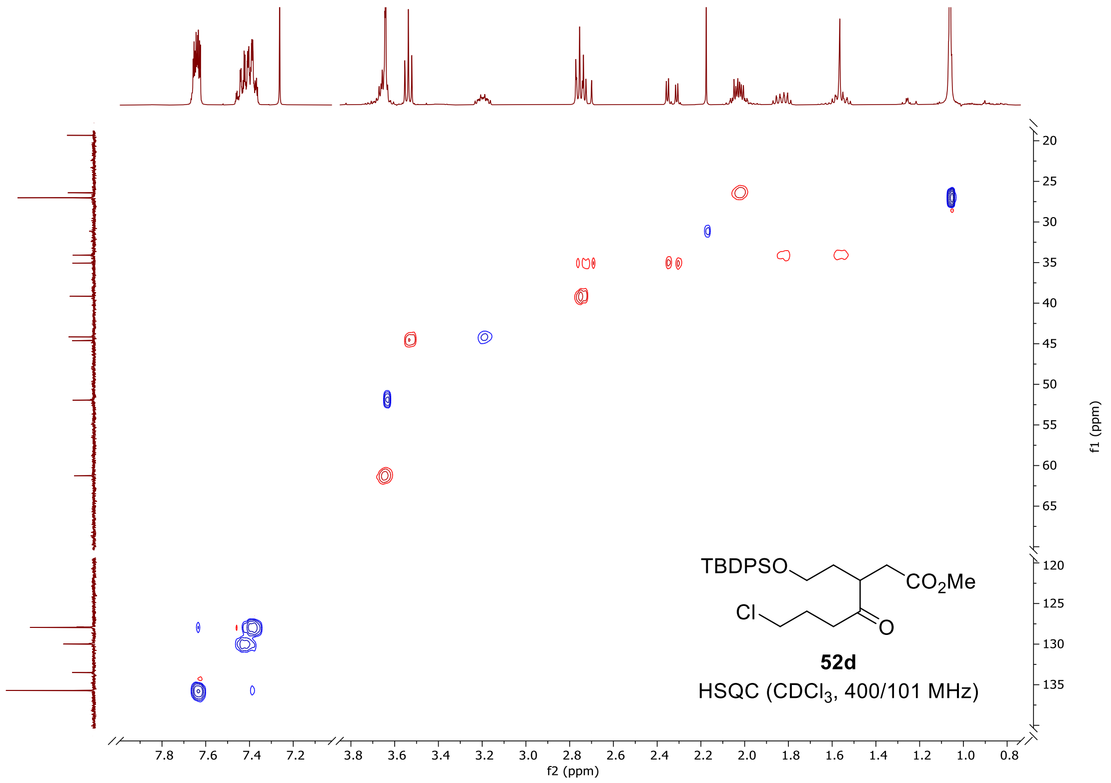
TBDPSO
CO2Me
Cl
O
52d
HSQC (CDCl3, 400/101 MHz)
f2 (ppm)
f1 (ppm)

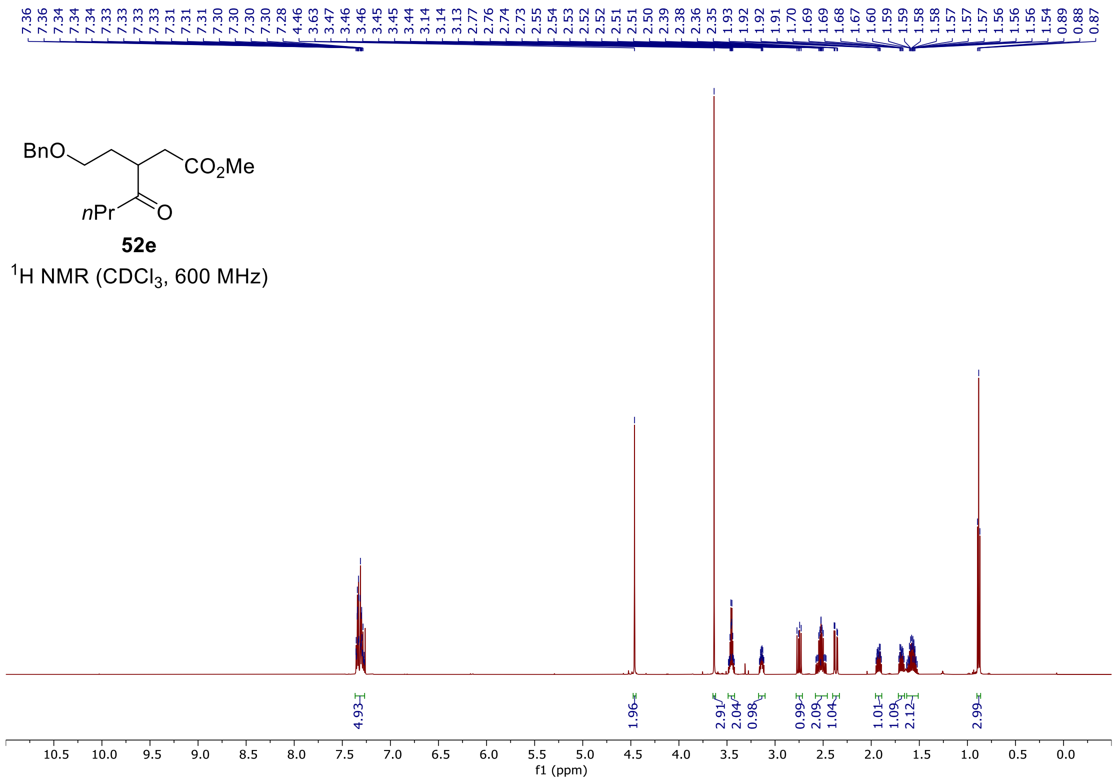
BnO
CO2Me
nPr
O
52e
1H NMR (CDCl3, 600 MHz)
f1 (ppm)

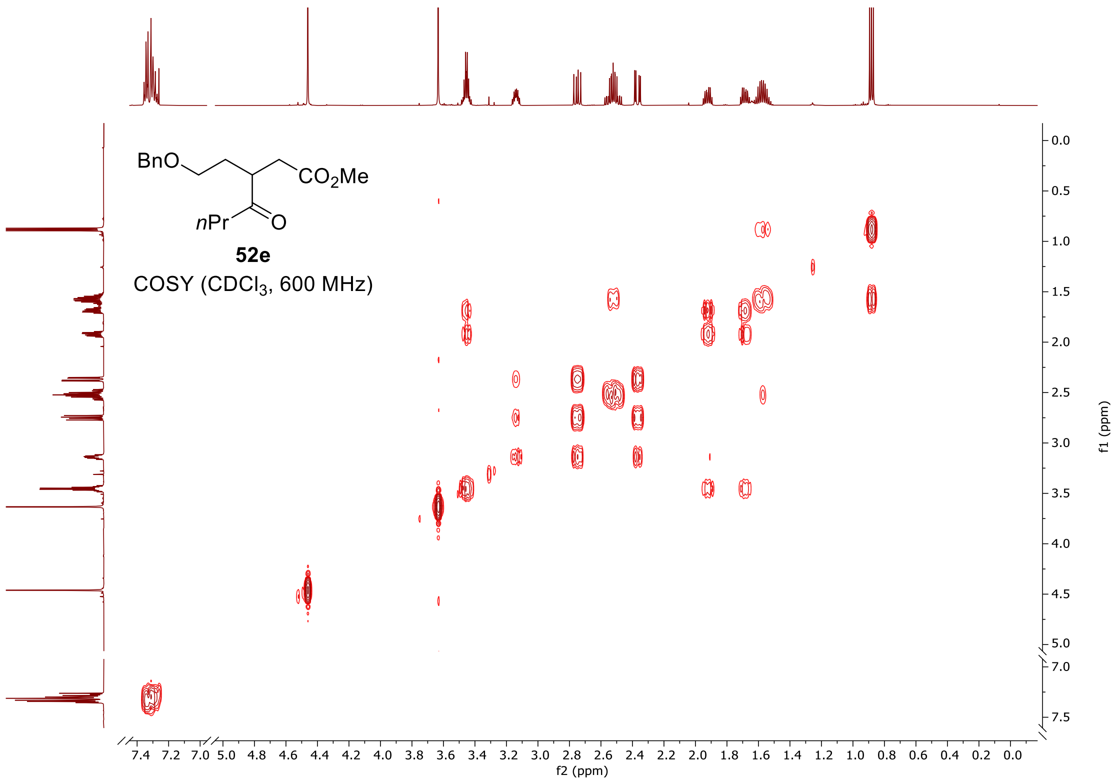
BnO
CO2Me
nPr
O
52e
COSY (CDCl3, 600 MHz)
f2 (ppm)
f1 (ppm)

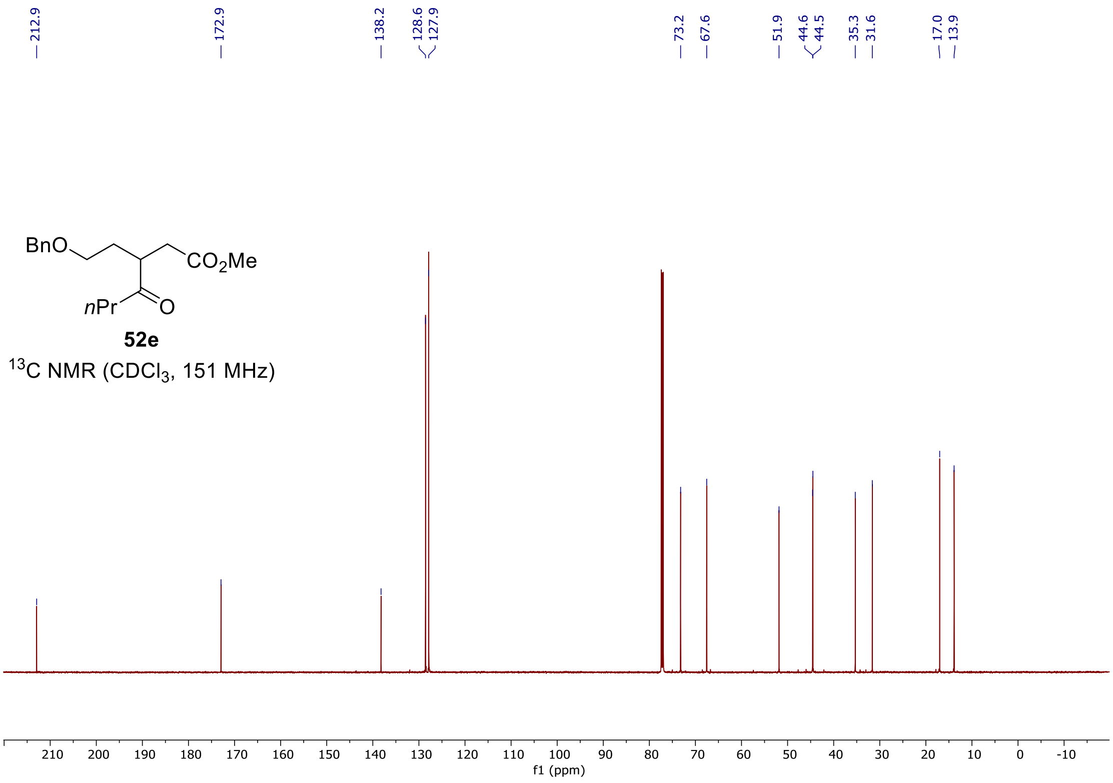
212.9
172.9
138.2
128.6
127.9
73.2
67.6
51.9
44.6
44.5
35.3
31.6
17.0
13.9
BnO
CO2Me
nPr
O
52e
13C NMR (CDCl3, 151 MHz)
f1 (ppm)

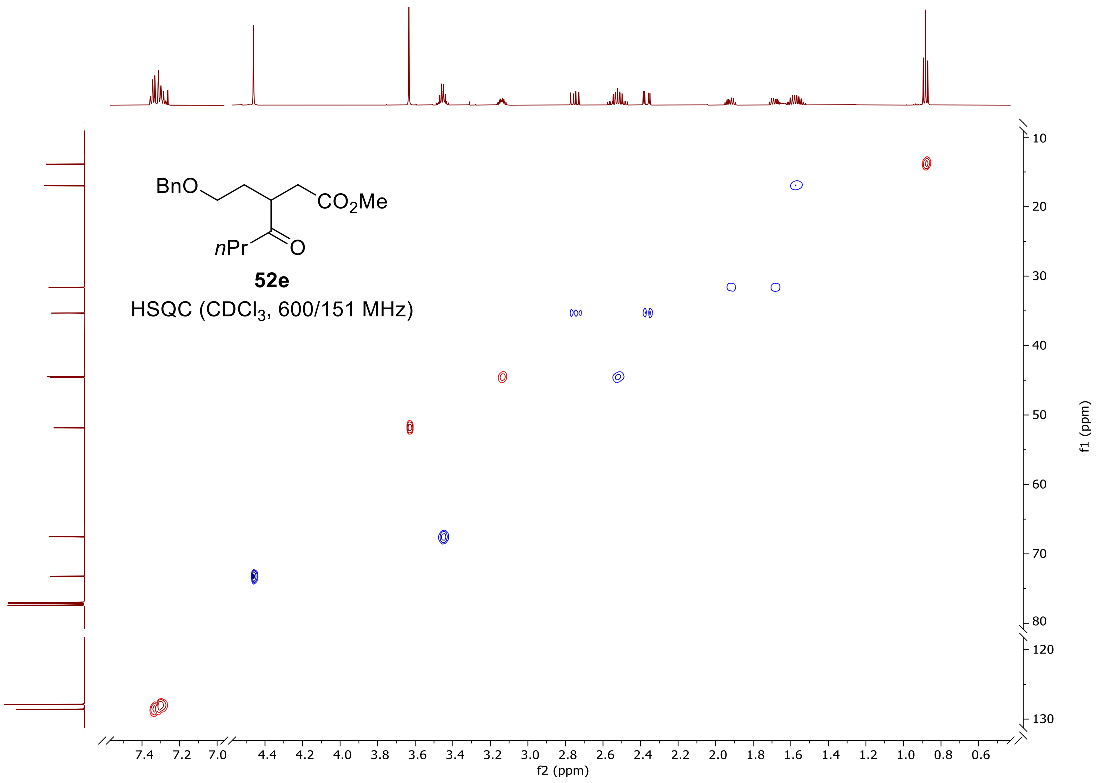
BnO
CO2Me
nPr
O
52e
HSQC (CDCl3, 600/151 MHz)
f2 (ppm)
f1 (ppm)

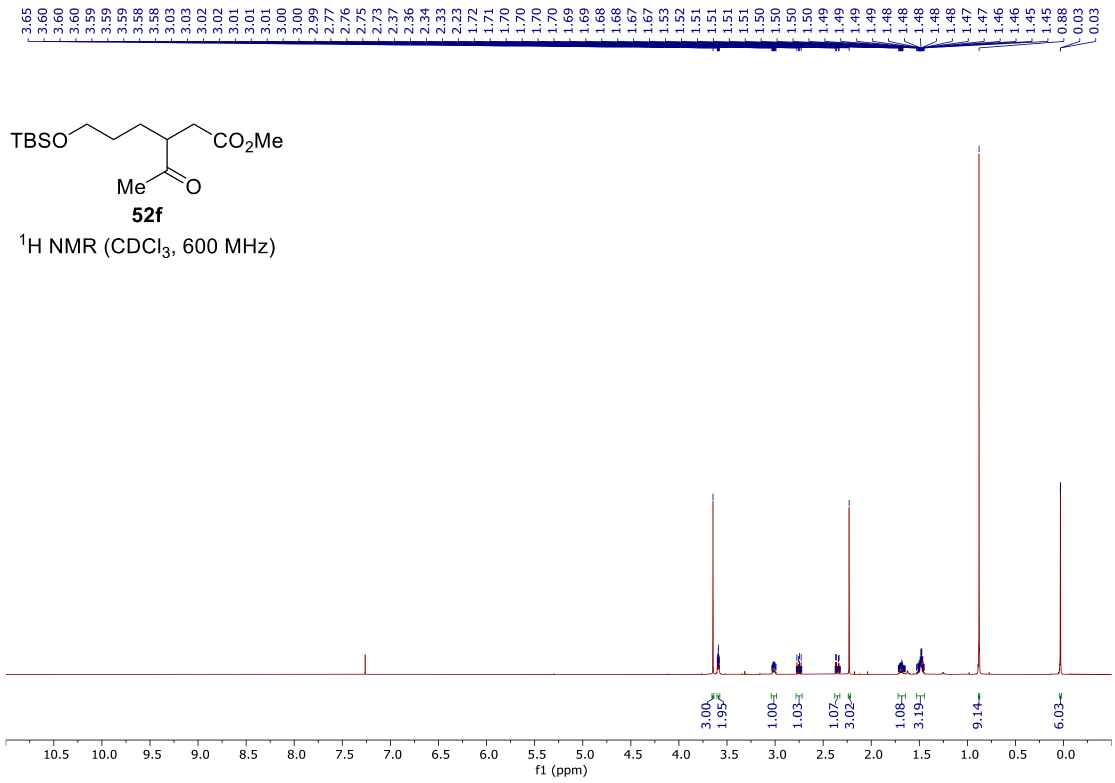
TBSO
CO_2Me
Me
O
52f
^1H NMR ($CDCl_3$, 600 MHz)
f1 (ppm)

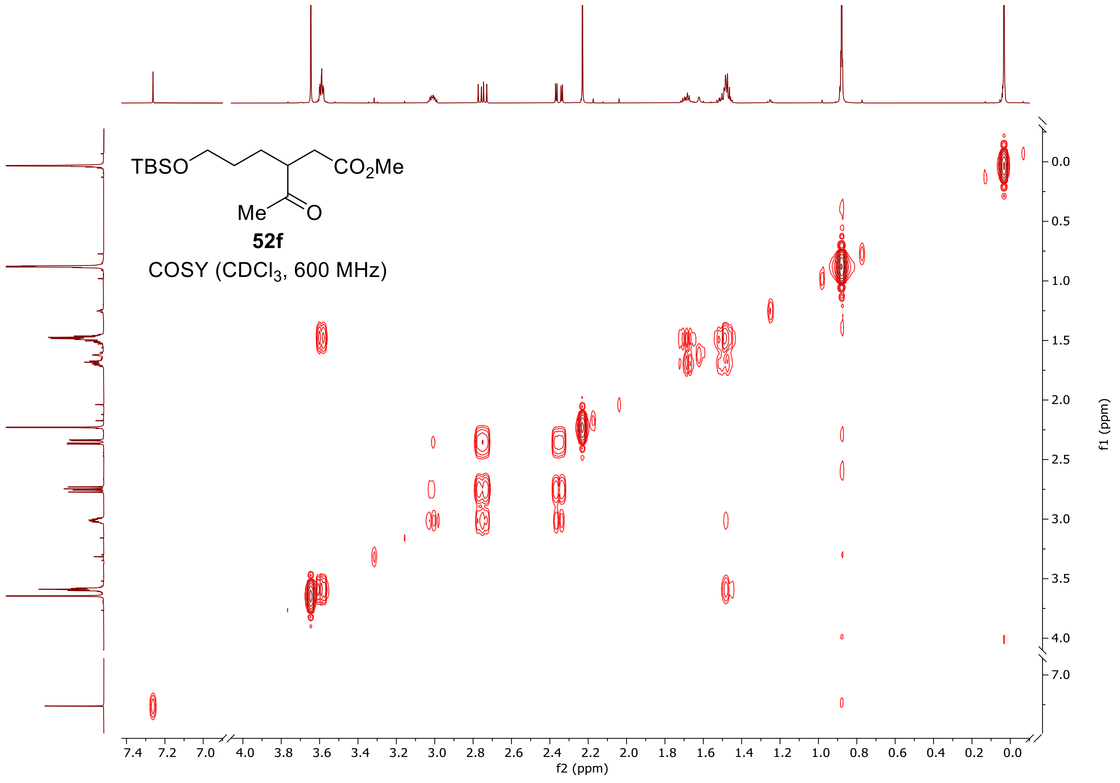
TBSO
CO_2Me
Me
O
52f
COSY ($CDCl_3$, 600 MHz)
f2 (ppm)
f1 (ppm)

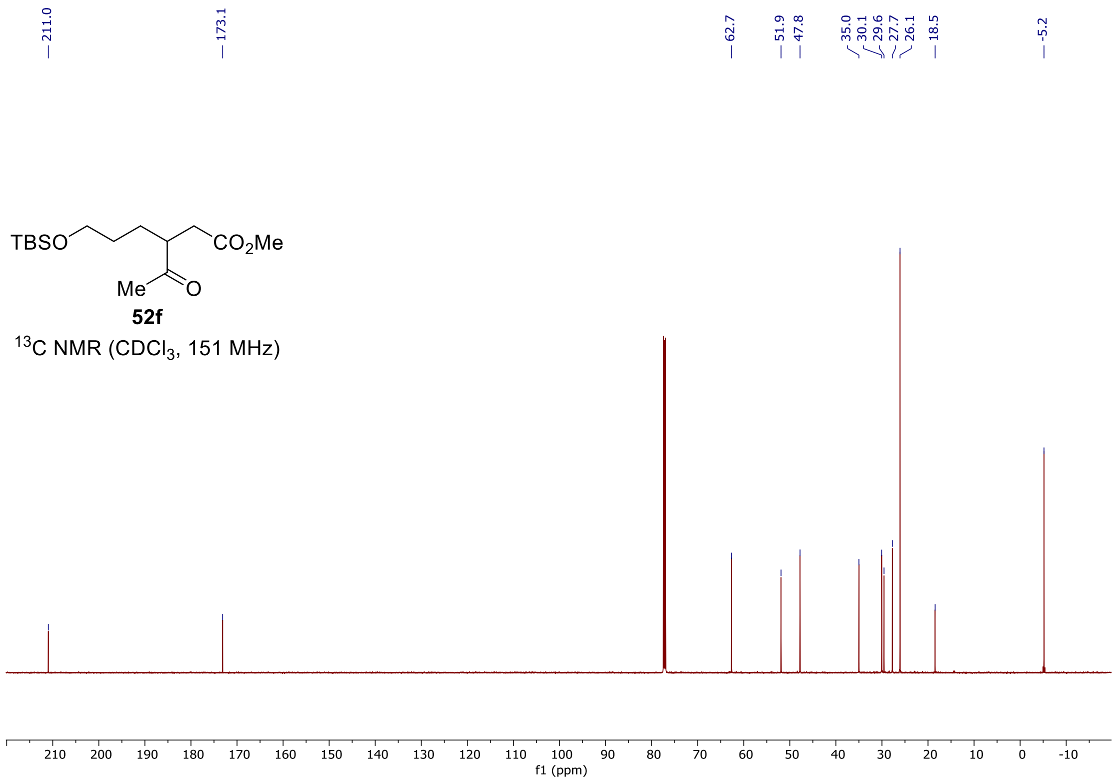

211.0
173.1
62.7
51.9
47.8
35.0
30.1
29.6
27.7
26.1
18.5
-5.2
TBSO
CO2Me
Me
O
52f
13C NMR (CDCl3, 151 MHz)
210 200 190 180 170 160 150 140 130 120 110 100 90 80 70 60 50 40 30 20 10 0 -10
f1 (ppm)

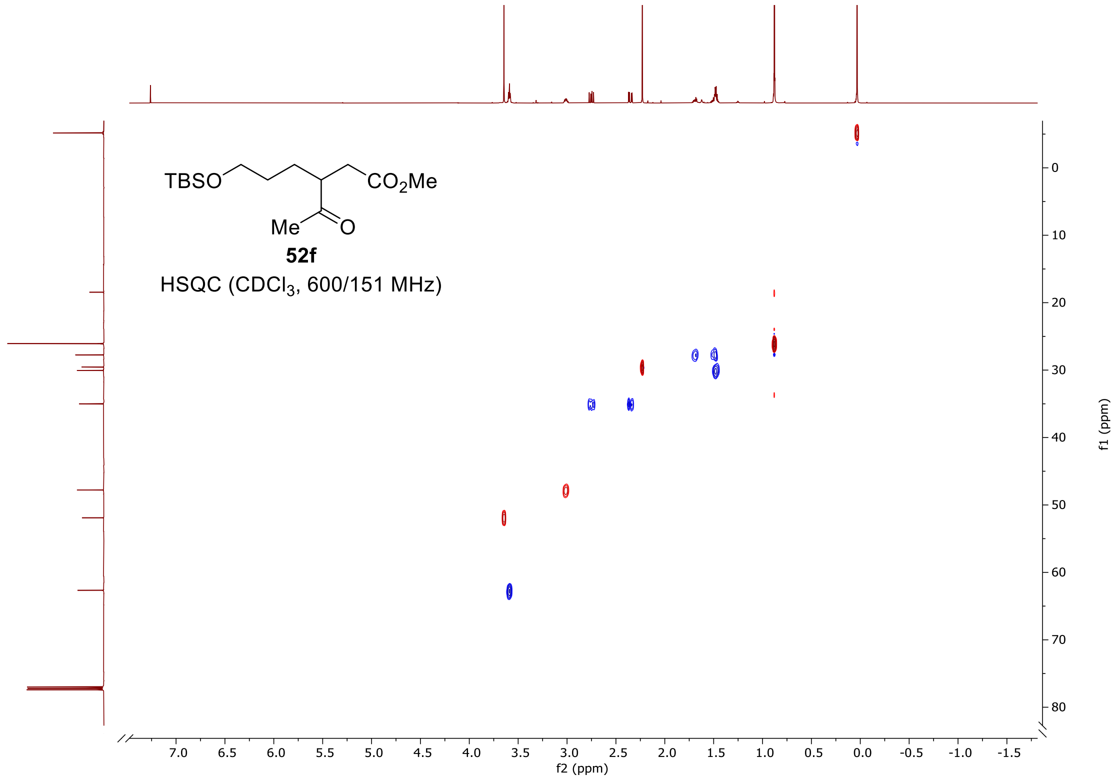

TBSO
CO2Me
Me
O
52f
HSQC (CDCl3, 600/151 MHz)
7.0 6.5 6.0 5.5 5.0 4.5 4.0 3.5 3.0 2.5 2.0 1.5 1.0 0.5 0.0 -0.5 -1.0 -1.5
f2 (ppm)
0 10 20 30 40 50 60 70 80
f1 (ppm)

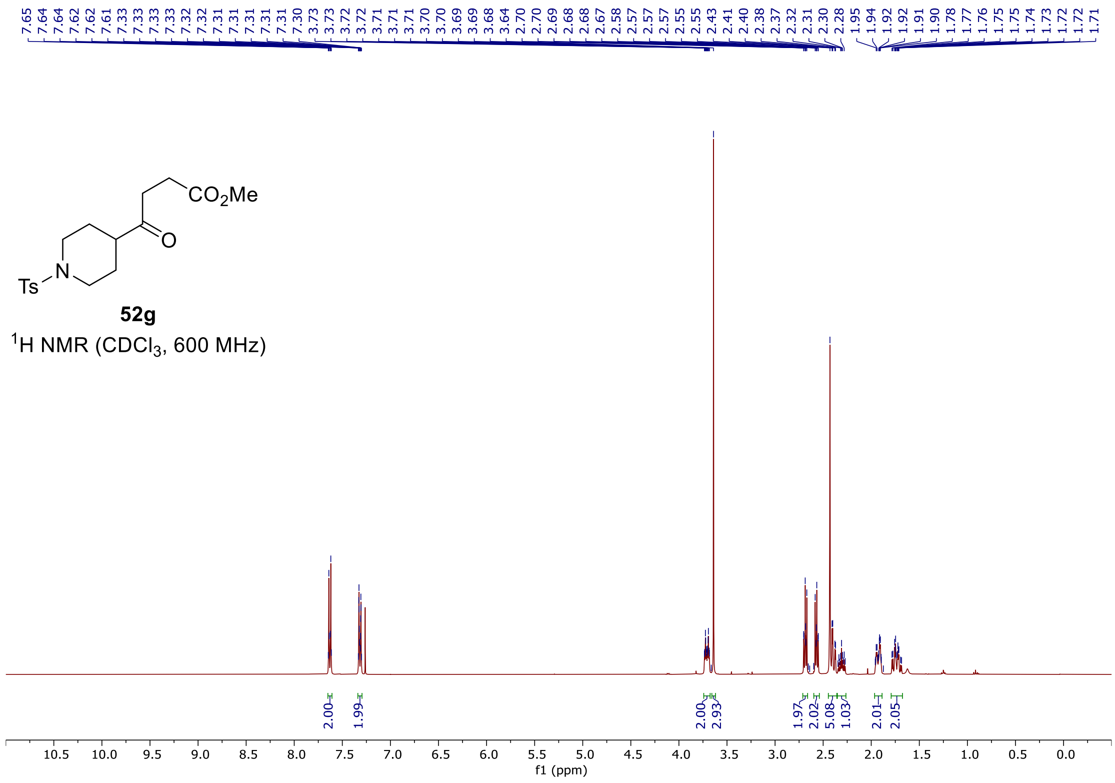

52g
^{1}H NMR ($CDCl_3$, 600 MHz)
f1 (ppm)

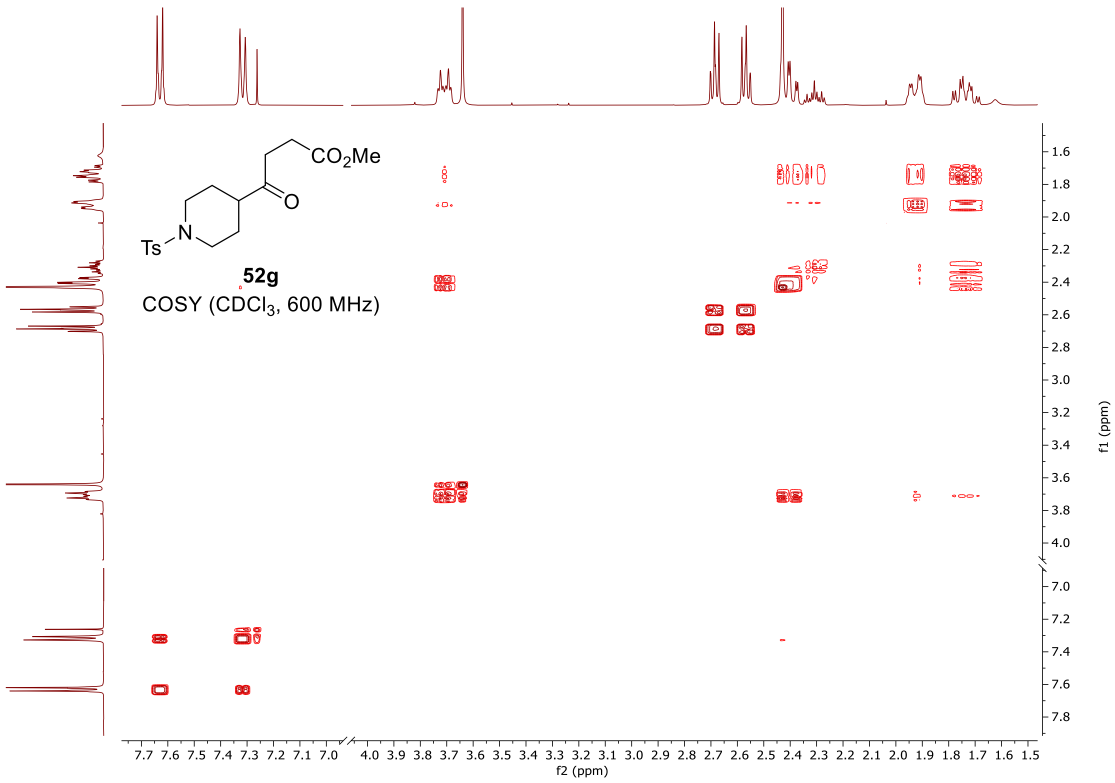

52g
COSY ($CDCl_3$, 600 MHz)
f2 (ppm)
f1 (ppm)

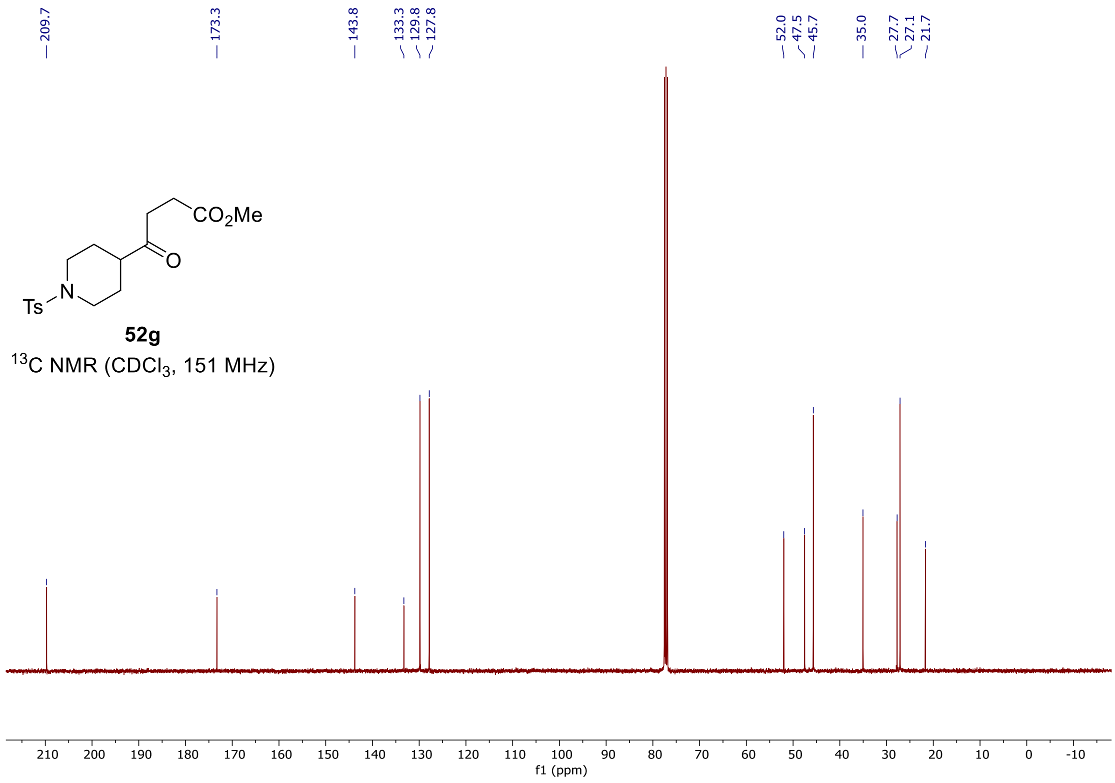
209.7
173.3
143.8
133.3
129.8
127.8
52.0
47.5
45.7
35.0
27.7
27.1
21.7
CO2Me
O
Ts
N
52g
13C NMR (CDCl3, 151 MHz)
f1 (ppm)

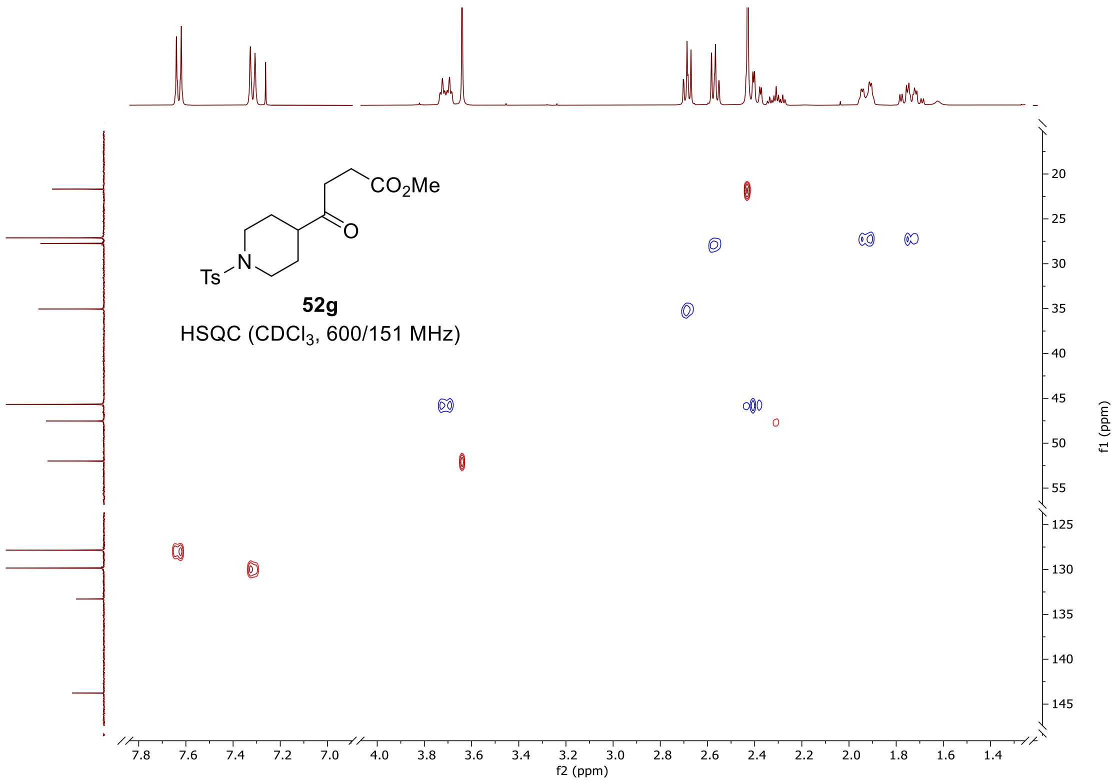
CO2Me
O
Ts
N
52g
HSQC (CDCl3, 600/151 MHz)
f2 (ppm)
f1 (ppm)

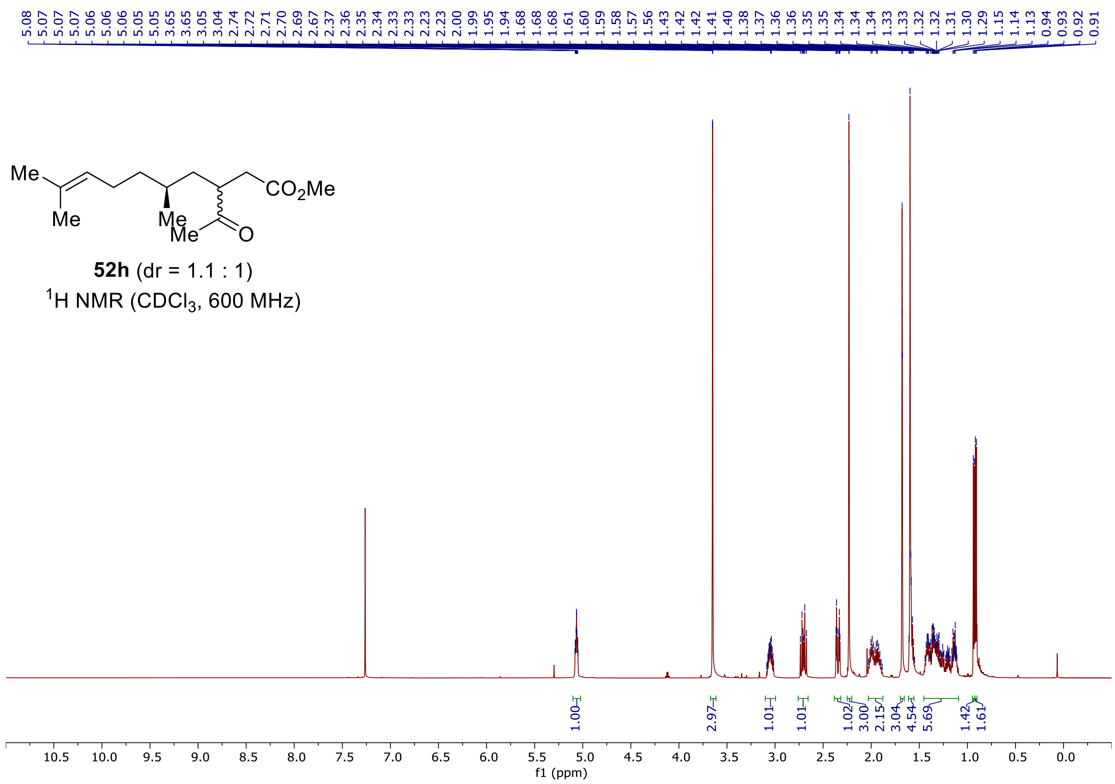
5.08
5.07
5.07
5.06
5.06
5.05
5.05
3.65
3.05
3.04
2.74
2.72
2.71
2.70
2.69
2.67
2.37
2.36
2.35
2.34
2.33
2.33
2.23
2.23
2.00
1.99
1.95
1.94
1.68
1.68
1.61
1.60
1.59
1.58
1.57
1.56
1.43
1.42
1.42
1.41
1.40
1.38
1.37
1.36
1.36
1.35
1.35
1.34
1.34
1.34
1.33
1.33
1.32
1.32
1.31
1.30
1.29
1.15
1.14
1.13
0.94
0.93
0.92
0.91
Me
Me
Me
Me
CO2Me
O
52h (dr = 1.1 : 1)
1H NMR (CDCl3, 600 MHz)
1.00
2.97
1.01
1.01
1.02
3.00
2.15
3.04
4.54
5.69
1.42
1.61
f1 (ppm)

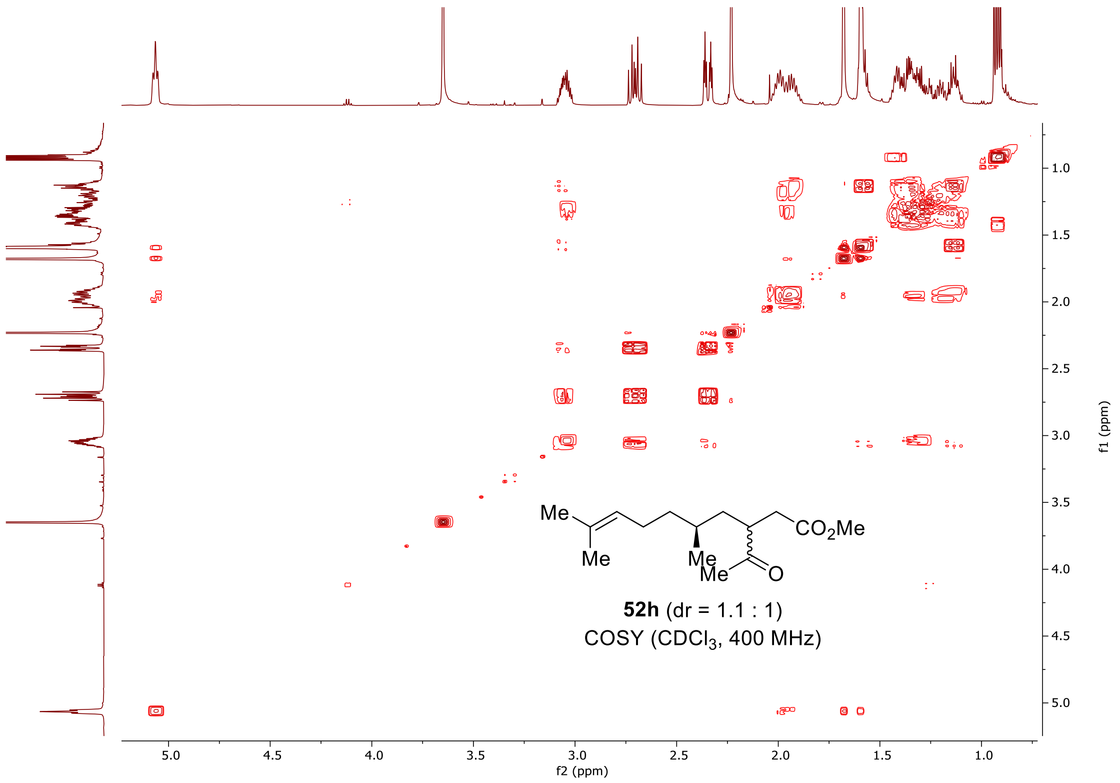
Me
Me
Me
Me
CO2Me
O
52h (dr = 1.1 : 1)
COSY (CDCl3, 400 MHz)
f2 (ppm)
f1 (ppm)

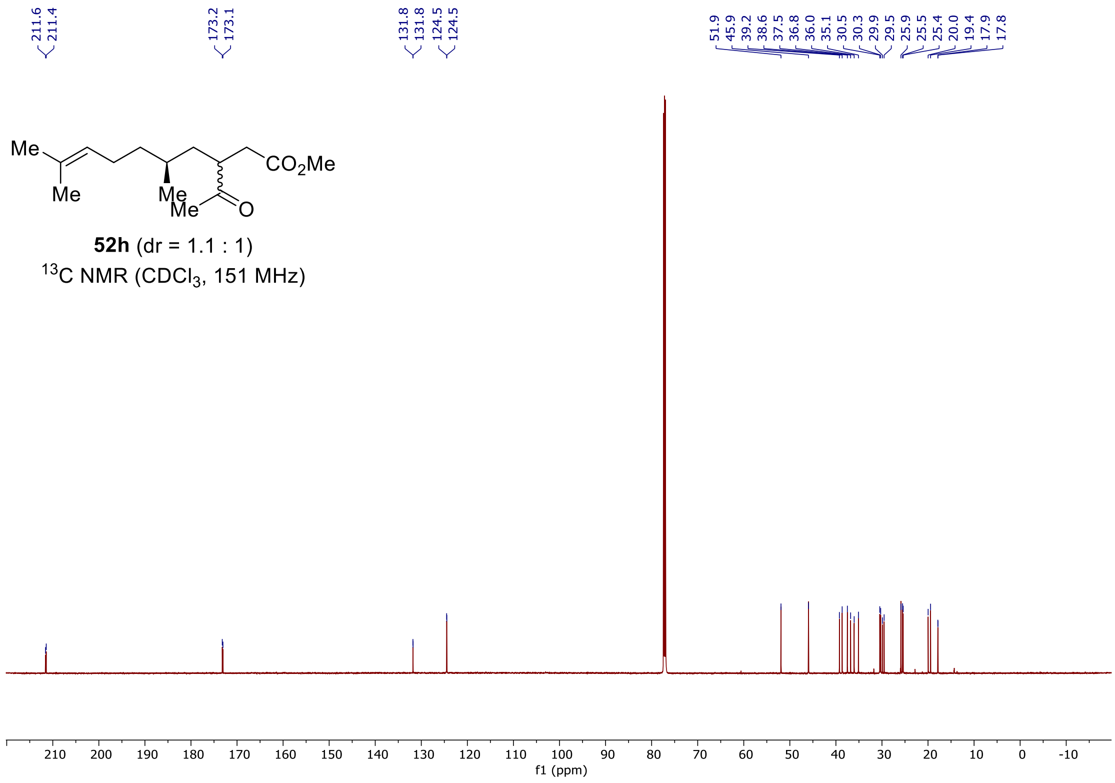

211.6
211.4
173.2
173.1
131.8
131.8
124.5
124.5
51.9
45.9
39.2
38.6
37.5
36.8
36.0
35.1
30.5
30.3
29.9
29.5
25.9
25.5
25.4
20.0
19.4
17.9
17.8
Me
Me
Me
Me
CO_2Me
O
52h (dr = 1.1 : 1)
^{13}C NMR ($CDCl_3$, 151 MHz)
f1 (ppm)

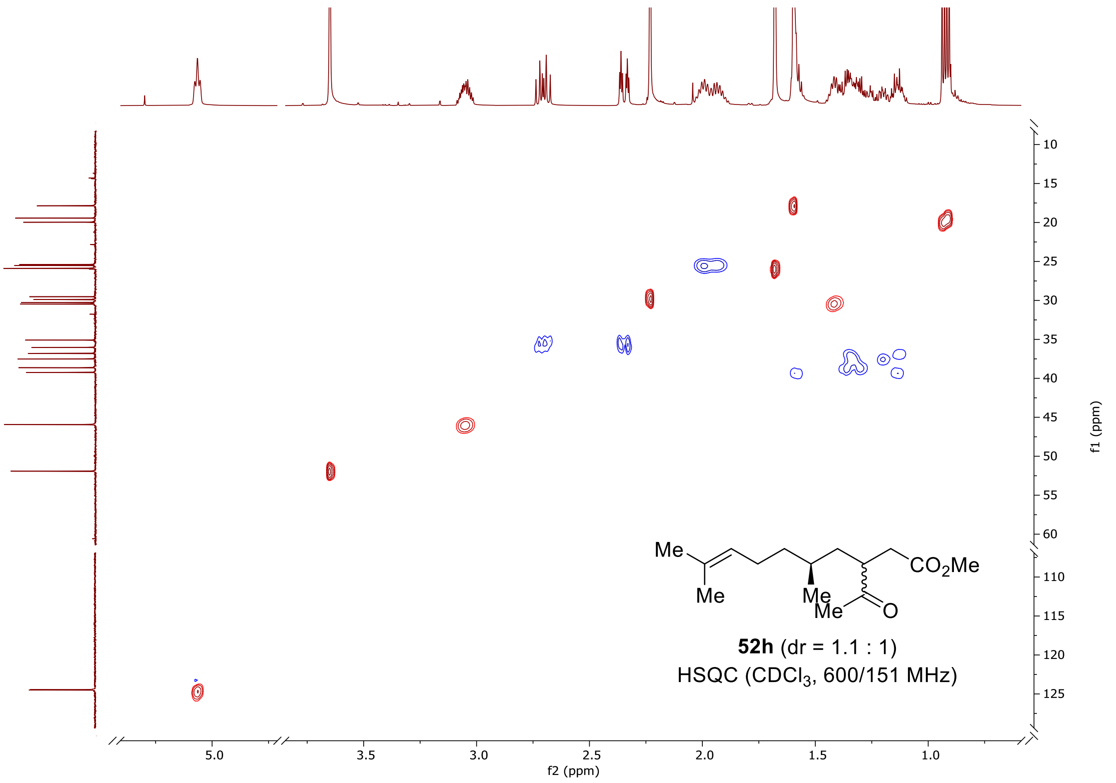

Me
Me
Me
Me
CO_2Me
O
52h (dr = 1.1 : 1)
HSQC ($CDCl_3$, 600/151 MHz)
f2 (ppm)
f1 (ppm)
